# Supplementary material for: Comparison of serum lactate and lactate-derived ratios as prognostic biomarkers in pediatric dengue shock syndrome using supervised machine learning models
Source: PLoS One. 2025 Oct 27;20(10):e0335022. doi: 10.1371/journal.pone.0335022 (PMC12558473; doi:10.1371/journal.pone.0335022)
Supplement: S1 Table — (DOCX) [file pone.0335022.s001.docx]

**Table S1. Variables used in the supervised machine learning models**

These covariables were predetermined based on our clinical knowledge and a literature review.

| **Variable names** | **Description** | **Data types** |
| --- | --- | --- |
| age.year | Patient age by years | Continuous variable |
| sex.female (versus male) | Patient’s sex, Female = 1, Male = 0 | Binary data (1/0) |
| DSS.Decompensated | Severity of dengue shock syndrome (DSS): decompensated DSS = 1;  compensated DSS = 0 | Binary data (1/0) |
| underlying.disease | Presence of underlying diseases accompanied at hospital admission | Binary data (1/0) |
| onset.day.shock | Early onset day of occurrence of dengue shock < day-4 since the manifestation of the first symptoms (vs. late onset of DSS > = day 4 since disease onset) | Binary data (1/0) |
| Res.rate | Respiratory rate (breaths per minute) | Continuous variable |
| Sys.shock.index | Systolic shock index (bpm/mmHg) | Continuous variable |
| severe.bleeding | Critical bleeding in dengue-infected patients | Binary data (1/0) |
| PLT.lower20K | Low platelet count < 20 x 10^9^/L | Binary data (1/0) |
| PLT.transfusion | Patients required platelet transfusion | Binary data (1/0) |
| HCT.peak | The highest value of hematocrit (%) during the first 24h of admission | Continuous variable |
| HCT.nadir | The lowest value of hematocrit (%) during the first 24h of admission | Continuous variable |
| Severe.hepatitis | Severe transminitis defined by WHO Dengue guidelines in 2009, as AST or ALT > 1,000 IU/L | Binary data (1/0) |
| INR | International normalized ratio | Continuous variable |
| AVPU.admission | A = alert, V = verbal response, P = pain response, U = Unresponse  P and U levels coded as 1  A and V levels coded as 0 | Categorical data |
| Albumin.Admission | Serum albumin (g/L) at PICU admission | Continuous variable |
| Lactate.admission | Serum lactate (mmol/L) on PICU admission | Continuous variable |
| HCO3.admission | Serum bicarbonate (mEq/L) on PICU admission | Continuous variable |
| Creatinin | Serum creatinine (µmol/L) | Continuous variable |
| Cumulative.fluid.24h | Cumulative amount of fluid infused (mL/kg) from referral hospital and 24h of admission | Continuous variable |
| VIS.24h.over30 | Vasoactive inotropic score (VIS) levels-during the first 24hours of PICU admission > 30 (units) | Binary data (1/0) |
| LA.admission | Serum lactate to albumin ratio | Continuous variable |
| LB.admission | Serum lactate to bicarbonate ratio | Continuous variable |
| critical_dengue | The composite clinical endpoint including death, mechanical ventilation, dengue-associated PALF and encephalitis | Binary data (1/0) |

DSS, Dengue shock syndrome; PICU, pediatric intensive care unit; WHO, World Health Organization
