# Supplementary material for: Comparison of serum lactate and lactate-derived ratios as prognostic biomarkers in pediatric dengue shock syndrome using supervised machine learning models
Source: PLoS One. 2025 Oct 27;20(10):e0335022. doi: 10.1371/journal.pone.0335022 (PMC12558473; doi:10.1371/journal.pone.0335022)
Supplement: S2 Table — (DOCX) [file pone.0335022.s002.docx]

Supplementary data

**S2 Table.** **Standardized mean differences for covariables assessing distributional balance between outcome groups**

Baseline characteristics are presented according to outcome status, with standardized mean differences reported to indicate balance among groups.

| *Stratified by Composite Endpoint* | | | |
| --- | --- | --- | --- |
| **Covariables** | **Composite endpoint-Yes**  (n = 89) | **Composite endpoint-No**  **(**n = 435) | **SMD** |
| Age, year (mean ± SD) | 6.7 (3.5) | 8.9 (3.6) | 0.611 |
| Female sex, n (%) | 40 (44.9) | 192 (44.1) | 0.016 |
| Underlying diseases, n (%) | 12 (13.5) | 39 (9.0) | 0.143 |
| Decompensated dengue shock, n (%) | 22 (25) | 41 (9.4) | 0.415 |
| Early onset of shock (< 4 days), n (%) | 23 (26) | 47 (11) | 0.396 |
| Severe bleeding, n (%) | 46 (52) | 9 (2.1) | 1.350 |
| Severe transaminitis, n (%) | 52 (58) | 22 (5.1) | 1.399 |
| Respiratory rate (/min), (mean ± SD) | 31 (8) | 25 (5) | 0.870 |
| Systolic shock index, (bpm/mmHg) (mean ± SD) | 1.48 (0.37) | 1.31 (0.29) | 0.517 |
| HCT peak (%), (mean ± SD) | 45.7 (7.4) | 48.7 (5.1) | 0.463 |
| HCT nadir (%), (mean ± SD) | 35.0 (7.4) | 38.7 (4.7) | 0.598 |
| INR, (mean ± SD) | 2.77 (1.96) | 1.32 (0.38) | 1.026 |
| Serum creatinine, µmol/L (mean ± SD) | 66 (43) | 54 (12) | 0.386 |
| Serum albumin, g/dL, (mean ± SD) | 2.1 (0.71) | 2.8 (0.97) | 0.997 |
| Serum lactate, mmol/L, (mean ± SD) | 4.88 (3.74) | 2.48 (1.33) | 0.854 |
| Serum bicarbonate, mEq/L, (mean ± SD) | 16 (4) | 17 (5) | 0.173 |
| LAR, (mean ± SD) | 2.65 (2.45) | 0.93 (0.71) | 0.949 |
| LB ratio, (mean ± SD) | 0.37 (0.39) | 0.23 (0.38) | 0.357 |
| Cumulative fluid infusion from referral hospital and within 24h admission, mL/kg, (mean ± SD) | 221 (117) | 146 (50) | 0.839 |
| Vasoactive inotropic score within first 24h admission > 30, n (%) | 23 (26) | 0 (0) | 0.835 |

**Notes.** SD, standard deviation; SMD, standardized mean difference

Standardized Mean Difference (SMD) > 0.1 indicates imbalance between groups.
