# Supplementary material for: Comparison of serum lactate and lactate-derived ratios as prognostic biomarkers in pediatric dengue shock syndrome using supervised machine learning models
Source: PLoS One. 2025 Oct 27;20(10):e0335022. doi: 10.1371/journal.pone.0335022 (PMC12558473; doi:10.1371/journal.pone.0335022)
Supplement: S3 Table — (DOCX) [file pone.0335022.s003.docx]

**Full predictive model specification**

**S3 Table. The unadjusted associations between the covariables and composite endpoint**

| **Variables** | **Statistics** | **OR** | **95% CI** | **p-value** |
| --- | --- | --- | --- | --- |
| Lactate (+1 mmol/L) | 2.3 (1.7-3.2) | 1.54 | 1.36 – 1.74 | < 0.001 |
| LAR (+1 unit) | 0.86 (0.61-1.30) | 2.84 | 2.14 – 3.78 | < 0.001 |
| LB (+1 unit) | 0.14 (0.09-0.23) | 2.05 | 1.23 – 3.41 | < 0.01 |
| Age (years) | 8.7 (6-11) | 0.85 | 0.79-0.91 | < 0.001 |
| Female patients, n (%) | 232 (44.3) | 1.03 | 0.65-1.63 | 0.89 |
| Underlying diseases, n (%) | 51 (9.7) | 1.58 | 0.79-3.16 | 0.19 |
| Early onset of dengue shock < 4 days, n (%) | 70 (13.4) | 2.88 | 1.64-5.05 | < 0.001 |
| Decompensated shock, n (%) | 63 (12) | 3.16 | 1.77-5.63 | < 0.001 |
| Respiratory rate (/min) | 24 (22-28) | 1.13 | 1.09-1.18 | < 0.001 |
| Systolic shock index (bpm/mmHg) | 1.3 (1.11-1.5) | 5.07 | 2.31-11.1 | < 0.001 |
| Pain and Unresponsive levels on AVPU scale, n (%) | 32 (6.1) | 232 | 31.1-1731 | < 0.001 |
| Severe bleeding, n (%) | 55 (10.5) | 50.5 | 23.2-110.2 | < 0.001 |
| Severe transaminitis, n (%) | 74(14.1) | 26.4 | 14.5-48.1 | < 0.001 |
| Peak hematocrit (%) | 48 (45-52) | 0.92 | 0.88-0.95 | < 0.001 |
| Nadir hematocrit (%) | 39 (35-41) | 0.89 | 0.85-0.93 | < 0.001 |
| Serum creatinine (µmol/L) | 53 (45-61) | 1.02 | 1.01-1.04 | < 0.001 |
| Serum albumin (g/dL) | 2.8 (2.0-3.5) | 0.89 | 0.86-0.92 | < 0.001 |
| Serum bicarbonate (mEq/L) | 17.1 (14.7-19.5) | 0.97 | 0.93-1.01 | 0.18 |
| Low platelet count (< 20 x 10^9^/L), n (%) | 159 (30.3) | 0.99 | 0.61-1.64 | 0.99 |
| Platelet transfusion, n (%) | 88 (16.8) | 36.1 | 19.8-65.9 | < 0.001 |
| International normalized ratio | 1.3 (1.2-1.6) | 16.6 | 9.1-30.5 | < 0.001 |
| Cumulative amount of fluid infused from referral hospitals and during 24h-admission (mL/kg) | 143 (110-190) | 1.01 | 1.01-1.02 | < 0.001 |

Statistics are presented as median (interquartile range) for continuous variables and n (%) for categorical variables. CI, 95% confidence interval; OR, odds ratio; LAR, Lactate-to-albumin ratio; LB, Lactate-to-bicarbonate ratio

Notably, vasoactive inotropic score (VIS), particularly high VIS values (> 30) and the log2-VIS score, showed extreme values in the statistical analysis, indicating near-perfect prediction of the study outcome (p < 0.001). All covariables were selected a priori based on clinical knowledge and review of the medical literature (*references [1-5] below*). Although some covariables were not statistically significant, they were retained in the full model due to their clinical relevance and pathophysiological importance.

**References**

[1] Thanh NT, Luan VT, Viet DC, Tung TH, Thien V. A machine learning-based risk score for prediction of mechanical ventilation in children with dengue shock syndrome: A retrospective cohort study. PLoS One. 2024 Dec 6;19(12):e0315281. doi: 10.1371/journal.pone.0315281. PMID: 39642139; PMCID: PMC11623794.

[2] Nguyen Tat T, Vo Hoang-Thien N, Nguyen Tat D, Nguyen PH, Ho LT, Doan DH, et al. Prognostic values of serum lactate-to-bicarbonate ratio and lactate for predicting 28-day in-hospital mortality in children with dengue shock syndrome. Medicine (Baltimore). 2024 Apr 26;103(17):e38000. doi: 10.1097/MD.0000000000038000. PMID: 38669370; PMCID: PMC11049702.

[3] Vo LT, Do VC, Trinh TH, Nguyen TT. In-Hospital Mortality in Mechanically Ventilated Children With Severe Dengue Fever: Explanatory Factors in a Single-Center Retrospective Cohort From Vietnam, 2013-2022. Pediatr Crit Care Med. 2025 Jun 1;26(6):e796-e805. doi: 10.1097/PCC.0000000000003728. Epub 2025 Mar 19. PMID: 40105396; PMCID: PMC12133049.

[4] Nguyen TT, Ngo PT, Vo LT. Predicting the risk of mortality in children with dengue-induced hepatitis admitted to the paediatric intensive care unit. World J Crit Care Med. 2024 Dec 9;13(4):98862. doi: 10.5492/wjccm.v13.i4.98862. PMID: 39655306; PMCID: PMC11577541.

[5] Huy BV, Toàn NV. Prognostic indicators associated with progresses of severe dengue. PLoS One. 2022 Jan 5;17(1):e0262096. doi: 10.1371/journal.pone.0262096. PMID: 34986174; PMCID: PMC8730386.
