# Supplementary figures and images for: Comparison of serum lactate and lactate-derived ratios as prognostic biomarkers in pediatric dengue shock syndrome using supervised machine learning models
Source: PLoS One. 2025 Oct 27;20(10):e0335022. doi: 10.1371/journal.pone.0335022 (PMC12558473; doi:10.1371/journal.pone.0335022)

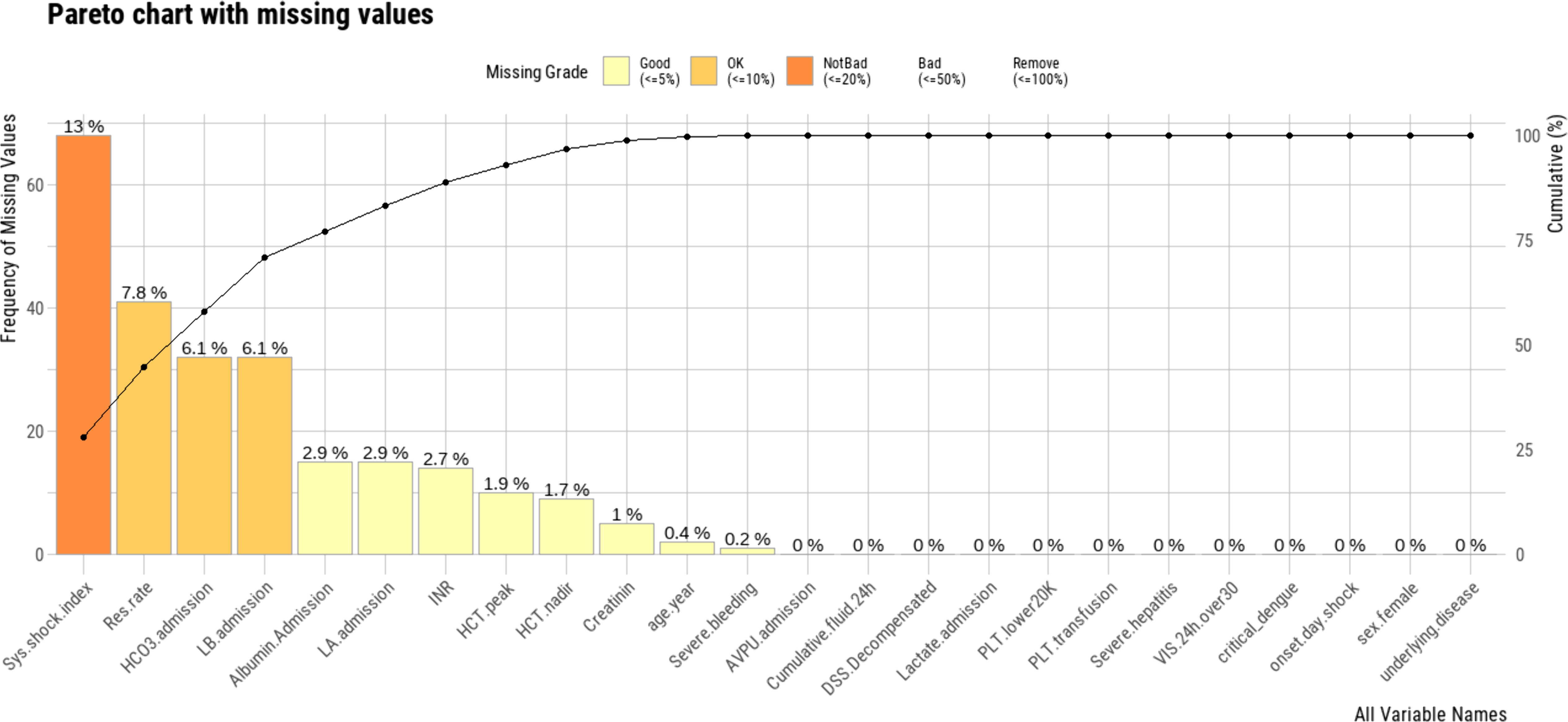

Supplement: S1 Fig — (TIF) [file pone.0335022.s004.tif]

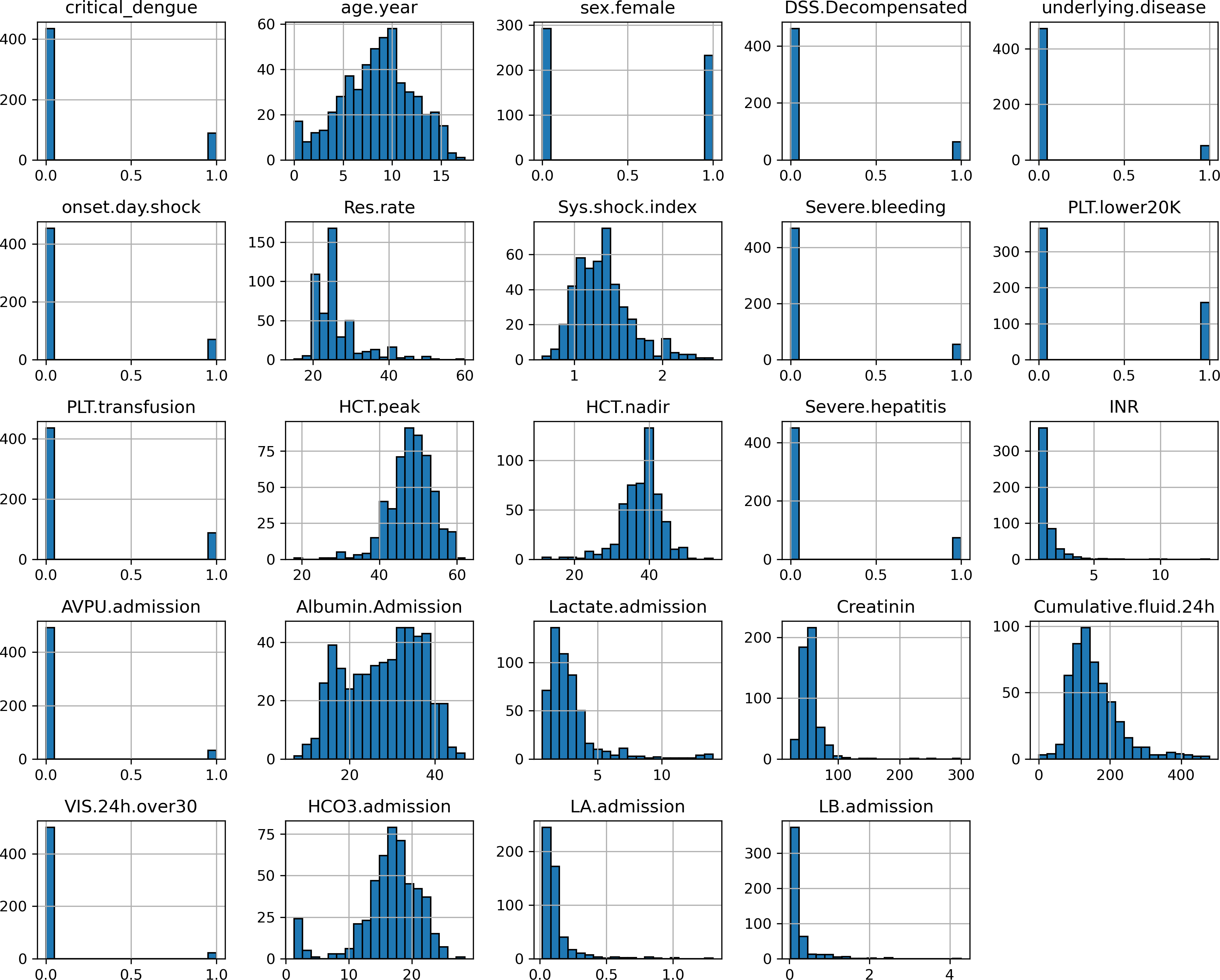

Supplement: S2 Fig — (TIF) [file pone.0335022.s005.tif]
