# Supplementary material for: Comparison of serum lactate and lactate-derived ratios as prognostic biomarkers in pediatric dengue shock syndrome using supervised machine learning models
Source: PLoS One. 2025 Oct 27;20(10):e0335022. doi: 10.1371/journal.pone.0335022 (PMC12558473; doi:10.1371/journal.pone.0335022)

**The least absolute shrinkage and selection operator (LASSO) method**

The least absolute shrinkage and selection operator penalization was applied to optimize the model dimensionality by shrinking the regression coefficients through the penalty parameter (λ). Coefficient trajectories of all predefined covariates across log(λ) values. The optimal λ value was determined using the one-standard-error criterion to select the most parsimonious model. These data are illustrated in the following plots:


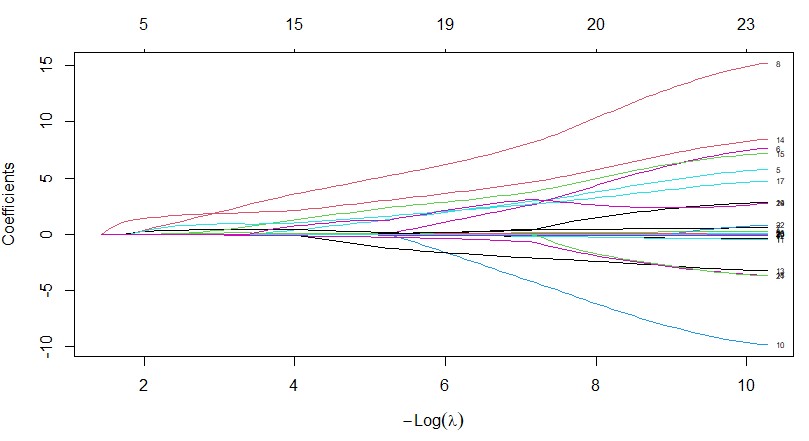


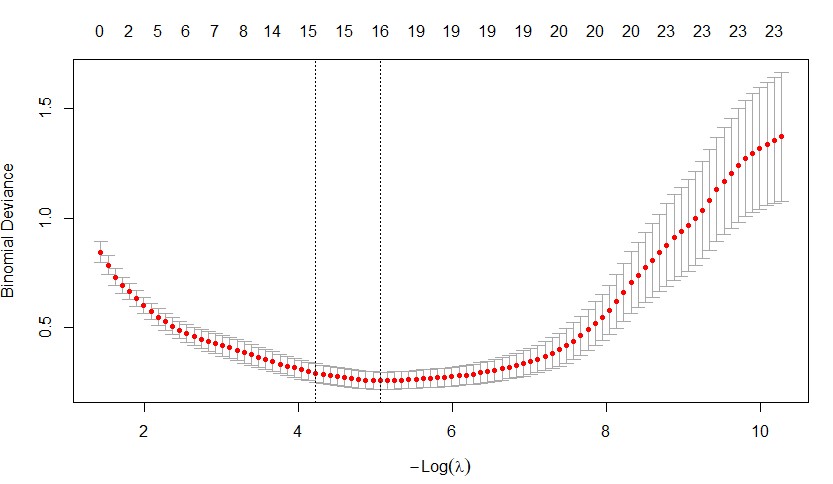

Supplement: S1 File — (DOCX) [file pone.0335022.s006.docx]
