# Supplementary material for: Comparison of serum lactate and lactate-derived ratios as prognostic biomarkers in pediatric dengue shock syndrome using supervised machine learning models
Source: PLoS One. 2025 Oct 27;20(10):e0335022. doi: 10.1371/journal.pone.0335022 (PMC12558473; doi:10.1371/journal.pone.0335022)
Supplement: S2 File — (DOCX) [file pone.0335022.s007.docx]

**The** **study analysis flowchart for predictive model development for the composite endpoint in children with dengue shock syndrome admitted to the pediatric intensive care unit**

**Dengue cohort 2013-2022**

Approximately 2,000 patients with dengue shock syndrome

**Composite endpoint** included Death, Mechanical Ventilation Requirement, Dengue-associated Acute Liver Failure and Encephalitis

**Shapley Addictive Explanations (SHAP)**

Determine the most key features for the predefined composite endpoint

**Model performance assessment:**

AUC, Sensitivity, Specificity, Precision,

F1 score, Accuracy

Identify the optimal predictive model

**Supervised models**

Logistic regression, Random Forest, Support Vector Machine, k-Nearest Neighbors, AdaBoost, XGBoost, Naïve Bayes

**Variable selection process**

Based on the clinical knowledge, medical literature, AIC and LASSO methods

23 most significant predictors identified

**Split dataset**

Training set (80%) and Test set (20%)

5-fold cross validation

**Data Pre-processing**

Eliminate unnecessary variables

Missing data of interest

**Study population**

N = 524 patients with total 75 variables
