# Supplementary material for: Comparison of serum lactate and lactate-derived ratios as prognostic biomarkers in pediatric dengue shock syndrome using supervised machine learning models
Source: PLoS One. 2025 Oct 27;20(10):e0335022. doi: 10.1371/journal.pone.0335022 (PMC12558473; doi:10.1371/journal.pone.0335022)
Supplement: S2 Appendix — (DOCX) [file pone.0335022.s009.docx]

S2_Appendix

**All statistical analysis code with R (version 4.5.0) and Python (Anaconda Distribution version 3.0)**

Datasets which were analyzed including:

1. Full dataset - S1_Appendix_Dataset
2. Multiple imputation dataset - LALB.multiple.imputation

These datasets were submitted (**S1_Appendix)**.

**A summary of all predefined covariables and outcomes in the analysis:**

These covariables were predetermined based on our clinical knowledge and a literature review.

| **Variable names** | **Description** | **Data types** |
| --- | --- | --- |
| age.year | Patient age by years | Continuous variable |
| sex.female (versus male) | Patient’s sex, Female = 1, Male = 0 | Binary data (1/0) |
| DSS.Decompensated | Severity of dengue shock syndrome (DSS): decompensated DSS = 1;  compensated DSS = 0 | Binary data (1/0) |
| underlying.disease | Underlying diseases accompanied at hospital admission | Binary data (1/0) |
| onset.day.shock | Early onset day of occurrence of dengue shock < day-4 since the manifestation of the first symptoms (vs. late onset of DSS > = day 4 since disease onset) | Binary data (1/0) |
| Res.rate | Respiratory rate (breaths per minute) | Continuous variable |
| Sys.shock.index | Systolic shock index (bpm/mmHg) | Continuous variable |
| severe.bleeding | Critical bleeding in dengue-infected patients | Binary data (1/0) |
| PLT.lower20K | Low platelet count < 20 x 10^9^/L | Binary data (1/0) |
| PLT.transfusion | Patients required platelet transfusion | Binary data (1/0) |
| HCT.peak | The highest value of hematocrit (%) during the first 24h of admission | Continuous variable |
| HCT.nadir | The lowest value of hematocrit (%) during the first 24h of admission | Continuous variable |
| Severe.hepatitis | Severe transminitis defined by WHO Dengue guidelines in 2009, as AST or ALT > 1,000 IU/L | Binary data (1/0) |
| INR | International normalized ratio | Continuous variable |
| AVPU.admission | A = alert, V = verbal response, P = pain response, U = Unresponse  P and U levels coded as 1  A and V levels coded as 0 | Categorical data |
| Albumin.Admission | Serum albumin (g/L) at PICU admission | Continuous variable |
| Lactate.admission | Serum lactate (mmol/L) on PICU admission | Continuous variable |
| HCO3.admission | Serum bicarbonate (mEq/L) on PICU admission | Continuous variable |
| Creatinin | Serum creatinine (µmol/L) | Continuous variable |
| Cumulative.fluid.24h | Cumulative amount of fluid infused (mL/kg) from referral hospital and 24h of admission | Continuous variable |
| VIS.24h.over30 | Vasoactive inotropic score (VIS) levels-during the first 24hours of PICU admission > 30 (units) | Binary data (1/0) |
| LA.admission | Serum lactate to albumin ratio | Continuous variable |
| LB.admission | Serum lactate to bicarbonate ratio | Continuous variable |
| critical_dengue | The composite clinical endpoint including death, mechanical ventilation, dengue-associated PALF and encephalitis | Binary data (1/0) |

*Full code for the study analysis*:

1. **Baseline characteristics of study participants**

**Table 1**- **Baseline clinical and laboratory characteristics of study participants on admission and clinical outcomes at discharge**

# R code

# Descriptive analysis

rm(list=ls()) ### Clear the working environments

### Import original dataset: S1_Appendix

# Install package if not already installed

#install.packages("readxl")

library(readxl)

# Import Excel file in R

df <- read_excel("C:/Users/HP/Desktop/S1_Appendix.xlsx", sheet = "S1_Appendix_Dataset") ## xlsx excel

### Factor covariables

df$sex <- as.factor(df$sex)

df$DSS.grade <- as.factor(df$DSS.grade)

df$underlying.disease <- as.factor(df$underlying.disease)

df$onset.day.shock <- as.factor(df$onset.day.shock)

df$Severe.bleeding <- as.factor(df$Severe.bleeding)

df$PLT.lower20K <- as.factor(df$PLT.lower20K)

df$PLT.transfusion <- as.factor(df$PLT.transfusion)

df$Severe.hepatitis <- as.factor(df$Severe.hepatitis)

df$AVPU.Pain.Unresponse <- as.factor(df$AVPU.Pain.Unresponse)

### Factor categorical variables:

df$death <- as.factor(df$death)

df$MV <- as.factor(df$MV)

df$PALF <- as.factor(df$PALF)

df$Encephalitis.dengue <- as.factor(df$Encephalitis.dengue)

#### Data preparation:

# Structure of dataset (variable types, first few entries)

str(df)

# Exploring and understanding data

head(df)

tail(df)

dim(df)

library(tidyverse)

glimpse(df)

# Check missing

library(rms)

na.patterns = naclus(df)

plot(na.patterns)

########### Descriptive Analysis R packages ##################

#library(devtools)

#devtools::install_github("isubirana/compareGroups")

#install.packages("compareGroups")

library(compareGroups)

#install.packages("table1")

library(table1)

#install.packages("DescTools")

library(DescTools)

**Figure 1**- The study flowchart

# Descriptive analysis

# R code

summary(df)

## TABLE 1- Baseline characteristic and comparison tables:

## Basic summary statistics (df):

summary(df)

## 1-1 Baseline characteristics of study participants

library(table1)

table1 (~ age.year + sex + DSS.grade + bmi+underlying.disease +

onset.day.shock + Severe.bleeding + PLT.lower20K + PLT.transfusion +

AVPU.Pain.Unresponse + Res.rate + Sys.shock.index + Res.rate +

WBC+Hb+HCT.peak + HCT.nadir +PLT + PLT.transfusion+INR + Creatinin + AST+ALT+Severe.hepatitis+ Albumin.Admission.g.dL + Lactate.admission + HCO3.admission + LA.admission + LB.admission + pH + PaCO2 + PO2 + FiO2+HCO3.admission + Cumulative.fluid.24h+VIS.admission + MV+PALF+Encephalitis.dengue+death+critical_dengue, data = df)

### Factor outcome variable (composite endpoint = critical_dengue)

df$critical_dengue<-as.factor(df$critical_dengue)

# Generate baseline characteristics stratified by critical_dengue

table1(~ age.year + sex + DSS.grade + bmi+underlying.disease +

onset.day.shock + Severe.bleeding + PLT.lower20K + PLT.transfusion +

AVPU.Pain.Unresponse + Res.rate + Sys.shock.index + Res.rate +

WBC+Hb+HCT.peak + HCT.nadir +PLT + PLT.transfusion+INR + Creatinin + AST+ALT+Severe.hepatitis+

Albumin.Admission.g.dL + Lactate.admission + HCO3.admission + LA.admission + LB.admission +

pH + PaCO2 + PO2 + FiO2+HCO3.admission+

Cumulative.fluid.24h+VIS.admission+MV+PALF+Encephalitis.dengue+death | critical_dengue,

data = df)

### 1-2 Comparison tables with p-values

### Compare groups by outcome variables (critical_dengue = composite endpoint)

library(compareGroups)

t = compareGroups(critical_dengue~age.year + sex + DSS.grade + bmi+underlying.disease +

onset.day.shock + Severe.bleeding + PLT.lower20K + PLT.transfusion +

AVPU.Pain.Unresponse + Res.rate + Sys.shock.index + Res.rate +

WBC+Hb+HCT.peak + HCT.nadir +PLT + PLT.transfusion+INR + Creatinin +AST + ALT+Severe.hepatitis+

Albumin.Admission.g.dL + Lactate.admission + HCO3.admission + LA.admission + LB.admission + pH + PaCO2 + PO2 + FiO2+HCO3.admission+ Cumulative.fluid.24h+VIS.admission+MV+PALF+Encephalitis.dengue+death,data=df)

createTable(t)

### 1-3-Baseline characteristics table stratified by study outcome with standardized mean differences (SMD)

## **S2 Table.** **Standardized mean differences (SMD) for covariables assessing distributional balance between outcome groups**

# Load libraries

library(dplyr)

library(tableone)

# Step 1: Select variables into df1 dataframe

df1 <- df %>%

select(

age.year, sex, DSS.grade, bmi, underlying.disease,

onset.day.shock, Severe.bleeding, PLT.lower20K, PLT.transfusion,

AVPU.Pain.Unresponse, Res.rate, Sys.shock.index,

WBC, Hb, HCT.peak, HCT.nadir, PLT, INR, Creatinin, AST, ALT, Severe.hepatitis,

Albumin.Admission.g.dL, Lactate.admission, HCO3.admission, LA.admission, LB.admission,

pH, PaCO2, PO2, FiO2,

Cumulative.fluid.24h, VIS.admission, MV, PALF, Encephalitis.dengue, death, critical_dengue

)

# Step 2: Define outcome variable for stratification

df1$CompositeEndpoint <- df1$critical_dengue

# Step 3: Define variables of interest (matching df1 column names)

vars <- c(

"age.year", "sex", "DSS.grade", "bmi", "underlying.disease",

"onset.day.shock", "Severe.bleeding", "PLT.lower20K", "PLT.transfusion",

"AVPU.Pain.Unresponse", "Res.rate", "Sys.shock.index",

"WBC", "Hb", "HCT.peak", "HCT.nadir", "PLT", "INR", "Creatinin",

"AST", "ALT", "Severe.hepatitis", "Albumin.Admission.g.dL",

"Lactate.admission", "HCO3.admission", "LA.admission", "LB.admission",

"pH", "PaCO2", "PO2", "FiO2", "Cumulative.fluid.24h", "VIS.admission",

"MV", "PALF", "Encephalitis.dengue", "death"

)

# Step 4: Identify categorical variables

catVars <- c(

"sex", "DSS.grade", "underlying.disease", "Severe.bleeding",

"PLT.lower20K", "PLT.transfusion", "AVPU.Pain.Unresponse",

"Severe.hepatitis", "MV", "PALF", "Encephalitis.dengue", "death"

)

# Step 5: Create baseline table stratified by CompositeEndpoint

tab1 <- CreateTableOne(vars = vars, strata = "CompositeEndpoint",

data = df1, factorVars = catVars, test = FALSE)

# Step 6: Print table with SMDs

print(tab1, smd = TRUE)

####

1. **Sensitivity analysis**

**# Complete case data analysis**

**Table 2**- Area under the receiver operating characteristic curve for serum lactate, lactate-to-albumin and lactate-to-bicarbonate ratios, and cut-off points for composite clinical endpoint among children with dengue shock syndrome

# R code

rm(list=ls()) #### Clear all currently working commands

### Import original dataset: S1_Appendix

# Install package if not already installed

# install.packages("readxl")

library(readxl)

# Import Excel file in R

# S1_Appendix <- read.csv("C:/Users/HP/Desktop/S1_Appendix.csv") ## csv file

S1_Appendix <- read_excel("C:/Users/HP/Desktop/S1_Appendix.xlsx", sheet = "S1_Appendix_Dataset") ## xlsx excel file

head(S1_Appendix,3)

### Create a subset data (named rocdata) of biomarkers for analysis

library(dplyr)

library(tidyverse)

rocdata = S1_Appendix %>% select(critical_dengue,Lactate.admission,LA.admission,LB.admission)

head(rocdata,3)

# --- Load required packages ---

library(readxl)

library(dplyr)

library(ggplot2)

library(pROC)

# --- Import Excel file ---

S1_Appendix <- read_excel(

"C:/Users/HP/Desktop/S1_Appendix.xlsx",

sheet = "S1_Appendix_Dataset"

)

# --- Quick check of the first rows ---

head(S1_Appendix, 3)

names(S1_Appendix)

# --- Create ROC dataset (make sure column names match exactly) ---

rocdata <- S1_Appendix %>%

select(critical_dengue,Lactate.admission, LA.admission, LB.admission)

head(rocdata, 3)

### Libraries required:

library(ggplot2)

library(pROC)

## Study Outcome = Composite endpoint (critical_dengue)

### 2-1. Lactate on PICU admission

pROC_obj.lactate.admission <- roc(rocdata$ critical_dengue,rocdata$Lactate.admission,

smoothed = TRUE,

# arguments for ci

ci=TRUE, ci.alpha=0.9, stratified=FALSE,

# arguments for plot

plot=TRUE, auc.polygon=TRUE, max.auc.polygon=TRUE, grid=TRUE,

print.auc=TRUE, show.thres=TRUE)

sens.ci <- ci.se(pROC_obj.lactate.admission)

plot(sens.ci, type="shape", col="lightblue")

## Warning in plot.ci.se(sens.ci, type = "shape", col = "lightblue"): Low

## definition shape.

plot(sens.ci, type="bars")

### P value - Logistic Regression

model1<-glm(critical_dengue~Lactate.admission,family=binomial,data=rocdata)

summary(model1)

library(epiDisplay)#### 95% CI of odd ratio (OR)

logistic.display(model1)

### Other ROC package for calculating Youden Index

library(Epi)

ROC(form = critical_dengue~Lactate.admission, data=rocdata, plot = "ROC", lwd=2)

##### 2-2 Ratio Lactate/Albumin (LAR) at PICU admission

pROC_obj.LAratio.24h <- roc(rocdata$critical_dengue,rocdata$LA.admission,

smoothed = TRUE,

# arguments for ci

ci=TRUE, ci.alpha=0.9, stratified=FALSE,

# arguments for plot

plot=TRUE, auc.polygon=TRUE, max.auc.polygon=TRUE, grid=TRUE,

print.auc=TRUE, show.thres=TRUE)

sens.ci <- ci.se(pROC_obj.ratio.24h)

plot(sens.ci, type="shape", col="lightblue")

## Warning in plot.ci.se(sens.ci, type = "shape", col = "lightblue"): Low

## definition shape.

plot(sens.ci, type="bars")

### P value

model2<-glm(critical_dengue~LA.admission,family=binomial,data=rocdata)

summary(model2)

library(epiDisplay) ### 95% CI of odd ratio (OR)

logistic.display(model2)

### Other ROC package for calculating Youden Index

library(Epi)

ROC(form = critical_dengue~LA.admission, data=rocdata, plot = "ROC", lwd=2)

### 2-3. Ratio Lactate/HCO3 (L/B) on PICU admission

pROC_obj.LBratio.admission <- roc(rocdata$critical_dengue,rocdata$LB.admission,

smoothed = TRUE,

# arguments for ci

ci=TRUE, ci.alpha=0.9, stratified=FALSE,

# arguments for plot

plot=TRUE, auc.polygon=TRUE, max.auc.polygon=TRUE, grid=TRUE,

print.auc=TRUE, show.thres=TRUE)

sens.ci <- ci.se(pROC_obj.LBratio.admission)

plot(sens.ci, type="shape", col="lightblue")

## Warning in plot.ci.se(sens.ci, type = "shape", col = "lightblue"): Low

## definition shape.

plot(sens.ci, type="bars")

### P value

model3<-glm(critical_dengue~ LB.admission,family=binomial,data=rocdata)

summary(model3)

library(epiDisplay)#### 95% CI of odd ratio (OR)

logistic.display(model3)

### Other ROC package for calculating Youden Index

library(Epi)

ROC(form = critical_dengue ~ LB.admission, data=rocdata, plot = "ROC", lwd=2)

##

# Determine CUT-OFF points (Youden Index Criteria) and 95% CI for metrics (Sensitivity, Spec, LR+, LR-)

rm(list=ls()) #### Remove all working commands

# Import original dataset: S1_Appendix

# install.packages("readxl")

library(readxl)

# Import Excel file in R

# S1_Appendix <- read.csv("C:/Users/HP/Desktop/S1_Appendix.csv") ## csv file

S1_Appendix <- read_excel("C:/Users/HP/Desktop/S1_Appendix.xlsx", sheet = "S1_Appendix_Dataset") ## xlsx excel file

head(S1_Appendix,3)

library(dplyr)

# Create a subset data (rocdata) of biomarkers:

rocdata = S1_Appendix %>% select(critical_dengue,Lactate.admission,LA.admission,LB.admission)

head(rocdata,3)

# Factor clinical outcomes # Required for cutpointr package

rocdata$ critical_dengue <- as.factor(rocdata$ critical_dengue)

# Determine cut-off points

library(cutpointr)

library(pROC)

# 2-4 Blood Lactate on admission:

cp1 <- cutpointr(rocdata$Lactate.admission, rocdata$critical_dengue, pos_class = "1", neg_class = "0", direction = ">=",

method = maximize_metric, metric = sum_sens_spec,na.rm = TRUE)

summary(cp1)

plot(cp1)

###

# 2-5 Biomarker LAR

library(pROC)

library(cutpointr)

# --- Ensure outcome is numeric (0/1) --- ### Important step in ROC analysis

rocdata <- rocdata %>%

mutate(critical_dengue = as.numeric(as.character(critical_dengue))) # if factor/char, convert to numeric

# --- Cutpointr ---

cp2 <- cutpointr(

x = rocdata$LA.admission,

class = rocdata$critical_dengue,

pos_class = 1, # now numeric, so use 1 and 0 (not quoted)

neg_class = 0,

direction = ">=",

method = maximize_metric,

metric = sum_sens_spec,

na.rm = TRUE

)

summary(cp2)

plot(cp2)

####

# set up Cutoff for LAR: LAR = 1.25

# --- Define a Predefined Cutoff Point ---

# Let's say, based on previous research or clinical guidelines,

library(pROC)

# a predictor value above 0.125 is considered positive.

cutoff_point <- 1.25

# --- Calculate Sensitivity and Specificity ---

# Convert predictor to binary based on the cutoff point

predicted_outcome <- as.factor(ifelse(rocdata$LA.admission >= cutoff_point, 1, 0))

# Create a confusion matrix

conf_matrix <- table(Predicted = predicted_outcome, Actual = rocdata$critical_dengue) ## Predicted vs outcome data

print("Confusion Matrix:")

print(conf_matrix)

# Extract values from confusion matrix

# Assuming '1' is the positive class and '0' is the negative class

true_positive <- conf_matrix["1", "1"]

false_negative <- conf_matrix["0", "1"]

false_positive <- conf_matrix["1", "0"]

true_negative <- conf_matrix["0", "0"]

# Calculate Sensitivity (True Positive Rate)

#Sensitivity = TP / (TP + FN)

sensitivity <- true_positive / (true_positive + false_negative)

# Calculate Specificity (True Negative Rate)

# Specificity = TN / (TN + FP)

specificity <- true_negative / (true_negative + false_positive)

cat("\n--- Metrics at Predefined Cutoff ---\n")

cat("Cutoff Point:", cutoff_point, "\n")

cat("Sensitivity:", round(sensitivity, 4), "\n")

cat("Specificity:", round(specificity, 4), "\n")

# Calculate Accuracy

accuracy <- (true_positive + true_negative) /

(true_positive + true_negative + false_positive + false_negative)

cat("Accuracy:", round(accuracy, 4), "\n")

##### 95% Confidence interval for LAR metrics:

# Confusion matrix and outputs Sensitivity, Specificity, LR+, LR− with confidence intervals.

#Likelihood Ratios (LR+ and LR−) with 95% CIs, we can use the epiR package (epi.tests()

#install.packages("epiR")

library(epiR)

library(pROC)

### Additional analysis for likelihood ratios (LR + AND LR -)

### set up Cutoff point for LAR = 1.25

# --- Define a Predefined Cutoff Point ---

cutoff_point <- 1.25

# --- Calculate Predicted Outcome ---

predicted_outcome <- as.factor(ifelse(rocdata$LA.admission >= cutoff_point, 1, 0))

# Ensure actual outcome is factor with 0/1 levels

rocdata$critical_dengue <- factor(rocdata$critical_dengue, levels = c(0,1))

# --- Confusion Matrix ---

conf_matrix <- table(Predicted = predicted_outcome, Actual = rocdata$critical_dengue)

print("Confusion Matrix:")

print(conf_matrix)

# --- Use epiR to calculate metrics with 95% CI ---

# epi.tests expects a matrix with columns = actual (disease status), rows = test result

# Format: [Test Positive, Test Negative] x [Disease Positive, Disease Negative]

epi_res <- epi.tests(conf_matrix)

print(epi_res)

### 2-6 LB ratio

library(pROC)

library(cutpointr)

cp3 <- cutpointr(rocdata$LB.admission, rocdata$critical_dengue, pos_class = "1", neg_class = "0", direction = ">=", method = maximize_metric, metric = sum_sens_spec,na.rm = TRUE)

summary(cp3)

plot(cp3)

###

### set up cutoffs for LB: LB = 0.20

### Includes confusion matrix, metrics, bootstrap CIs,

### AUC with 95% CI, and ROC plot.

library(pROC)

# --- Define Predefined Cutoff for LB ratio ---

cutoff_point <- 0.20 # ✅

# Predicted class at cutoff

predicted_outcome <- ifelse(rocdata$LB.admission >= cutoff_point, 1, 0)

predicted_outcome <- factor(predicted_outcome, levels = c(0, 1))

actual_outcome <- factor(rocdata$critical_dengue, levels = c(0, 1))

# --- Confusion Matrix ---

conf_matrix <- table(Predicted = predicted_outcome, Actual = actual_outcome)

print("Confusion Matrix:")

print(conf_matrix)

# Extract values

true_positive <- conf_matrix["1", "1"]

false_negative <- conf_matrix["0", "1"]

false_positive <- conf_matrix["1", "0"]

true_negative <- conf_matrix["0", "0"]

# --- Metrics ---

sensitivity <- true_positive / (true_positive + false_negative)

specificity <- true_negative / (true_negative + false_positive)

accuracy <- (true_positive + true_negative) / sum(conf_matrix)

cat("\n--- Metrics at Predefined Cutoff (0.20) ---\n")

cat("Sensitivity:", round(sensitivity, 4), "\n")

cat("Specificity:", round(specificity, 4), "\n")

cat("Accuracy:", round(accuracy, 4), "\n")

# --- Bootstrap 95% CI for Sensitivity, Specificity, Accuracy ---

set.seed(123)

nboot <- 2000

boot_results <- replicate(nboot, {

idx <- sample(seq_along(actual_outcome), replace = TRUE)

pred <- predicted_outcome[idx]

act <- actual_outcome[idx]

cm <- table(Pred = pred, Act = act)

tp <- cm["1", "1"]; fn <- cm["0", "1"]

fp <- cm["1", "0"]; tn <- cm["0", "0"]

sens <- ifelse((tp + fn) > 0, tp / (tp + fn), NA)

spec <- ifelse((tn + fp) > 0, tn / (tn + fp), NA)

acc <- (tp + tn) / sum(cm)

c(sens, spec, acc)

})

sens_ci <- quantile(boot_results[1, ], c(0.025, 0.975), na.rm = TRUE)

spec_ci <- quantile(boot_results[2, ], c(0.025, 0.975), na.rm = TRUE)

acc_ci <- quantile(boot_results[3, ], c(0.025, 0.975), na.rm = TRUE)

cat("\n--- 95% Confidence Intervals (Bootstrap, 2000 resamples) ---\n")

cat("Sensitivity CI:", paste(round(sens_ci, 4), collapse = " - "), "\n")

cat("Specificity CI:", paste(round(spec_ci, 4), collapse = " - "), "\n")

cat("Accuracy CI:", paste(round(acc_ci, 4), collapse = " - "), "\n")

#####

### LB cutoff = 0.20

# --- Define a Predefined Cutoff Point ---

# Let's say, based on previous research or clinical guidelines,

library(pROC)

# a predictor value above 0.20 is considered positive.

cutoff_point <- 0.20

# --- Calculate Sensitivity and Specificity ---

# Convert predictor to binary based on the cutoff point

predicted_outcome <- as.factor(ifelse(rocdata$LB.admission >= cutoff_point, 1, 0))

# Create a confusion matrix

conf_matrix <- table(Predicted = predicted_outcome, Actual = rocdata$critical_dengue) ## Predicted vs outcome data

print("Confusion Matrix:")

print(conf_matrix)

# Extract values from confusion matrix

# Assuming '1' is the positive class and '0' is the negative class

true_positive <- conf_matrix["1", "1"]

false_negative <- conf_matrix["0", "1"]

false_positive <- conf_matrix["1", "0"]

true_negative <- conf_matrix["0", "0"]

# Calculate Sensitivity (True Positive Rate)

# Sensitivity = TP / (TP + FN)

sensitivity <- true_positive / (true_positive + false_negative)

# Calculate Specificity (True Negative Rate)

# Specificity = TN / (TN + FP)

specificity <- true_negative / (true_negative + false_positive)

cat("\n--- Metrics at Predefined Cutoff ---\n")

cat("Cutoff Point:", cutoff_point, "\n")

cat("Sensitivity:", round(sensitivity, 4), "\n")

cat("Specificity:", round(specificity, 4), "\n")

# Calculate Accuracy

accuracy <- (true_positive + true_negative) /

(true_positive + true_negative + false_positive + false_negative)

cat("Accuracy:", round(accuracy, 4), "\n")

# --- Calculate AUC (Area Under the ROC Curve) ---

# AUC does not depend on a specific cutoff point; it evaluates the overall discriminatory ability

### Additional analysis for LB cutoff > = 0.2

library(pROC)

library(epiR)

# --- Define a Predefined Cutoff Point ---

cutoff_point <- 0.2

# --- Calculate Predicted Outcome ---

predicted_outcome <- as.factor(ifelse(rocdata$LB.admission >= cutoff_point, 1, 0))

# Ensure actual outcome is factor with 0/1 levels

rocdata$critical_dengue <- factor(rocdata$critical_dengue, levels = c(0,1))

# --- Confusion Matrix ---

conf_matrix <- table(Predicted = predicted_outcome, Actual = rocdata$critical_dengue)

print("Confusion Matrix:")

print(conf_matrix)

# --- Use epiR to calculate metrics with 95% CI ---

# epi.tests expects a matrix with columns = actual (disease status), rows = test result

# Format: [Test Positive, Test Negative] x [Disease Positive, Disease Negative]

epi_res <- epi.tests(conf_matrix)

print(epi_res)

1. **Univariable analyses of associations between the composite endpoints and biomarkers and other predetermined predictors**

**S3 Table. The unadjusted associations between the covariables and composite endpoint**

# R code

# Univariate Logistic Regression-Composite endpoint and LAR,LB and Lactate:

# 3.1 Logistic Regression-LAR

model1 <- glm(critical_dengue ~ LA.admission,

family = binomial(link = "logit"),

data = df)

# Model summary

summary(model1)

# Odds ratios with 95% CI

library(epiDisplay)

logistic.display(model1)

### 3.2 Logistic Regression-LB ratio

model2 <- glm(critical_dengue ~ LB.admission,

family = binomial(link = "logit"),

data = df)

# Model summary

summary(model2)

# Odds ratios with 95% CI

library(epiDisplay)

logistic.display(model2)

### 3.3 Logistic Regression-Lactate.admission

model3 <- glm(critical_dengue ~ Lactate.admission,

family = binomial(link = "logit"),

data = df)

# Model summary

summary(model3)

# Odds ratios with 95% CI

library(epiDisplay)

logistic.display(model3)

### 3.4 Age

model4 <- glm(critical_dengue ~ age.year,

family = binomial(link = "logit"),

data = df)

# Model summary

summary(model4)

# Odds ratios with 95% CI

library(epiDisplay)

logistic.display(model4)

### 3.5 Female sex

model5 <- glm(critical_dengue ~ sex.female,

family = binomial(link = "logit"),

data = df)

# Model summary

summary(model5)

# Odds ratios with 95% CI

library(epiDisplay)

logistic.display(model5)

### 3.6 underlying.disease

model6 <- glm(critical_dengue ~ underlying.disease,

family = binomial(link = "logit"),

data = df)

# Model summary

summary(model6)

# Odds ratios with 95% CI

library(epiDisplay)

logistic.display(model6)

### 3.7 Early onset.day.shock

model7 <- glm(critical_dengue ~ onset.day.shock,

family = binomial(link = "logit"),

data = df)

# Model summary

summary(model7)

# Odds ratios with 95% CI

library(epiDisplay)

logistic.display(model7)

### 3.8 DSS.Decompensated

model8 <- glm(critical_dengue ~ DSS.Decompensated,

family = binomial(link = "logit"),

data = df)

# Model summary

summary(model8)

# Odds ratios with 95% CI

library(epiDisplay)

logistic.display(model8)

### 3.9 Respiratory rate

model9 <- glm(critical_dengue ~ Res.rate,

family = binomial(link = "logit"),

data = df)

# Model summary

summary(model9)

# Odds ratios with 95% CI

library(epiDisplay)

logistic.display(model9)

### 3.10 Systolic.shock.index

model10 <- glm(critical_dengue ~ Sys.shock.index,

family = binomial(link = "logit"),

data = df)

# Model summary

summary(model10)

# Odds ratios with 95% CI

library(epiDisplay)

logistic.display(model10)

### 3.11 AVPU.admission

model11 <- glm(critical_dengue ~ AVPU.admission,

family = binomial(link = "logit"),

data = df)

# Model summary

summary(model11)

# Odds ratios with 95% CI

library(epiDisplay)

logistic.display(model11)

### 3.12 Severe.bleeding

model12 <- glm(critical_dengue ~ Severe.bleeding,

family = binomial(link = "logit"),

data = df)

# Model summary

summary(model12)

# Odds ratios with 95% CI

library(epiDisplay)

logistic.display(model12)

### 3.13 Severe.hepatitis

model13 <- glm(critical_dengue ~ Severe.hepatitis,

family = binomial(link = "logit"),

data = df)

# Model summary

summary(model13)

# Odds ratios with 95% CI

library(epiDisplay)

logistic.display(model13)

### 3.14 HCT.peak

model14 <- glm(critical_dengue ~ HCT.peak,

family = binomial(link = "logit"),

data = df)

# Model summary

summary(model14)

# Odds ratios with 95% CI

library(epiDisplay)

logistic.display(model14)

### 3.15 HCT.nadir

model15 <- glm(critical_dengue ~ HCT.nadir,

family = binomial(link = "logit"),

data = df)

# Model summary

summary(model15)

# Odds ratios with 95% CI

library(epiDisplay)

logistic.display(model15)

### 3.16 Creatinin

model16 <- glm(critical_dengue ~ Creatinin ,

family = binomial(link = "logit"),

data = df)

# Model summary

summary(model16)

# Odds ratios with 95% CI

library(epiDisplay)

logistic.display(model16)

### 3.17 Serum albumin

model17 <- glm(critical_dengue ~ Albumin.Admission,

family = binomial(link = "logit"),

data = df)

# Model summary

summary(model17)

# Odds ratios with 95% CI

library(epiDisplay)

logistic.display(model17)

### 3.18 Serum Bicarbonate

model18 <- glm(critical_dengue ~ HCO3.admission,

family = binomial(link = "logit"),

data = df)

# Model summary

summary(model18)

# Odds ratios with 95% CI

library(epiDisplay)

logistic.display(model18)

### 3.19 Low platelet count (<20 x 109/L)

model19 <- glm(critical_dengue ~ PLT.lower20K,

family = binomial(link = "logit"),

data = df)

# Model summary

summary(model19)

# Odds ratios with 95% CI

library(epiDisplay)

logistic.display(model19)

### 3.20 Requiring platetet transfusion

model20 <- glm(critical_dengue ~ PLT.transfusion,

family = binomial(link = "logit"),

data = df)

# Model summary

summary(model20)

# Odds ratios with 95% CI

library(epiDisplay)

logistic.display(model20)

### 3.21 International normalized ratio (INR)

model21 <- glm(critical_dengue ~ INR,

family = binomial(link = "logit"),

data = df)

# Model summary

summary(model21)

# Odds ratios with 95% CI

library(epiDisplay)

logistic.display(model21)

### 3.22 High vasoactive inotropic score (> 30)

model22 <- glm(critical_dengue ~ VIS.24h.over30,

family = binomial(link = "logit"),

data = df)

# Model summary

summary(model22)

# Odds ratios with 95% CI

library(epiDisplay)

logistic.display(model22)

### 3.23 Cumulative amount of fluid infused from referral hospitals and 24h admission (Log2)

model23 <- glm(critical_dengue ~ Cumulative.fluid.24h,

family = binomial(link = "logit"),

data = df)

# Model summary

summary(model23)

# Odds ratios with 95% CI

library(epiDisplay)

logistic.display(model23)

### Log-2 transformation of VIS 24h

df$log2VIS<-log2(df$VIS.24h+1)

### 3.24 Cumulative amount of fluid infused from referral hospitals and 24h admission (Log2)

model24 <- glm(critical_dengue ~ log2VIS,

family = binomial(link = "logit"),

data = df)

# Model summary

summary(model24)

# Odds ratios with 95% CI

library(epiDisplay)

logistic.display(model24)

###

1. **Lasso regression**

## Complete-case data analysis

rm(list=ls()) #### Clear all working commands

# --- Load required packages ---

library(readxl)

# --- Import Excel file ---

S1_Appendix <- read_excel(

"C:/Users/HP/Desktop/S1_Appendix.xlsx",

sheet = "S1_Appendix_Dataset"

)

# --- Quick check of the first rows ---

head(S1_Appendix, 3)

names(S1_Appendix)

# Select all predefined variables into df

library(dplyr)

df <- S1_Appendix %>%

select(

age.year, sex,underlying.disease, onset.day.shock, DSS.Decompensated, Severe.bleeding,

AVPU.Pain.Unresponse, Res.rate, Sys.shock.index,

HCT.peak, HCT.nadir, PLT.lower20K, PLT.transfusion, INR, Creatinin, Severe.hepatitis,

Albumin.Admission.g.dL, Lactate.admission, HCO3.admission, LA.admission, LB.admission,

Cumulative.fluid.24h, VIS.24h.over30, critical_dengue

)

# Load the required libraries

library(glmnet)

library(caret)

#### LASSO regression

# Convert outcome to numeric if it's still in character form as 0/1 format for target var

df$critical_dengue <- as.numeric(df$critical_dengue)

# Remove rows with any missing values to ensure consistency ## Lasso only accept non-missing dataset

df_clean <- na.omit(df) ### No missing values are important condition to perform LASSO regression

# Define predictor matrix and response vector

x <- model.matrix(critical_dengue ~ . - 1, data = df_clean) # Remove intercept

y <- df_clean$critical_dengue

# Set seed for reproducibility

set.seed(123)

# Perform cross-validated LASSO (alpha = 1 for LASSO)

cv.lasso <- cv.glmnet(x, y, family = "binomial", alpha = 1)

coef(cv.lasso, s = "lambda.min")

# Plot cross-validation results

plot(cv.lasso)

# Optional: Extract best lambda

best_lambda <- cv.lasso$lambda.min

print(paste("Best lambda:", best_lambda))

# Fit the LASSO model without cross-validation to get the full coefficient path

lasso_fit <- glmnet(x, y, family = "binomial", alpha = 1)

# Plot the Lasso coefficient paths

plot(lasso_fit, xvar = "lambda", label = TRUE)

title("Coefficient Path Plot for LASSO")

# Extract the coefficients at the optimal lambda

lasso_coefs <- coef(cv.lasso, s = "lambda.min")

# Convert the coefficients to a data frame for better readability

selected_variables <- as.data.frame(as.matrix(lasso_coefs))

selected_variables <- selected_variables[selected_variables != 0, , drop = FALSE]

selected_variables <- data.frame(Variable = rownames(selected_variables), Coefficient = selected_variables[, 1])

# Print the selected variables

print(selected_variables)

####

1. **Data preprocessing**

**Multiple imputation analysis**

# R code

rm(list=ls()) ### Clear the working environments

### Multiple imputation dataset with predictive mean matching (pmm) method

# --- Load required packages ---

library(readxl)

# --- Import Excel file ---

S1_Appendix <- read_excel(

"C:/Users/HP/Desktop/S1_Appendix.xlsx",

sheet = "S1_Appendix_Dataset"

)

# --- Quick check of the first rows ---

head(S1_Appendix, 3)

names(S1_Appendix)

# Select all predefined variables into df

library(dplyr)

df <- S1_Appendix %>%

select(critical_dengue,

age.year, sex,underlying.disease, onset.day.shock, DSS.Decompensated, Severe.bleeding,

AVPU.Pain.Unresponse, Res.rate, Sys.shock.index,

HCT.peak, HCT.nadir, PLT.lower20K, PLT.transfusion, INR, Creatinin, Severe.hepatitis,

Albumin.Admission.g.dL, Lactate.admission, HCO3.admission, LA.admission, LB.admission,

Cumulative.fluid.24h, VIS.24h.over30

)

### Data Exploration

# Exploring and understanding data

head(df)

tail(df)

dim(df)

library(tidyverse)

glimpse(df)

str(df)

# Check missing

library(rms)

na.patterns = naclus(df)

plot(na.patterns)

library(naniar)

gg_miss_var(df)

library(VIM)

res<-summary(aggr(df, sortVar=TRUE))$combinations

vis_miss(df, sort_miss = TRUE) # It also provides the percentage of missing values in each column.

library(dlookr)

plot_na_pareto(df) # It will show pareto chart with missing values

plot_na_hclust(df) # Distribution of missing value by combination of variables

plot_na_intersect(df) # Missing with intersection of variables

# Calculate the percentage of missing values for each variable

missing_percentage <- sapply(df, function(x) sum(is.na(x)) / length(x)) * 100

# Filter the variables with more than 20% missing values

variables_above_20_percent_missing <- names(missing_percentage[missing_percentage > 20])

# Count how many variables have more than 20% missing values

num_variables_above_20_percent_missing <- length(variables_above_20_percent_missing)

# Print the result

print(num_variables_above_20_percent_missing)

print(variables_above_20_percent_missing)

# Select the variables with less than 20% missing values

variables_below_20_percent_missing <- names(missing_percentage[missing_percentage < 20])

# Create the new data set 'df1' with only the selected variables < 20% missingness

df1 <- df %>% select(all_of(variables_below_20_percent_missing))

head(df1)

plot_na_pareto(df1)

variables_below_20_percent_missing

################ Missing imputation process #########################

### Create imputation dataset of all predictors

# Drop outcome

df2 <- subset(df1, select = -c(critical_dengue)) ## outcome variable

library(missRanger)

# Perform imputation with predictive mean matching (pmm) in missRanger package:

impu <- missRanger(df2,

formula = .~.,

num.trees = 1000,

seed = 3)

# Add the outcome variable back to the multiple imputed dataset

LALB.multiple.imputation <- cbind(impu, outcome = df1$critical_dengue) ####### new variable "outcome" = "critical_dengue"

# Check for missing data in each column for sure

summary(LALB.multiple.imputation)

str(LALB.multiple.imputation)

missing_data <- colSums(is.na(LALB.multiple.imputation))

missing_data

### Export multiple imputation dataset into excel file

write.csv(LALB.multiple.imputation, file = "C:/Users/HP/Desktop/LALB.multiple.imputation.CSV")

#### Multiple imputation is completed #######

#### Import the imputation dataset ######

LALB.multiple.imputation <- read.csv("C:/Users/HP/Desktop/LALB.multiple.imputation.CSV")

#### This dataset (LALB.multiple.imputation) is ready for supervised machine learning modelling in the next step

1. **Performance of supervised models**

**Table 3**- Performance of supervised models to estimate the risk of the critical clinical dengue outcomes children admitted with dengue shock syndrome

# R code

**# 6-1 LR, Logistic regression**

## --- Load packages ---

# install.packages(c("caret","pROC","binom"))

library(caret)

library(pROC)

library(binom)

## --- Import dataset ---

mydata <- read.csv("C:/Users/HP/Desktop/LALB.multiple.imputation.CSV")

## Factorize outcome

mydata$critical_dengue <- factor(mydata$critical_dengue,

levels = c(0,1),

labels = c("No","Yes"))

## --- Train/test split ---

set.seed(123)

trainIndex <- createDataPartition(mydata$critical_dengue, p = 0.7, list = FALSE)

train <- mydata[trainIndex, ]

test <- mydata[-trainIndex, ]

## --- Train Logistic Regression model ---

log_model <- glm(critical_dengue ~ ., data = train, family = binomial)

## --- Predictions ---

log_prob <- predict(log_model, newdata = test, type = "response")

log_pred <- factor(ifelse(log_prob > 0.5, "Yes","No"), levels = c("No","Yes"))

## --- Confusion Matrix ---

cm <- confusionMatrix(log_pred, test$critical_dengue, positive = "Yes")

print(cm)

## --- Extract metrics ---

TP <- cm$table[2,2]

TN <- cm$table[1,1]

FP <- cm$table[1,2]

FN <- cm$table[2,1]

sens <- TP / (TP + FN)

spec <- TN / (TN + FP)

prec <- TP / (TP + FP)

acc <- (TP + TN) / (TP + TN + FP + FN)

f1 <- 2 * (prec * sens) / (prec + sens)

metrics <- data.frame(

Metric = c("Accuracy","Sensitivity","Specificity","Precision","F1 Score"),

Estimate = c(acc, sens, spec, prec, f1)

)

print(metrics)

## --- 95% CI for metrics ---

acc_ci <- binom.confint(TP+TN, TP+TN+FP+FN, method="wilson")

sens_ci <- binom.confint(TP, TP+FN, method="wilson")

spec_ci <- binom.confint(TN, TN+FP, method="wilson")

prec_ci <- binom.confint(TP, TP+FP, method="wilson")

# Bootstrap CI for F1

set.seed(123)

boot_f1 <- replicate(1000, {

idx <- sample(1:nrow(test), replace=TRUE)

y_true <- test$critical_dengue[idx]

y_prob <- log_prob[idx]

y_pred <- ifelse(y_prob > 0.5, "Yes","No")

y_pred <- factor(y_pred, levels = c("No","Yes"))

cm_boot <- table(y_true, y_pred)

TPb <- cm_boot["Yes","Yes"]

FPb <- cm_boot["No","Yes"]

FNb <- cm_boot["Yes","No"]

prec_b <- TPb / (TPb + FPb)

sens_b <- TPb / (TPb + FNb)

if(is.nan(prec_b) | is.nan(sens_b)) return(NA)

2*(prec_b*sens_b)/(prec_b+sens_b)

})

boot_f1 <- na.omit(boot_f1)

f1_ci <- quantile(boot_f1, c(0.025, 0.975))

## --- ROC with AUC + 95% CI ---

roc_log <- roc(test$critical_dengue, log_prob)

auc(roc_log)

ci.auc(roc_log)

plot(roc_log, col="blue", lwd=2, main="ROC Curve - Logistic Regression")

## --- Final Summary Table ---

summary_table <- data.frame(

Metric = c("AUC","Accuracy","Sensitivity","Specificity","Precision","F1 Score"),

Estimate = c(auc(roc_log), acc, sens, spec, prec, f1),

CI_Lower = c(ci.auc(roc_log)[1], acc_ci$lower, sens_ci$lower, spec_ci$lower, prec_ci$lower, f1_ci[1]),

CI_Upper = c(ci.auc(roc_log)[3], acc_ci$upper, sens_ci$upper, spec_ci$upper, prec_ci$upper, f1_ci[2])

)

print(summary_table)

###

**# 6-2 RF, Random Forest**

rm(list=ls()) #### Clear all working commands

### Supervised models

## Import dataset:

mydata <- read.csv("C:/Users/HP/Desktop/LALB.multiple.imputation.CSV")

head(mydata,3)

#### Factor outcome var

mydata$critical_dengue <- as.factor(mydata$critical_dengue)

### Label and factor study OUTCOMES

mydata$critical_dengue = factor(mydata$critical_dengue,

levels = c(0, 1),

labels = c("No", "Yes"))

dim(mydata)

### Factor all categorical variables:

mydata$sex.female <- as.factor(mydata$sex.female)

mydata$DSS.Decompensated <- as.factor(mydata$DSS.Decompensated)

mydata$underlying.disease <- as.factor(mydata$underlying.disease)

mydata$onset.day.shock <- as.factor(mydata$onset.day.shock)

mydata$Severe.bleeding <- as.factor(mydata$Severe.bleeding)

mydata$PLT.lower20K <- as.factor(mydata$PLT.lower20K)

mydata$PLT.transfusion <- as.factor(mydata$PLT.transfusion)

mydata$AVPU.admission <- as.factor(mydata$AVPU.admission)

mydata$Severe.hepatitis <- as.factor(mydata$Severe.hepatitis)

mydata$VIS.24h.over30 <- as.factor(mydata$VIS.24h.over30)

## Recheck missingness:

missing_data <- colSums(is.na(mydata))

missing_data

## No missing data is present in the dataset analyzed

## First look at dataset:

library(dplyr)

glimpse(mydata)

dim(mydata) #### 524 (subjects) 24 (variables)

## --- Libraries ---

library(randomForest)

library(caret)

library(pROC)

library(binom)

library(epiR) # for sensitivity/specificity CI

library(dplyr)

library(caTools) # split function

set.seed(120)

## --- Train/test split ---

split <- sample.split(mydata$critical_dengue, SplitRatio = 0.7)

train <- subset(mydata, split == TRUE)

test <- subset(mydata, split == FALSE)

## --- Fit Random Forest ---

rf_model <- randomForest(critical_dengue ~ .,

data = train,

ntree = 500, importance = TRUE)

## --- Predictions ---

rf_probs <- predict(rf_model, test, type = "prob")[,2]

rf_pred <- predict(rf_model, test)

## --- Confusion Matrix ---

cm <- confusionMatrix(rf_pred, test$critical_dengue, positive = "Yes")

cm

## Extract counts for CI calc

tp <- cm$table[2,2]; fn <- cm$table[1,2]

tn <- cm$table[1,1]; fp <- cm$table[2,1]

n <- sum(cm$table)

## --- Accuracy + CI ---

acc <- cm$overall["Accuracy"]

acc_ci <- binom.confint(tp+tn, n, conf.level=0.95, methods="wilson")

## --- Sensitivity + CI ---

sens <- cm$byClass["Sensitivity"]

sens_ci <- binom.confint(tp, tp+fn, conf.level=0.95, methods="wilson")

## --- Specificity + CI ---

spec <- cm$byClass["Specificity"]

spec_ci <- binom.confint(tn, tn+fp, conf.level=0.95, methods="wilson")

## --- Precision (PPV) + CI ---

ppv <- cm$byClass["Pos Pred Value"]

ppv_ci <- binom.confint(tp, tp+fp, conf.level=0.95, methods="wilson")

## --- F1 Score (point estimate) ---

f1 <- 2 * (ppv * sens) / (ppv + sens)

# ### 95% CI for AUC and other metrics for Random Forest model:

# Bootstrap 95% CI for F1 score

boot_f1 <- replicate(1000, {

idx <- sample(seq_len(n), replace = TRUE)

cm_boot <- confusionMatrix(rf_pred[idx], test$critical_dengue[idx], positive = "Yes")

tp_b <- cm_boot$table[2,2]; fn_b <- cm_boot$table[1,2]

tn_b <- cm_boot$table[1,1]; fp_b <- cm_boot$table[2,1]

sens_b <- tp_b / (tp_b + fn_b)

ppv_b <- tp_b / (tp_b + fp_b)

2 * (ppv_b * sens_b) / (ppv_b + sens_b)

})

f1_ci <- quantile(boot_f1, c(0.025, 0.975))

## --- AUC + 95% CI ---

roc_obj <- roc(test$critical_dengue, rf_probs)

auc_val <- auc(roc_obj)

auc_ci <- ci.auc(roc_obj, conf.level=0.95)

## --- Summary Table ---

results <- data.frame(

Metric = c("Accuracy", "Sensitivity", "Specificity", "Precision (PPV)", "F1 Score", "AUC"),

Estimate = c(acc, sens, spec, ppv, f1, auc_val),

CI_Lower = c(acc_ci$lower, sens_ci$lower, spec_ci$lower, ppv_ci$lower, f1_ci[1], auc_ci[1]),

CI_Upper = c(acc_ci$upper, sens_ci$upper, spec_ci$upper, ppv_ci$upper, f1_ci[2], auc_ci[3])

)

print(results, row.names = FALSE)

####

### Hypertunining Random Forest:

### We can tune a number of trees and mtry basis below the function.

t <- tuneRF(train[,-23], train[,23],

stepFactor = 0.5,

plot = TRUE,

ntreeTry = 150,

trace = TRUE,

improve = 0.05)

## Then we have, mtry = 14 has lowest error rate = 2.7%

## Input back the RF model tuned

t <- tuneRF(train[,-23], train[,23],

stepFactor = 0.5,

plot = TRUE,

ntreeTry = 14,

trace = TRUE,

improve = 0.05)

####No. of nodes for the trees

hist(treesize(RF),

main = "No. of Nodes for the Trees",

col = "green")

### Tunining Random Forest Model:

# Multi-dimensional Scaling Plot of Proximity Matrix

# Dimension plot also can create from random forest model.

# MDSplot(RF, train$critical_dengue)

# dataframe from importance() output => "RF" is the name of model

**Figure 2**- Comparisons of serum lactate levels, lactate-derived ratios, and significant clinical variables of the Random Forest model based on classification accuracy.

#### Variables of importance in Random Forest (GINI and Accuracy)

library(tidyverse)

library(ggplot2)

feat_imp_df <- importance(RF) %>%

data.frame() %>%

mutate(feature = row.names(.))

### Plot the variables of most importance from RF model

ggplot(feat_imp_df, aes(x = reorder(feature, MeanDecreaseGini),

y = MeanDecreaseGini)) +

geom_bar(stat='identity') +

coord_flip() +

theme_classic() +

labs(

x = "Features",

y = "Mean decrease in accuracy",

title = "Random Forest-Features of Importance for composite endpoint"

)

# **Fig 2** in the submitted manuscript

#### AUC and 95%CI AUC for Random Forest model:

# load packages

library(pROC)

library(dplyr)

library(randomForest)

forest=randomForest(critical_dengue~.,data=train,importance=T,ntree=2000)

#### Prediction

test_pred <- predict(forest, test, type = "prob")[,2]

#### AUC Random forest

library(pROC)

roc_test_RF <- roc(test$critical_dengue, test_pred, algorithm = 2) #### test_y = outcome (only) matrix in test dataset

plot(roc_test_RF)

# Calculate area under ROC curve

auc(roc_test_RF)

#### 95% CI AUC from Random forest

ci.auc(test$critical_dengue, test_pred) #### outcome.MV = Yes(1)/ No(0)

**# 6-3 XGBoost model**

## --- Load packages ---

# install.packages(c("xgboost", "caret", "pROC", "binom"))

library(xgboost)

library(caret)

library(pROC)

library(binom)

## --- Import dataset ---

mydata <- read.csv("C:/Users/HP/Desktop/LALB.multiple.imputation.CSV")

## Factorize outcome

mydata$critical_dengue <- factor(mydata$critical_dengue,

levels = c(0,1),

labels = c("No","Yes"))

## --- Train/test split ---

set.seed(123)

trainIndex <- createDataPartition(mydata$critical_dengue, p = 0.7, list = FALSE)

train <- mydata[trainIndex, ]

test <- mydata[-trainIndex, ]

## --- Convert to matrix for XGBoost ---

train_matrix <- xgb.DMatrix(data = as.matrix(train[, -24]),

label = as.numeric(train$critical_dengue) - 1)

test_matrix <- xgb.DMatrix(data = as.matrix(test[, -24]),

label = as.numeric(test$critical_dengue) - 1)

## --- Train XGBoost model ---

set.seed(123)

xgb_model <- xgboost(

data = train_matrix,

booster = "gbtree",

objective = "binary:logistic",

eval_metric = "auc",

nrounds = 200,

max_depth = 4,

eta = 0.1,

subsample = 0.8,

colsample_bytree = 0.8,

verbose = 0

)

## --- Predictions on test set ---

xgb_pred_prob <- predict(xgb_model, newdata = test_matrix)

xgb_pred_class <- ifelse(xgb_pred_prob > 0.5, "Yes", "No")

xgb_pred_class <- factor(xgb_pred_class, levels = c("No","Yes"))

## --- Confusion matrix ---

cm <- confusionMatrix(xgb_pred_class, test$critical_dengue, positive = "Yes")

print(cm)

## --- Extract metrics ---

TP <- cm$table[2,2]

TN <- cm$table[1,1]

FP <- cm$table[1,2]

FN <- cm$table[2,1]

sens <- TP / (TP + FN)

spec <- TN / (TN + FP)

prec <- TP / (TP + FP)

acc <- (TP + TN) / (TP + TN + FP + FN)

f1 <- 2 * (prec * sens) / (prec + sens)

metrics <- data.frame(

Metric = c("Accuracy","Sensitivity","Specificity","Precision","F1 Score"),

Estimate = c(acc, sens, spec, prec, f1)

)

print(metrics)

## --- 95% CI for metrics ---

acc_ci <- binom.confint(TP+TN, TP+TN+FP+FN, method="wilson")

sens_ci <- binom.confint(TP, TP+FN, method="wilson")

spec_ci <- binom.confint(TN, TN+FP, method="wilson")

prec_ci <- binom.confint(TP, TP+FP, method="wilson")

# Bootstrap CI for F1

set.seed(123)

boot_f1 <- replicate(1000, {

idx <- sample(1:nrow(test), replace=TRUE)

y_true <- test$critical_dengue[idx]

y_prob <- xgb_pred_prob[idx]

y_pred <- ifelse(y_prob > 0.5, "Yes","No")

y_pred <- factor(y_pred, levels = c("No","Yes"))

cm_boot <- table(y_true, y_pred)

TPb <- cm_boot["Yes","Yes"]

FPb <- cm_boot["No","Yes"]

FNb <- cm_boot["Yes","No"]

prec_b <- TPb / (TPb + FPb)

sens_b <- TPb / (TPb + FNb)

if(is.nan(prec_b) | is.nan(sens_b)) return(NA)

2*(prec_b*sens_b)/(prec_b+sens_b)

})

boot_f1 <- na.omit(boot_f1)

f1_ci <- quantile(boot_f1, c(0.025, 0.975))

## --- AUC with 95% CI ---

roc_xgb <- roc(test$critical_dengue, xgb_pred_prob)

auc(roc_xgb)

ci.auc(roc_xgb)

plot(roc_xgb, col="red", lwd=2, main="ROC Curve - XGBoost")

## --- Final summary table ---

summary_table <- data.frame(

Metric = c("AUC","Accuracy","Sensitivity","Specificity","Precision","F1 Score"),

Estimate = c(auc(roc_xgb), acc, sens, spec, prec, f1),

CI_Lower = c(ci.auc(roc_xgb)[1], acc_ci$lower, sens_ci$lower, spec_ci$lower, prec_ci$lower, f1_ci[1]),

CI_Upper = c(ci.auc(roc_xgb)[3], acc_ci$upper, sens_ci$upper, spec_ci$upper, prec_ci$upper, f1_ci[2])

)

print(summary_table)

###

**## 6-4 AdaBoost model**

# --- Packages ---

library(caret)

library(adabag)

library(pROC)

library(binom)

set.seed(123)

# --- Train/test split ---

idx <- createDataPartition(mydata$critical_dengue, p = 0.7, list = FALSE)

train <- mydata[idx, ]

test <- mydata[-idx, ]

# --- Train AdaBoost ---

ada_model <- boosting(

critical_dengue ~ .,

data = train,

boos = TRUE,

mfinal = 100

)

# --- Predictions ---

ada_pred <- predict(ada_model, newdata = test)

# Ensure probability matrix has proper column names

colnames(ada_pred$prob) <- levels(train$critical_dengue)

# --- Confusion Matrix ---

cm <- confusionMatrix(

factor(ada_pred$class, levels = c("No","Yes")),

test$critical_dengue,

positive = "Yes"

)

print(cm)

# --- AUC with 95% CI ---

roc_obj <- roc(test$critical_dengue, ada_pred$prob[, "Yes"], levels = c("No","Yes"))

auc_val <- auc(roc_obj)

auc_ci <- ci.auc(roc_obj)

print(auc_val)

print(auc_ci)

# --- Other metrics with 95% CI ---

TP <- cm$table["Yes","Yes"]

FP <- cm$table["No","Yes"]

TN <- cm$table["No","No"]

FN <- cm$table["Yes","No"]

accuracy <- (TP + TN) / sum(cm$table)

sensitivity <- TP / (TP + FN)

specificity <- TN / (TN + FP)

precision <- TP / (TP + FP)

f1_score <- 2 * (precision * sensitivity) / (precision + sensitivity)

# Binomial CIs for Accuracy, Sensitivity, Specificity, Precision

acc_ci <- binom.confint(TP+TN, sum(cm$table), methods = "wilson")[c("lower","upper")]

sens_ci <- binom.confint(TP, TP+FN, methods = "wilson")[c("lower","upper")]

spec_ci <- binom.confint(TN, TN+FP, methods = "wilson")[c("lower","upper")]

prec_ci <- binom.confint(TP, TP+FP, methods = "wilson")[c("lower","upper")]

# --- Bootstrap F1 95% CI ---

set.seed(123)

boot_f1 <- replicate(1000, {

idx <- sample(1:nrow(test), replace=TRUE)

y_true <- test$critical_dengue[idx]

y_prob <- ada_pred$prob[idx, "Yes"]

y_pred <- ifelse(y_prob > 0.5, "Yes", "No")

y_pred <- factor(y_pred, levels = c("No","Yes"))

cm_boot <- table(y_true, y_pred)

TPb <- ifelse("Yes" %in% rownames(cm_boot) && "Yes" %in% colnames(cm_boot), cm_boot["Yes","Yes"], 0)

FPb <- ifelse("No" %in% rownames(cm_boot) && "Yes" %in% colnames(cm_boot), cm_boot["No","Yes"], 0)

FNb <- ifelse("Yes" %in% rownames(cm_boot) && "No" %in% colnames(cm_boot), cm_boot["Yes","No"], 0)

prec_b <- ifelse((TPb + FPb) > 0, TPb / (TPb + FPb), NA)

sens_b <- ifelse((TPb + FNb) > 0, TPb / (TPb + FNb), NA)

if(is.na(prec_b) | is.na(sens_b)) return(NA)

2 * (prec_b * sens_b) / (prec_b + sens_b)

})

boot_f1 <- na.omit(boot_f1)

f1_ci <- quantile(boot_f1, c(0.025, 0.975))

# --- Summary Table ---

results <- data.frame(

Metric = c("Accuracy", "Sensitivity", "Specificity", "Precision", "F1 Score", "AUC"),

Estimate = c(accuracy, sensitivity, specificity, precision, f1_score, auc_val),

CI_lower = c(acc_ci$lower, sens_ci$lower, spec_ci$lower, prec_ci$lower, f1_ci[1], auc_ci[1]),

CI_upper = c(acc_ci$upper, sens_ci$upper, spec_ci$upper, prec_ci$upper, f1_ci[2], auc_ci[3])

)

print(results)

###

**## 6-5 SVM, Support vector machine**

# --- Libraries ---

library(readxl)

library(dplyr)

library(caret)

library(e1071)

# --- Import Excel file ---

df <- read.csv("C:/Users/HP/Desktop/LALB.multiple.imputation.CSV")

head(df,3)

# --- Make sure outcome is a factor (caret needs factor for classification) ---

df$critical_dengue <- factor(df$critical_dengue, levels = c(0,1), labels = c("No","Yes"))

# --- Data partition (70/30 split) ---

set.seed(123)

index <- createDataPartition(df$critical_dengue, p = 0.7, list = FALSE)

train <- df[index, ] # train set

test <- df[-index, ] # test set

# --- Normalization (center + scale all predictors) ---

scale <- preProcess(train[, 1:23], method = c("center", "scale"))

train_scaled <- predict(scale, train)

test_scaled <- predict(scale, test)

library(caret)

library(e1071) ### SVM

library(tidyverse)

# --- Cross-validation ---

ctr <- trainControl(

method = "cv",

number = 5,

classProbs = TRUE,

summaryFunction = twoClassSummary

)

# --- Train SVM (Radial kernel) ---

svm_model <- caret::train(

critical_dengue ~ .,

data = train_scaled,

method = "svmRadial",

trControl = ctr,

metric = "ROC"

)

# --- Predictions ---

pred_train <- predict(svm_model, train_scaled)

pred_test <- predict(svm_model, test_scaled)

# --- Show results ---

confusionMatrix(pred_train, train_scaled$critical_dengue)

confusionMatrix(pred_test, test_scaled$critical_dengue)

library(pROC)

# --- Predict class probabilities (needed for ROC curve) ---

pred_prob <- predict(svm_model, test_scaled, type = "prob")

# --- Build ROC curve (assumes "Yes" is the positive class) ---

roc_obj <- roc(

response = test_scaled$critical_dengue,

predictor = pred_prob$Yes, # column name = "Yes"

levels = c("No", "Yes"), # set the order of classes

direction = "<"

)

# --- Print AUC ---

auc(roc_obj)

# --- Plot ROC curve ---

plot(

roc_obj,

col = "blue",

lwd = 2,

main = "ROC Curve for SVM on Test Set"

)

abline(a = 0, b = 1, lty = 2, col = "red") # diagonal line

###

library(pROC)

# --- Get predicted probabilities on the test set ---

pred_probs <- predict(svm_model, test_scaled, type = "prob")

# --- Compute ROC curve ---

roc_obj <- roc(

response = test_scaled$critical_dengue,

predictor = pred_probs$Yes, # probability for "Yes" class

levels = rev(levels(test_scaled$critical_dengue))

)

# --- Plot ROC curve ---

plot(roc_obj, col = "blue", lwd = 2, main = "ROC Curve for SVM (Test Set)")

abline(a = 0, b = 1, lty = 2, col = "gray") # diagonal reference line

# --- Print AUC ---

auc(roc_obj)

### 95% confidence intervals (CIs) for AUC and other performance metrics (SMV model above)

library(pROC)

library(caret)

# --- Predictions (class + probability) ---

pred_class <- predict(svm_model, test_scaled)

pred_probs <- predict(svm_model, test_scaled, type = "prob")

# --- Confusion matrix with accuracy, sensitivity, specificity ---

cm <- confusionMatrix(pred_class, test_scaled$critical_dengue, positive = "Yes")

cm

# --- ROC + AUC with 95% CI ---

roc_obj <- roc(

response = test_scaled$critical_dengue,

predictor = pred_probs$Yes, # Probabilities for "Yes"

levels = rev(levels(test_scaled$critical_dengue))

)

# Plot ROC curve

plot(roc_obj, col = "blue", lwd = 2, main = "ROC Curve with 95% CI (Test Set)")

abline(a = 0, b = 1, lty = 2, col = "gray")

# AUC and 95% CI

auc_val <- auc(roc_obj)

auc_ci <- ci.auc(roc_obj) # 95% CI for AUC

auc_val

auc_ci

# --- 95% CI for sensitivity, specificity, and accuracy ---

# Sensitivity

sens_ci <- ci.se(roc_obj, specificities = seq(0, 1, 0.1))

# Specificity (at given sensitivities)

spec_ci <- ci.sp(roc_obj, sensitivities = seq(0, 1, 0.1))

# Accuracy (from confusion matrix bootstrap)

acc <- cm$overall["Accuracy"]

acc_ci <- binom.test(cm$table[1,1] + cm$table[2,2], sum(cm$table))$conf.int

list(

AUC = auc_val,

AUC_CI = auc_ci,

Accuracy = acc,

Accuracy_CI = acc_ci,

Sensitivity_CI = sens_ci,

Specificity_CI = spec_ci

)

## 95% CI of AUC and performance metrics (SVM model):

## wrap this up into a single summary table (with metrics + 95% CIs):

library(caret)

library(pROC)

library(dplyr)

# --- Predictions ---

pred_class <- predict(svm_model, test_scaled)

pred_probs <- predict(svm_model, test_scaled, type = "prob")

# --- Confusion Matrix ---

cm <- confusionMatrix(pred_class, test_scaled$critical_dengue, positive = "Yes")

# --- ROC + AUC ---

roc_obj <- roc(

response = test_scaled$critical_dengue,

predictor = pred_probs$Yes,

levels = rev(levels(test_scaled$critical_dengue))

)

# --- AUC + 95% CI ---

auc_val <- as.numeric(auc(roc_obj))

auc_ci <- as.numeric(ci.auc(roc_obj))

# --- Accuracy + 95% CI ---

acc <- cm$overall["Accuracy"]

acc_ci <- binom.test(cm$table[1,1] + cm$table[2,2], sum(cm$table))$conf.int

# --- Sensitivity + 95% CI ---

sens <- cm$byClass["Sensitivity"]

sens_ci <- binom.test(cm$table[2,2], cm$table[2,1] + cm$table[2,2])$conf.int

# --- Specificity + 95% CI ---

spec <- cm$byClass["Specificity"]

spec_ci <- binom.test(cm$table[1,1], cm$table[1,1] + cm$table[1,2])$conf.int

# --- Build summary table ---

results <- data.frame(

Metric = c("Accuracy", "Sensitivity", "Specificity", "AUC"),

Estimate = c(acc, sens, spec, auc_val),

CI_Lower = c(acc_ci[1], sens_ci[1], spec_ci[1], auc_ci[1]),

CI_Upper = c(acc_ci[2], sens_ci[2], spec_ci[2], auc_ci[3])

)

# --- Nicely formatted table ---

results <- results %>%

mutate(

Estimate = round(Estimate, 3),

CI = paste0("(", round(CI_Lower, 3), " – ", round(CI_Upper, 3), ")")

) %>%

select(Metric, Estimate, CI)

print(results)

####

**## 6-6 KNN, K-nearest neighbors**

## --- Load packages ---

# install.packages(c("caret", "pROC", "binom", "epiR"))

library(caret)

library(pROC)

library(binom)

library(epiR)

## --- Import dataset ---

mydata <- read.csv("C:/Users/HP/Desktop/LALB.multiple.imputation.CSV")

## Factorize outcome

mydata$critical_dengue <- factor(mydata$critical_dengue,

levels = c(0,1),

labels = c("No","Yes"))

## Train/test split (caret method)

set.seed(123)

trainIndex <- createDataPartition(mydata$critical_dengue, p = 0.7, list = FALSE)

train <- mydata[trainIndex, ]

test <- mydata[-trainIndex, ]

## --- Preprocessing (scale numeric predictors for KNN) ---

preProcValues <- preProcess(train[, -24], method = c("center", "scale"))

train_scaled <- predict(preProcValues, train[, -24])

test_scaled <- predict(preProcValues, test[, -24])

## Add outcome back

train_scaled$critical_dengue <- train$critical_dengue

test_scaled$critical_dengue <- test$critical_dengue

## --- Train KNN model ---

set.seed(123)

knn_model <- train(

critical_dengue ~ .,

data = train_scaled,

method = "knn",

trControl = trainControl(method = "cv", number = 10),

tuneLength = 10

)

print(knn_model)

## --- Predictions on test set ---

knn_pred_class <- predict(knn_model, newdata = test_scaled)

knn_pred_prob <- predict(knn_model, newdata = test_scaled, type = "prob")[, "Yes"]

## Confusion Matrix + Accuracy, Sens, Spec

cm <- confusionMatrix(knn_pred_class, test_scaled$critical_dengue, positive = "Yes")

print(cm)

## --- Extract metrics ---

TP <- cm$table[2,2]

TN <- cm$table[1,1]

FP <- cm$table[1,2]

FN <- cm$table[2,1]

# Sensitivity, Specificity, Precision, Accuracy

sens <- TP / (TP + FN)

spec <- TN / (TN + FP)

prec <- TP / (TP + FP)

acc <- (TP + TN) / (TP + TN + FP + FN)

f1 <- 2 * (prec * sens) / (prec + sens)

metrics <- data.frame(

Metric = c("Accuracy","Sensitivity","Specificity","Precision","F1 Score"),

Estimate = c(acc, sens, spec, prec, f1)

)

print(metrics)

## --- 95% CI for metrics ---

# Accuracy CI

acc_ci <- binom.confint(TP+TN, TP+TN+FP+FN, method="wilson")

# Sensitivity CI

sens_ci <- binom.confint(TP, TP+FN, method="wilson")

# Specificity CI

spec_ci <- binom.confint(TN, TN+FP, method="wilson")

# Precision CI

prec_ci <- binom.confint(TP, TP+FP, method="wilson")

# F1 CI (bootstrap)

set.seed(123)

boot_f1 <- replicate(1000, {

idx <- sample(1:nrow(test_scaled), replace=TRUE)

y_true <- test_scaled$critical_dengue[idx]

y_pred <- knn_pred_class[idx]

cm_boot <- table(y_true, y_pred)

TPb <- cm_boot["Yes","Yes"]

FPb <- cm_boot["No","Yes"]

FNb <- cm_boot["Yes","No"]

prec_b <- TPb / (TPb + FPb)

sens_b <- TPb / (TPb + FNb)

if(is.nan(prec_b) | is.nan(sens_b)) return(NA)

2*(prec_b*sens_b)/(prec_b+sens_b)

})

boot_f1 <- na.omit(boot_f1)

f1_ci <- quantile(boot_f1, c(0.025, 0.975))

## --- AUC with 95% CI ---

roc_knn <- roc(test_scaled$critical_dengue, knn_pred_prob)

auc(roc_knn)

ci.auc(roc_knn)

plot(roc_knn, col="blue", lwd=2, main="ROC Curve - KNN")

## --- Final summary table ---

summary_table <- data.frame(

Metric = c("AUC","Accuracy","Sensitivity","Specificity","Precision","F1 Score"),

Estimate = c(auc(roc_knn), acc, sens, spec, prec, f1),

CI_Lower = c(ci.auc(roc_knn)[1], acc_ci$lower, sens_ci$lower, spec_ci$lower, prec_ci$lower, f1_ci[1]),

CI_Upper = c(ci.auc(roc_knn)[3], acc_ci$upper, sens_ci$upper, spec_ci$upper, prec_ci$upper, f1_ci[2])

)

print(summary_table)

###

**## 6-7 Naïve Bayes**

## --- Load packages ---

# install.packages(c("e1071", "caret", "pROC", "binom"))

library(e1071)

library(caret)

library(pROC)

library(binom)

## --- Import dataset ---

mydata <- read.csv("C:/Users/HP/Desktop/LALB.multiple.imputation.CSV")

## Factorize outcome

mydata$critical_dengue <- factor(mydata$critical_dengue,

levels = c(0,1),

labels = c("No","Yes"))

## --- Train/test split ---

set.seed(123)

trainIndex <- createDataPartition(mydata$critical_dengue, p = 0.7, list = FALSE)

train <- mydata[trainIndex, ]

test <- mydata[-trainIndex, ]

## --- Train Naïve Bayes model ---

set.seed(123)

nb_model <- naiveBayes(critical_dengue ~ ., data = train)

## --- Predictions ---

nb_pred <- predict(nb_model, newdata = test, type = "class")

nb_prob <- predict(nb_model, newdata = test, type = "raw")[, "Yes"]

## --- Confusion Matrix ---

cm <- confusionMatrix(nb_pred, test$critical_dengue, positive = "Yes")

print(cm)

## --- Extract metrics ---

TP <- cm$table[2,2]

TN <- cm$table[1,1]

FP <- cm$table[1,2]

FN <- cm$table[2,1]

sens <- TP / (TP + FN)

spec <- TN / (TN + FP)

prec <- TP / (TP + FP)

acc <- (TP + TN) / (TP + TN + FP + FN)

f1 <- 2 * (prec * sens) / (prec + sens)

metrics <- data.frame(

Metric = c("Accuracy","Sensitivity","Specificity","Precision","F1 Score"),

Estimate = c(acc, sens, spec, prec, f1)

)

print(metrics)

## --- 95% CI for metrics ---

acc_ci <- binom.confint(TP+TN, TP+TN+FP+FN, method="wilson")

sens_ci <- binom.confint(TP, TP+FN, method="wilson")

spec_ci <- binom.confint(TN, TN+FP, method="wilson")

prec_ci <- binom.confint(TP, TP+FP, method="wilson")

# Bootstrap CI for F1

set.seed(123)

boot_f1 <- replicate(1000, {

idx <- sample(1:nrow(test), replace=TRUE)

y_true <- test$critical_dengue[idx]

y_prob <- nb_prob[idx]

y_pred <- ifelse(y_prob > 0.5, "Yes","No")

y_pred <- factor(y_pred, levels = c("No","Yes"))

cm_boot <- table(y_true, y_pred)

TPb <- cm_boot["Yes","Yes"]

FPb <- cm_boot["No","Yes"]

FNb <- cm_boot["Yes","No"]

prec_b <- TPb / (TPb + FPb)

sens_b <- TPb / (TPb + FNb)

if(is.nan(prec_b) | is.nan(sens_b)) return(NA)

2*(prec_b*sens_b)/(prec_b+sens_b)

})

boot_f1 <- na.omit(boot_f1)

f1_ci <- quantile(boot_f1, c(0.025, 0.975))

## --- AUC with 95% CI ---

roc_nb <- roc(test$critical_dengue, nb_prob)

auc(roc_nb)

ci.auc(roc_nb)

plot(roc_nb, col="darkgreen", lwd=2, main="ROC Curve - Naïve Bayes")

## --- Final summary table ---

summary_table <- data.frame(

Metric = c("AUC","Accuracy","Sensitivity","Specificity","Precision","F1 Score"),

Estimate = c(auc(roc_nb), acc, sens, spec, prec, f1),

CI_Lower = c(ci.auc(roc_nb)[1], acc_ci$lower, sens_ci$lower, spec_ci$lower, prec_ci$lower, f1_ci[1]),

CI_Upper = c(ci.auc(roc_nb)[3], acc_ci$upper, sens_ci$upper, spec_ci$upper, prec_ci$upper, f1_ci[2])

)

print(summary_table)

##

1. **SHAP analysis and plot**

**Figure 3**-SHAP summary plot based on the XGBoost model, illustrating the relative contribution of serum lactate, lactate-derived ratios (LAR and L/B), and clinical features to the prediction of critical outcomes in pediatric patients with dengue shock syndrome.

# Python

*## Jupiter Notebook - SHAP for LAR-Lactate-LB model (Python)*

*### Upload required packages:*

**import** pandas **as** pd

**import** numpy **as** np

**import** matplotlib.pyplot **as** plt

**from** sklearn.experimental **import** enable_iterative_imputer

**from** sklearn.impute **import** IterativeImputer

**from** sklearn.model_selection **import** train_test_split

**from** sklearn.preprocessing **import** StandardScaler

**from** sklearn.linear_model **import** LogisticRegression

**from** sklearn.ensemble **import** RandomForestClassifier, AdaBoostClassifier

**from** sklearn.svm **import** SVC

**from** sklearn.neighbors **import** KNeighborsClassifier

**from** sklearn.naive_bayes **import** GaussianNB

**from** sklearn.metrics **import** roc_curve, auc

**from** xgboost **import** XGBClassifier

*# --- Load dataset ---*

data **=** pd**.**read_csv(r"C:\Users\HP\Desktop\LALB.multiple.imputation.csv", encoding**=**"latin1")

data**.**head()

*### 7-1- ALL SUPERVISED MODELS IN THIS STUDY*

*# === Step 1: Load data ===*

data **=** pd**.**read_csv(r"C:\Users\HP\Desktop\LALB.multiple.imputation.csv", encoding**=**"latin1")

*# === Step 2: Create composite target ===*

*# (Uncomment if needed)*

*# data['critical_dengue'] = ((data['MV'] == 1) | (data['death'] == 1) | (data['PALF'] == 1)).astype(int)*

*# === Step 3: Separate X and y ===*

y **=** data['critical_dengue']

X **=** data**.**drop(columns**=**'critical_dengue')

*# === Step 4: Encode categorical variables ===*

X **=** pd**.**get_dummies(X, drop_first**=True**)

*# === Step 5: Impute missing values ===*

imputer **=** IterativeImputer(random_state**=**42)

X_imputed **=** imputer**.**fit_transform(X)

X **=** pd**.**DataFrame(X_imputed, columns**=**X**.**columns)

*# === Step 6: Standardize features ===*

scaler **=** StandardScaler()

X_scaled **=** scaler**.**fit_transform(X)

*# === Step 7: Train/test split ===*

X_train, X_test, y_train, y_test **=** train_test_split(X_scaled, y, test_size**=**0.2, random_state**=**42)

*# === Step 8: Define models ===*

models **=** {

"Logistic Regression": LogisticRegression(solver**=**'liblinear', random_state**=**42),

"Random Forest": RandomForestClassifier(n_estimators**=**100, random_state**=**42),

"XGBoost": XGBClassifier(use_label_encoder**=False**, eval_metric**=**'logloss', random_state**=**42),

"AdaBoost": AdaBoostClassifier(n_estimators**=**100, random_state**=**42),

"SVM (RBF Kernel)": SVC(probability**=True**, kernel**=**'rbf', random_state**=**42),

"K-Nearest Neighbors": KNeighborsClassifier(n_neighbors**=**5),

"Naive Bayes": GaussianNB()

}

*# === Step 9: Plot ROC curves ===*

plt**.**figure(figsize**=**(10, 8))

**for** name, model **in** models**.**items():

model**.**fit(X_train, y_train)

y_proba **=** model**.**predict_proba(X_test)[:, 1]

fpr, tpr, _ **=** roc_curve(y_test, y_proba)

roc_auc **=** auc(fpr, tpr)

plt**.**plot(fpr, tpr, label**=**f"{name} (AUC = {roc_auc:.2f})")

plt**.**plot([0, 1], [0, 1], 'k--', label**=**"Random Guess")

plt**.**xlabel("False Positive Rate")

plt**.**ylabel("True Positive Rate")

plt**.**title("Combined ROC Curves for Ensemble, Logistic, SVM, and KNN Models")

plt**.**legend(loc**=**"lower right")

plt**.**grid(**True**)

plt**.**tight_layout()

*# Save the plot to desktop*

output_path **=** r"C:\Users\HP\Desktop\combined_roc_curves.png"

plt**.**savefig(output_path, dpi**=**300, bbox_inches**=**'tight')

plt**.**close()

print(f"ROC curves plot saved to: {output_path}")

*### 7-2 SHAP ANALYSIS WITH ENSEMBLE MODELS (XGBOOST, SVM) # Figure 3*

*## SHAP and XGboost*

*## Full Python Code: SHAP with XGBoost (Beeswarm Plot)*

**import** numpy **as** np

**import** pandas **as** pd

**import** shap

**import** matplotlib.pyplot **as** plt

**from** xgboost **import** XGBClassifier *# Import XGBoost classifier*

**from** sklearn.pipeline **import** Pipeline

**from** sklearn.impute **import** SimpleImputer

**from** sklearn.preprocessing **import** StandardScaler

**from** sklearn.model_selection **import** train_test_split

*# --- Load dataset ---*

data **=** pd**.**read_csv(r"C:\Users\HP\Desktop\LALB.multiple.imputation.csv", encoding**=**"latin1")

*# --- Set target column ---*

target_col **=** 'critical_dengue' *# 🔁 Change this if needed*

*# --- Separate features and target ---*

X **=** data**.**drop(columns**=**[target_col])

y **=** data[target_col]

*# --- Mean imputation for missing values ---*

imputer **=** SimpleImputer(strategy**=**'mean')

X_imputed **=** pd**.**DataFrame(imputer**.**fit_transform(X), columns**=**X**.**columns)

*# --- Train/test split ---*

X_train, X_test, y_train, y_test **=** train_test_split(

X_imputed, y, test_size**=**0.2, random_state**=**42

)

*# --- XGBoost pipeline with standardization ---*

xgb_pipeline **=** Pipeline([

('scaler', StandardScaler()),

('xgb', XGBClassifier(eval_metric**=**'logloss', use_label_encoder**=False**)) *# Disable label encoding*

])

*# --- Fit the model ---*

xgb_pipeline**.**fit(X_train, y_train)

*# --- SHAP Explainer for XGBoost ---*

X_test_scaled **=** xgb_pipeline**.**named_steps['scaler']**.**transform(X_test)

*# Use TreeExplainer which is designed for tree-based models like XGBoost*

explainer **=** shap**.**TreeExplainer(xgb_pipeline**.**named_steps['xgb'])

shap_values **=** explainer**.**shap_values(X_test_scaled)

*# --- Beeswarm plot (dot-based SHAP summary) ---*

shap**.**summary_plot(shap_values, features**=**X_test, feature_names**=**X**.**columns, plot_type**=**"dot")

1. **Calibration analysis and plot**

**Table 4**- Performance of predictive models with internal validation

**Figure 4**- **Calibration plots for prognostic models.** Calibration plots are presented for the (A) LAR-based, (B) lactate-based, and (C) LB-based models.

# R code

rm(list=ls()) #### Clear all working commands

## Import dataset:

LALB.multiple.imputation <- read.csv("C:/Users/HP/Desktop/LALB.multiple.imputation.csv")

### Copy and rename original dataset, "mydata"

mydata = LALB.multiple.imputation

### First look at dataset:

dim(mydata)

##Explore the data set = missing values

missing_data <- colSums(is.na(mydata))

missing_data

### Remove missing values if any # ensure no missing data in calibration analysis:

mydata <- na.omit(mydata)

library(dplyr)

glimpse(mydata)

dim(mydata) #### 524 (subjects) 24 (variables)

### Data preprocessing:

#### Log2 transformation IV fluid

mydata$log2.Fluid <-log2(mydata$Cumulative.fluid.24h+1)

library(ggpubr)

ggqqplot(mydata$log2.Fluid)

#### Factor categorical data:

### Study outcome

mydata$critical_dengue <- as.factor(mydata$critical_dengue)

### Study covariables

mydata$sex.female <- as.factor(mydata$sex.female)

mydata$DSS.Decompensated <- as.factor(mydata$DSS.Decompensated)

mydata$underlying.disease <- as.factor(mydata$underlying.disease)

mydata$onset.day.shock <- as.factor(mydata$onset.day.shock)

mydata$Severe.bleeding <- as.factor(mydata$Severe.bleeding)

mydata$PLT.lower20K <- as.factor(mydata$PLT.lower20K)

mydata$Severe.hepatitis <- as.factor(mydata$Severe.hepatitis)

mydata$AVPU.admission <- as.factor(mydata$AVPU.admission)

mydata$VIS.24h.over30 <- as.factor(mydata$VIS.24h.over30)

## Rename outcome = composite endpoint

mydata$Composite_Endpoint <- as.factor(mydata$critical_dengue)

### Calibration Analysis:

### https://cran.r-project.org/web/packages/ROCit/vignettes/my-vignette.html

### by Frank Harrel

## 8-1 LAR-based model

library(Hmisc)

library(rms)

ddist = datadist(mydata)

options(datadist = "ddist")

moboostrap1 = lrm(Composite_Endpoint~age.year + sex.female + DSS.Decompensated + underlying.disease +

onset.day.shock + Severe.bleeding + PLT.lower20K + PLT.transfusion +

Severe.hepatitis + VIS.24h.over30 + log2.Fluid+Res.rate + Sys.shock.index +

HCT.peak + HCT.nadir + INR + Creatinin + LA.admission

,data = mydata, x = TRUE, y = TRUE)

validate = validate(moboostrap1, B = 500)

validate

#### Calibration plot for model performance

cal = calibrate(moboostrap1, method = "boot", B=500)

plot(cal)

plot(cal,

xlab = "Predicted Probability",

ylab = "Observed Probability",

subtitles = FALSE,

lwd = 2,

col = "blue")

abline(0, 1, lty = 2, col = "gray") # 45° reference line

## 8-2 LACTATE-based model

library(Hmisc)

library(rms)

ddist = datadist(mydata)

options(datadist = "ddist")

moboostrap2 = lrm(Composite_Endpoint~age.year + sex.female + DSS.Decompensated + underlying.disease +

onset.day.shock + Severe.bleeding + PLT.lower20K + PLT.transfusion +

Severe.hepatitis + VIS.24h.over30 + log2.Fluid+Res.rate + Sys.shock.index +

HCT.peak + HCT.nadir + INR + Creatinin + Lactate.admission

,data = mydata, x = TRUE, y = TRUE)

validate = validate(moboostrap2, B = 500)

validate

#### Calibration plot for model performance

cal = calibrate(moboostrap2, method = "boot", B=500)

plot(cal)

plot(cal,

xlab = "Predicted Probability",

ylab = "Observed Probability",

subtitles = FALSE,

lwd = 2,

col = "blue")

abline(0, 1, lty = 2, col = "gray") # 45° reference line

## 8-3 LB ratio based model

library(Hmisc)

library(rms)

ddist = datadist(mydata)

options(datadist = "ddist")

moboostrap3 = lrm(Composite_Endpoint~age.year + sex.female + DSS.Decompensated + underlying.disease +

onset.day.shock + Severe.bleeding + PLT.lower20K + PLT.transfusion +

Severe.hepatitis + VIS.24h.over30 + log2.Fluid+Res.rate + Sys.shock.index +

HCT.peak + HCT.nadir + INR + Creatinin + LB.admission

,data = mydata, x = TRUE, y = TRUE)

validate = validate(moboostrap3, B = 500)

validate

#### Calibration plot for model performance

cal = calibrate(moboostrap3, method = "boot", B=500)

plot(cal)

plot(cal,

xlab = "Predicted Probability",

ylab = "Observed Probability",

subtitles = FALSE,

lwd = 2,

col = "blue")

abline(0, 1, lty = 2, col = "gray") # 45° reference line

1. **Decision curve analysis (DCA)**

**Figure 5- Decision curve analysis of prognostic models.** The LAR-, LB-, and lactate-based models showed net clinical benefit across a range of threshold probabilities, with the LAR-based model demonstrating the highest benefit.

# R code

rm(list=ls()) #### Clear all working commands

## Import dataset:

LALB.multiple.imputation <- read.csv("C:/Users/HP/Desktop/LALB.multiple.imputation.csv")

### Copy and rename original dataset, "mydata"

mydata = LALB.multiple.imputation

### First look at dataset:

dim(mydata)

##Explore the data set = missing values

missing_data <- colSums(is.na(mydata))

missing_data

### Remove missing values if any # ensure no missing data in calibration analysis:

mydata <- na.omit(mydata)

library(dplyr)

glimpse(mydata)

dim(mydata) #### 524 (subjects) 24 (variables)

### Data preprocessing:

#### Log2 transformation IV fluid

mydata$log2.Fluid <-log2(mydata$Cumulative.fluid.24h+1)

library(ggpubr)

ggqqplot(mydata$log2.Fluid)

#### Factor categorical data:

### Study outcome

#mydata$critical_dengue <- as.factor(mydata$critical_dengue) ### NOT factor outcome, format as 0/1

### Study covariables

mydata$sex.female <- as.factor(mydata$sex.female)

mydata$DSS.Decompensated <- as.factor(mydata$DSS.Decompensated)

mydata$underlying.disease <- as.factor(mydata$underlying.disease)

mydata$onset.day.shock <- as.factor(mydata$onset.day.shock)

mydata$Severe.bleeding <- as.factor(mydata$Severe.bleeding)

mydata$PLT.lower20K <- as.factor(mydata$PLT.lower20K)

mydata$Severe.hepatitis <- as.factor(mydata$Severe.hepatitis)

mydata$AVPU.admission <- as.factor(mydata$AVPU.admission)

mydata$VIS.24h.over30 <- as.factor(mydata$VIS.24h.over30)

## Rename outcome = composite endpoint

mydata$Composite_Endpoint <- mydata$critical_dengue

### Now performing DCA analysis

### 9-1 DCA for LAR model

## Decision Curve Analysis (DCA) ---

# rmda works with glm-style formulas

# If your outcome is Composite_Endpoint (binary 0/1)-as numeric format

library(rmda)

dca_model <- decision_curve(

Composite_Endpoint ~ age.year + sex.female + DSS.Decompensated + underlying.disease +

onset.day.shock + Severe.bleeding + PLT.lower20K + PLT.transfusion +

Severe.hepatitis + VIS.24h.over30 + log2.Fluid + Res.rate + Sys.shock.index +

HCT.peak + HCT.nadir + INR + Creatinin + LA.admission,

data = mydata,

family = binomial,

thresholds = seq(0.01, 0.99, by = 0.01),

confidence.intervals = 0.95,

study.design = "cohort" # important for clinical data

)

## --- Plot the Decision Curve ---

plot_decision_curve(

dca_model,

curve.names = "Full Model (with Lactate)",

xlab = "Threshold Probability",

ylab = "Net Benefit",

legend.position = "topright",

standardize = FALSE # set TRUE if you want standardized net benefit

)

abline(h = 0, lty = 2, col = "gray") # add reference

####

### WITHOUT LEGEND

plot_decision_curve(

dca_model,

curve.names = "Logistic Model",

xlab = "Threshold Probability",

ylab = "Net Benefit",

legend.position = "none"

)

### WITH LEGEND

plot_decision_curve(

dca_model,

curve.names = "Logistic Model",

xlab = "Threshold Probability",

ylab = "Net Benefit",

legend.position = "topright",

cex.legend = 0.5 # make legend smaller

)

###

### 9-2 DCA for LB model

## Decision Curve Analysis (DCA) ---

# rmda works with glm-style formulas

# If your outcome is Composite_Endpoint (binary 0/1)-as numeric format

library(rmda)

dca_model <- decision_curve(

Composite_Endpoint ~ age.year + sex.female + DSS.Decompensated + underlying.disease +

onset.day.shock + Severe.bleeding + PLT.lower20K + PLT.transfusion +

Severe.hepatitis + VIS.24h.over30 + log2.Fluid + Res.rate + Sys.shock.index +

HCT.peak + HCT.nadir + INR + Creatinin + LB.admission,

data = mydata,

family = binomial,

thresholds = seq(0.01, 0.99, by = 0.01),

confidence.intervals = 0.95,

study.design = "cohort" # important for clinical data

)

## --- Plot the Decision Curve ---

plot_decision_curve(

dca_model,

curve.names = "Full Model (with Lactate)",

xlab = "Threshold Probability",

ylab = "Net Benefit",

legend.position = "topright",

standardize = FALSE # set TRUE if you want standardized net benefit

)

abline(h = 0, lty = 2, col = "gray") # add reference

### WITHOUT LEGEND

plot_decision_curve(

dca_model,

curve.names = "Logistic Model",

xlab = "Threshold Probability",

ylab = "Net Benefit",

legend.position = "none"

)

### WITH LEGEND

plot_decision_curve(

dca_model,

curve.names = "Logistic Model",

xlab = "Threshold Probability",

ylab = "Net Benefit",

legend.position = "topright",

cex.legend = 0.7 # make legend smaller

)

### 9-3 LACTATE-based model

## Decision Curve Analysis (DCA) ---

# rmda works with glm-style formulas

# If your outcome is Composite_Endpoint (binary 0/1)-as numeric format

library(rmda)

dca_model <- decision_curve(

Composite_Endpoint ~ age.year + sex.female + DSS.Decompensated + underlying.disease +

onset.day.shock + Severe.bleeding + PLT.lower20K + PLT.transfusion +

Severe.hepatitis + VIS.24h.over30 + log2.Fluid + Res.rate + Sys.shock.index +

HCT.peak + HCT.nadir + INR + Creatinin + Lactate.admission,

data = mydata,

family = binomial,

thresholds = seq(0.01, 0.99, by = 0.01),

confidence.intervals = 0.95,

study.design = "cohort" # important for clinical data

)

## --- Plot the Decision Curve ---

plot_decision_curve(

dca_model,

curve.names = "Full Model (with Lactate)",

xlab = "Threshold Probability",

ylab = "Net Benefit",

legend.position = "topright",

standardize = FALSE # set TRUE if you want standardized net benefit

)

abline(h = 0, lty = 2, col = "gray") # add reference

### WITHOUT LEGEND

plot_decision_curve(

dca_model,

curve.names = "Logistic Model",

xlab = "Threshold Probability",

ylab = "Net Benefit",

legend.position = "none"

)

### WITH LEGEND

plot_decision_curve(

dca_model,

curve.names = "Logistic Model",

xlab = "Threshold Probability",

ylab = "Net Benefit",

legend.position = "topright",

cex.legend = 0.7 # make legend smaller

)

### 9-4-Comparison Between Three DCA Models

#### Three DCA models (dca_model_LAR, dca_model_LB, dca_model_lactate)

#### DCA curves for 3 models (in as single plot comparison)

# Load library

library(rmda)

# --- Fit 3 DCA models ---

### 9-4-1- LAR-based DCA model

dca_model_LA <- decision_curve(

Composite_Endpoint ~ age.year + sex.female + DSS.Decompensated + underlying.disease +

onset.day.shock + Severe.bleeding + PLT.lower20K + PLT.transfusion +

Severe.hepatitis + VIS.24h.over30 + log2.Fluid + Res.rate + Sys.shock.index +

HCT.peak + HCT.nadir + INR + Creatinin + LA.admission,

data = mydata,

family = binomial,

thresholds = seq(0.01, 0.99, by = 0.01),

confidence.intervals = 0.95,

study.design = "cohort"

)

###

### 9-4-2 Lactate-based DCA model

dca_model_lactate <- decision_curve(

Composite_Endpoint ~ age.year + sex.female + DSS.Decompensated + underlying.disease +

onset.day.shock + Severe.bleeding + PLT.lower20K + PLT.transfusion +

Severe.hepatitis + VIS.24h.over30 + log2.Fluid + Res.rate + Sys.shock.index +

HCT.peak + HCT.nadir + INR + Creatinin + Lactate.admission,

data = mydata,

family = binomial,

thresholds = seq(0.01, 0.99, by = 0.01),

confidence.intervals = 0.95,

study.design = "cohort"

)

### 9-4-3-LB-based DCA model

dca_model_LB <- decision_curve(

Composite_Endpoint ~ age.year + sex.female + DSS.Decompensated + underlying.disease +

onset.day.shock + Severe.bleeding + PLT.lower20K + PLT.transfusion +

Severe.hepatitis + VIS.24h.over30 + log2.Fluid + Res.rate + Sys.shock.index +

HCT.peak + HCT.nadir + INR + Creatinin + LB.admission,

data = mydata,

family = binomial,

thresholds = seq(0.01, 0.99, by = 0.01),

confidence.intervals = 0.95,

study.design = "cohort"

)

### Plot 3 Curves Of For Three DCA Models (LAR, Lactate and LB):

### WITHOUT LEGEND

plot_decision_curve(

list(dca_model_LA, dca_model_LB, dca_model_lactate),

curve.names = c("LAR", "LB", "Lactate"),

xlab = "Threshold Probability",

ylab = "Net Benefit",

lty = 1:3,

col = c("blue", "red", "green"),

legend.position = "none",

confidence.intervals = FALSE

)

### With Legend for 3 DCA curves:

legend("topright",

legend = c("LAR", "LB", "Lactate",

"Treat All", "Treat None"),

col = c("blue", "red", "darkgreen", "black", "black"),

lty = c(1, 2, 3, 1, 2),

cex = 0.8,

bty = "n")

dev.off()

# --- Export high-res PNG ---

png("C:/Users/HP/Desktop/DCA_3models_with95CI.png", width = 2000, height = 1600, res = 300)

# *This is all coding peformed for this study.*
