# Supplementary material for: Comparison of serum lactate and lactate-derived ratios as prognostic biomarkers in pediatric dengue shock syndrome using supervised machine learning models
Source: PLoS One. 2025 Oct 27;20(10):e0335022. doi: 10.1371/journal.pone.0335022 (PMC12558473; doi:10.1371/journal.pone.0335022)
Supplement: Attachment — Submitted filename: Response to PLOS Editors.docx [file pone.0335022.s012.docx]

**Response letter to editors**

Dear Editors-in-Chief of the PLOS ONE,

We are very thankful for your comments for our submitted manuscript.

**MANUSCRIPT # PONE-D-25-41174**, titled "Comparison of serum lactate and lactate-derived ratios as prognostic biomarkers in pediatric dengue shock syndrome using supervised machine learning models"

We are thankful for all peer-review points. These are our responses to all the reviewers’ comments.

**ACADEMIC EDITOR:**

1. Please upload copies of all the approval documents you received from your ethics committee to cover the entire range of the study period (i.e. the original approval document and any extension documents). Where ethics approval was obtained from more than one study location, please provide approval document(s) from all of the sites. If the original document is in another language, please also provide an English translation.

**Response:** Thanks for your comment. We have uploaded the local IRB Approval from Ethics Comitteee in Children’s Hospital 2, Ho Chi Minh City. The English attestation is available in the PLOS submission system. The document included Vietnamese and English translation in one single file (PDF).

2. please report in the Methods section the dates when data were accessed for research purposes in DD/MM/YYYY format.

**Response:** We are thankful for your comments, and appropriate amendments have been made to the revised manuscript. Lines 125-126 (highlighted below). This was amended in the revised manuscript submitted.

**Methods:**

**Data source, study setting and population**

“This study employed a secondary data analysis obtained from a previously approved investigation entitled *“*Prognostic model predicting mortality among patients presenting with dengue shock syndrome at Children’s Hospital 2, during 2013–2022*”* [6]. Ethical approval for the parent study was granted by the Scientific Committee and Institutional Review Board (IRB) of Children’s Hospital 2, Ho Chi Minh City, Vietnam (IRB approval number. 893/QD-BVND2; June 6, 2022) [6]. The requirement for informed consent was waived, and participants’ identities were anonymized to ensure patient confidentiality. The study was performed in accordance with the principles of Good Clinical Practice and ethical guidelines of the Declaration of Helsinki. The original dataset was accessed and analyzed on 01 May 2025.The current report adheres to the Transparent Reporting of a Multivariable Prediction Model for Individual Prognosis or Diagnosis (TRIPOD) guidelines (**S1 Checklist**).”

We have made appropriate amendment per your suggestion.

We look forward to your feedback and answer any further questions.

Many thanks,

Best regards,

Dr. Nguyen Tat Thanh
