# Supplementary material for: Comparison of serum lactate and lactate-derived ratios as prognostic biomarkers in pediatric dengue shock syndrome using supervised machine learning models
Source: PLoS One. 2025 Oct 27;20(10):e0335022. doi: 10.1371/journal.pone.0335022 (PMC12558473; doi:10.1371/journal.pone.0335022)
Supplement: Attachment — Submitted filename: Response to Reviewers.docx [file pone.0335022.s013.docx]

**Response letter to reviewers**

Dear Editors-in-Chief of the PLOS ONE,

We are very thankful for your insightful comments for our submitted manuscript.

MANUSCRIPT # **PONE-D-25-41174**, titled "Comparison of serum lactate and lactate-derived ratios as prognostic biomarkers in pediatric dengue shock syndrome using supervised machine learning models"

We are thankful for all peer-review points. These are our responses to all the reviewers’ comments.

**Academic Editors:**

**Journal Requirements:**

When submitting your revision, we need you to address these additional requirements.

1. Please ensure that your manuscript meets PLOS ONE's style requirements, including those for file naming. The PLOS ONE style templates can be found at

<https://journals.plos.org/plosone/s/file?id=wjVg/PLOSOne_formatting_sample_main_body.pdf> ;

[https://journals.plos.org/plosone/s/file?id=ba62/PLOSOne_formatting_sample_title_authors_affiliations.pdf](https://track.editorialmanager.com/CL0/https:%2F%2Fjournals.plos.org%2Fplosone%2Fs%2Ffile%3Fid=ba62%2FPLOSOne_formatting_sample_title_authors_affiliations.pdf/1/010f0198ade8360e-4953df76-59d1-409e-b51e-f4cd48b13603-000000/8FtovMgIzJHVmxoYRskF5SQA20eHZW2F6OHd70bp7D0=224)

**Response:** Yes, we are thankful for this comment. We have checked the journal format and have made appropriate amendments in the revised manuscript.

2. Please note that PLOS One has specific guidelines on code sharing for submissions in which author-generated code underpins the findings in the manuscript. In these cases, we expect all author-generated code to be made available without restrictions upon publication of the work.

**Response:** Thank you for this comment. We shared the code as appropriate request. The coding file has been submitted a as supplementary data (S2 Appendix).

3. We note that there is identifying data in the Supporting Information file <S1 Appendix.xlsx>. Due to the inclusion of these potentially identifying data, we have removed this file from your file inventory. Prior to sharing human research participant data, authors should consult with an ethics committee to ensure data are shared in accordance with participant consent and all applicable local laws.

Data that are not directly identifying may also be inappropriate to share, as in combination they can become identifying. For example, data collected from a small group of participants, vulnerable populations, or private groups should not be shared if they involve indirect identifiers (such as sex, ethnicity, location, etc.) that may risk the identification of study participants.

**Response:** Thank you for this comment. As per your suggestion, we have removed the participants’ identifier (ID) from the submitted dataset in compliance with the anonymized principle. Revised **S1 Appendix** (dataset) has been amended and resubmitted, as appropriate.

4. If the reviewer comments include a recommendation to cite specific previously published works, please review and evaluate these publications to determine whether they are relevant and should be cited. There is no requirement to cite these works unless the editor has indicated otherwise.

**Response:** Thank you for this comment. We have followed your instructions.

**Reviewer #1:**

1. **Title & Abstract —** Please temper superlatives such as “excellent performance” unless supported by external validation and demonstrated clinical utility; present all performance metrics with 95% confidence intervals using consistent precision (for example, AUC 0.82, 95% CI 0.78–0.86) and include PR-AUC given class imbalance; explicitly list the composite outcome components and total event count in the abstract; and state the rule used to choose the “optimal” LAR cutoff (e.g., Youden index), reporting sensitivity and specificity with 95% CIs at that threshold.

**Response:** Thank you for your comment and pointing this out. We have rechecked this statement in the Abstract and we will make an appropriate language style based on your suggestion.

The optimal LAR cutoff was chosen based on the Youden index criteria.

We will report sensitivity and specificity with 95% CIs at that threshold in the revised Table below in the revised manuscript.

**Table (Revised).** Area under the receiver operating characteristic curve for serum lactate, lactate-to-albumin and lactate-to-bicarbonate ratios, and cut-off points for composite clinical endpoint among children with dengue shock syndrome

| Parameters | AUC of composite clinical endpoint (95% CI) | | p*-*value | |
| --- | --- | --- | --- | --- |
| Lactate (mmol/L) | 0.72 (0.65 - 0.78) | | < 0.001 | |
| LAR | 0.82 (0.76 - 0.87) | | < 0.001 | |
| L/B ratio | 0.68 (0.62 - 0.74) | | < 0.01 | |
| Cutoff points | **Sensitivity** | **Specificity** | **LR (+)** | **LR (-)** |
| LAR ≥ 1.25 | 0.71  (0.60 – 0.80) | 0.82  (0.78 – 0.86) | 3.95  (3.35 – 4.65) | 0.36  (0.29 – 0.44) |
| L/B ratio ≥ 0.20 | 0.56  (0.45 – 0.67) | 0.75  (0.71 – 0.79) | 2.26  (1.84 – 2.79) | 0.58  (0.47 – 0.72) |

AUC, Area under the curve; LR, Likelihood ratio, 95% CI, Confidence interval (AUC, sensitivity, specificity, LR+, LR-); LAR, Lactate-to-albumin ratio; L/B, Lactate-to-bicarbonate ratio

**2. Introduction —** Clarify the biological and clinical rationale for why LAR may outperform lactate or L/B in DSS, briefly contrasting prior 24-hour findings with admission-time measurements; define each component of the composite endpoint (death, mechanical ventilation, PALF, encephalitis) and justify their aggregation from a clinical-decision perspective; and close with a precise objective that commits to assessing both discrimination and calibration while comparing LAR against lactate and L/B.

**Response:** Thank you for this comment.

- Your suggestion of “Clarifying the biological and clinical rationale for why LAR may outperform lactate or L/B in DSS, briefly contrasting prior 24-hour findings with admission-time measurements” should be placed in the discussion section rather than introduction. We will answer your queries from point to point in the following.

2.1 LAR may outperform lactate or L/B in DSS:

In fact, we discussed the suggested point in the paragraph 5**-in Discussion** of submitted manuscript.

*“*Notably, the superior prognostic performance of the lactate-to-albumin ratio (LAR) underscores its relevance in capturing the complex and interrelated pathophysiological processes underlying severe dengue shock syndrome. As the denominator of the ratio, serum albumin serves as a surrogate marker for the severity of plasma leakage, which is the hallmark of dengue pathogenesis. Given its biological half-life of approximately three weeks [28], a rapid and profound decline in albumin levels in the acute phase of DSS cannot be attributed solely to impaired hepatic synthesis or increased catabolism. Rather, this decline reflects substantial redistribution of albumin secondary to dengue-induced endothelial injury. This process may be further compounded by hemodilution resulting from aggressive fluid resuscitation, which has been associated with adverse clinical outcomes [4, 29]. Furthermore, serum lactate, the numerator of LAR, indicates the metabolic consequences of plasma leakage, including impaired perfusion, anaerobic metabolism, and systemic circulatory failure. In DSS, elevated lactate levels not only signal the presence of shock but also reflect inadequate tissue oxygenation despite fluid resuscitation and vasopressor support. Therefore, LAR captures both the underlying pathophysiological insult (plasma leakage resulting in hypoalbuminemia) and its clinical consequences (refractory shock, evidenced by hyperlactatemia). This integrated metric provides a more comprehensive representation of dengue severity than either lactate or albumin alone. High LAR values may indicate excessive capillary leakage or evolving multi-organ failure, particularly in patients with dengue-associated acute liver failure who present late in the course of irreversible shock. Accordingly, the capacity of LAR to quantify the interplay between vascular pathology and systemic perfusion failure likely underlies its superior prognostic value in predicting critical outcomes in pediatric DSS*.”*

2.2 Briefly contrasting prior 24-hour findings with admission-time point:

Considering the pathogenesis of dengue shock syndrome, the acute progression of dengue severity was calculated by hours after dengue shock occurrence after PICU admission. Hence, at 24h after admission, lactate, albumin, and bicarbonate levels significantly changed, corresponding to the severity of dengue shock. Significantly, it should be acknowledged that serum LAR, LB, and lactate values may evolve over the first 24 h owing to fluid resuscitation, hemodilution, and ongoing vascular leakage, which may modify their predictive utility over time after hospitalization. Thus, the 24h values of such biomarkers became clearly overt compared to the baseline values. Therefore, at 24h biomarkers of LAR, LB ratio, and lactate had higher predictive values. Notably, our research team has recently published and shown a higher predictive value of serum lactate and LB ratio at 24h post-admission than at the time of admission in this study. This further suggests dynamic modelling of these biomarkers during the first 24h of PICU admission.

2.3 Define each component of the composite endpoint (death, mechanical ventilation, PALF, encephalitis) and justify their aggregation from a clinical-decision perspective; and close with a precise objective that commits to assessing both discrimination and calibration while comparing LAR against lactate and L/B.

We have clearly defined the component of the composite endpoint (death, mechanical ventilation, PALF, and encephalitis) in the submitted manuscript.

The rationale for using the composite endpoint is the low proportion of deaths (mortality of dengue cases). We have shown in our previous publications that mechanical ventilation requirement and dengue-associated acute liver failure are the two most significant causes contributing to high mortality rates in patients with severe dengue. Therefore, it is reasonable to combine MV and PALF into a composite endpoint (with death outcome).

The discrimination and calibration of the LAR- and LB-based predictive models have been additionally performed and are presented in the revised manuscript and in the following.

**3. Methods:** Study Design & Cohort — Add a transparent flow diagram detailing numbers screened, excluded with reasons, and included; specify inclusion criteria, timing of blood sampling relative to admission, handling of any repeated measures, and follow-up window; provide exact outcome definitions (including whether mechanical ventilation includes non-invasive ventilation) and per-component counts; and state whether a protocol existed or confirm that no prior registration was performed.

**Response:** Thanks for your insightful comments.

3.1 Add a transparent flow diagram detailing numbers screened, excluded with reasons, and included; specify inclusion criteria

**Response**: Your suggested points are important; however, it is a gentle reminder that this was a secondary dataset research of a published retrospective study. Obtaining your suggested data (timing of blood sampling relative to admission, handling of any repeated measures, and follow-up window) is challenging and impractical.

Therefore, we simplified the study flow diagram, according with the availability of secondary dataset, as shown in **Fig 1.** in the submitted manuscript.

**Fig 1**

Notably, we emphasized that this was a secondary data analysis, and we found that the main reason of exclusion is missing data of biomarkers (LAR, LB, Lactate). Thus, we determined to keep this study flow diagram as it is (**Fig 1**).

\# Whether mechanical ventilation includes non-invasive ventilation:

**Response**: In this study, mechanical ventilation (MV) is exactly invasive MV.

Notably, nasal oxygenation and nasal continuous positive airway pressure (NCPAP) frequently used for DSS patients, were categorized as non-invasive ventilation (non-MV).

\# State whether a protocol existed or confirm that no prior registration was performed.

**Response:** In this study, the national dengue diagnosis and management protocol (the Vietnamese Ministry of Health) has been applied consistently over the study period and so far.

**4. Methods:** Predictors & Missing Data — Provide a complete list of candidate predictors with units and admission-time availability and include a data dictionary as supplementary material; characterize missingness at the variable and patient levels and discuss the likely mechanism (MAR/MNAR), supported by a missingness heatmap; perform imputation within the resampling loop so that models only “see” imputed values created from their training folds; justify the >20% missingness exclusion rule, list excluded variables, and present a complete-case sensitivity analysis.

**Response:** We are thankful for your insightful comment. For missing data (count and percentage), **S1 Fig**. (Supplementary File) was submitted to the journal. We have answered your point by point and performed the missing data analysis below.

We included all patients with biomarkers (albumin, lactate, bicarbonate) in the analysis. Patients who lacked biomarker data were excluded.

Complete-case data analysis was applied for the sensitivity/specificity analysis of biomarkers. A complete-case sensitivity has been analyzed and submitted in the revised manuscript (**Table 2**).

For supervised machine-learning models, we used multiple-imputation data, as appropriate.

Mechanism of missing data in this study: Missing Completely at Random (MCAR)

To the best of our knowledge, there is no “black-and-white” principle to choose the 20%, 30%... missingness threshold for the exclusion rule. Hence, we determined >20% missingness as the threshold,  because we mainly wanted the robust data for machine learning analysis to ensure the power and validity of study findings.

Based on your suggestion, we have added a missingness heatmap and Pareto chart (shown below), which will be submitted in the revision as supplementary data.

All predefined variables analyzed in the supervised models were complete (i.e no significant predictor in the supervised models was excluded).

**5. Methods:** Preprocessing & Modeling — Ensure that all preprocessing (scaling, one-hot encoding, class weighting or resampling, and any feature selection) is fit exclusively on training folds and then applied to held-out data; replace the single split with nested cross-validation or bootstrap optimism correction and, if feasible, add a temporal split (earlier years to train, later years to test) to probe transportability; report AUC, PR-AUC, Brier score, and expected calibration error with 95% CIs; include calibration-in-the-large and slope, add calibration plots, and consider isotonic or Platt recalibration; present decision-curve analysis to compare net benefit against treat-all/none policies and simple heuristics such as lactate alone; and show threshold-level confusion matrices, sensitivity, specificity, PPV, NPV, and likelihood ratios at clinically relevant cut points, specifying statistical tests (e.g., DeLong) and any multiplicity adjustments.

**Response:** Thank you for this comment. In current submission manuscript, we strictly performed most of what the reviewer mentioned above in data preprocessing and modeling. We ensure that all preprocessing (scaling, one-hot encoding, class weighting or resampling, and any feature selection) is fit exclusively on training folds and then applied to held-out data; the single split with nested cross-validation or bootstrap optimism correction. For the confusion matrices, statistical tests were performed using the DeLong method. Additionally, no interactions between the clinical predictors and biomarkers were found.

We performed additional analyses of the calibration plot and decision curve, and submitted these data in the revised manuscript. These data are shown below in question 6-**Result section**.

**6. Results:** Cohort & Outcomes — Provide a baseline characteristics table stratified by outcome with standardized mean differences to convey distributional separation; report per-component outcome counts and, where possible, univariable associations of LAR, lactate, and L/B with each component; when proposing cutoffs, give sensitivity/specificity with CIs and LR+/LR− alongside prevalence-adjusted PPV/NPV to guide bedside interpretation; and for machine-learning models, present outer-fold performance (mean ± SD), calibration results, and decision-curve plots.

**Response:** As the reviewer suggested, we further perform analysis of standardized mean differences using R package- *tableone* (version 4.5.0). We have provided baseline characteristic tables stratified by study outcomes with standardized mean differences (SMD) to convey distributional separation.

**6.1 Standardized mean differences of showing distribution separation:**

\# *Report per-component study outcome*:

1. **Composite endpoint:**

```
                                     Stratified by CompositeEndpoint
                                      0               1            SMD
  n                                   435             89
  age.year (mean (SD))               8.87 (3.58)    6.72 (3.47)    0.611
  sex.female = 1 (%)                  192 (44.1)      40 (44.9)    0.016
  DSS.Decompensated = 1 (%)            41 ( 9.4)      22 (24.7)    0.415
  underlying.disease = 1 (%)           39 ( 9.0)      12 (13.5)    0.143
  onset.day.shock = 1 (%)              47 (10.8)      23 (25.8)    0.396
  Severe.bleeding = 1 (%)               9 ( 2.1)      46 (51.7)    1.350
  PLT.lower20K = 1 (%)                132 (30.3)      27 (30.3)   <0.001
  PLT.transfusion = 1 (%)              26 ( 6.0)      62 (69.7)    1.741
  Severe.hepatitis = 1 (%)             22 ( 5.1)      52 (58.4)    1.399
  VIS.24h.over30 = 1 (%)                0 ( 0.0)      23 (25.8)    0.835
  Res.rate (mean (SD))              24.85 (5.34)   30.74 (7.95)    0.870
  Sys.shock.index (mean (SD))        1.31 (0.29)    1.48 (0.37)    0.517
  HCT.peak (mean (SD))              48.65 (5.12)   45.72 (7.36)    0.463
  HCT.nadir (mean (SD))             38.67 (4.67)   34.98 (7.37)    0.598
  INR (mean (SD))                    1.32 (0.38)    2.77 (1.96)    1.026
  Creatinin (mean (SD))             53.65 (12.27)  65.91 (43.24)   0.386
  Albumin.Admission (mean (SD))     28.76 (8.36)   21.01 (7.13)    0.997
  Lactate.admission (mean (SD))      2.48 (1.33)    4.88 (3.74)    0.854
  HCO3.admission (mean (SD))        16.67 (5.13)   15.87 (4.10)    0.173
  LA.admission (mean (SD))           0.94 (0.73)    2.65 (2.45)    0.942
  LB.admission (mean (SD))           0.23 (0.38)    0.37 (0.39)    0.357
  Cumulative.fluid.24h (mean (SD)) 145.63 (50.21) 221.30 (117.28)  0.839
```

**Note**s. Composite endpoint included in-hospital deaths, mechanical ventilation requirement and dengue-associated acute liver failure.

(Standardized Mean Difference (SMD) > 0.1 indicates imbalance between groups.)

2. **Study outcome = in-hospital death**

```
                                      Stratified by death
                                       0              1               SMD
  n                                   502             22
  age.year (mean (SD))               8.56 (3.65)    7.23 (3.43)    0.376
  sex.female = 1 (%)                  225 (44.8)       7 (31.8)    0.270
  DSS.Decompensated = 1 (%)            56 (11.2)       7 (31.8)    0.520
  underlying.disease = 1 (%)           46 ( 9.2)       5 (22.7)    0.377
  onset.day.shock = 1 (%)              67 (13.3)       3 (13.6)    0.008
  Severe.bleeding = 1 (%)              36 ( 7.2)      19 (86.4)    2.607
  PLT.lower20K = 1 (%)                149 (29.7)      10 (45.5)    0.330
  PLT.transfusion = 1 (%)              69 (13.7)      19 (86.4)    2.113
  Severe.hepatitis = 1 (%)             55 (11.0)      19 (86.4)    2.298
  VIS.24h.over30 = 1 (%)                7 ( 1.4)      16 (72.7)    2.190
  Res.rate (mean (SD))              25.55 (5.98)   32.39 (8.46)    0.933
  Sys.shock.index (mean (SD))        1.33 (0.31)    1.51 (0.35)    0.552
  HCT.peak (mean (SD))              48.29 (5.51)   45.09 (8.00)    0.466
  HCT.nadir (mean (SD))             38.19 (5.27)   34.50 (7.21)    0.585
  INR (mean (SD))                    1.45 (0.59)    4.39 (3.09)    1.321
  Creatinin (mean (SD))             54.30 (16.48)  88.38 (62.14)   0.749
  Albumin.Admission (mean (SD))     27.68 (8.62)   21.23 (7.49)    0.799
  Lactate.admission (mean (SD))      2.68 (1.67)    7.58 (5.01)    1.310
  HCO3.admission (mean (SD))        16.64 (5.00)   14.02 (3.45)    0.610
  LA.admission (mean (SD))           1.11 (1.07)    4.13 (3.14)    1.289
  LB.admission (mean (SD))           0.24 (0.37)    0.63 (0.52)    0.856
  Cumulative.fluid.24h (mean (SD)) 153.10 (64.75) 281.20 (115.73)  1.366
```

3. **Study outcome = mechanical ventilation requirement (MV)**

```
                                       Stratified by MV
                                       0              1            SMD
  n                                   439             85
  age.year (mean (SD))               8.85 (3.59)    6.71 (3.44)    0.610
  sex.female = 1 (%)                  194 (44.2)      38 (44.7)    0.010
  DSS.Decompensated = 1 (%)            41 ( 9.3)      22 (25.9)    0.445
  underlying.disease = 1 (%)           39 ( 8.9)      12 (14.1)    0.165
  onset.day.shock = 1 (%)              48 (10.9)      22 (25.9)    0.393
  Severe.bleeding = 1 (%)              10 ( 2.3)      45 (52.9)    1.375
  PLT.lower20K = 1 (%)                133 (30.3)      26 (30.6)    0.006
  PLT.transfusion = 1 (%)              26 ( 5.9)      62 (72.9)    1.884
  Severe.hepatitis = 1 (%)             25 ( 5.7)      49 (57.6)    1.346
  VIS.24h.over30 = 1 (%)                0 ( 0.0)      23 (27.1)    0.861
  Res.rate (mean (SD))              24.91 (5.37)   30.73 (8.05)    0.850
  Sys.shock.index (mean (SD))        1.31 (0.29)    1.48 (0.38)    0.521
  HCT.peak (mean (SD))              48.61 (5.13)   45.81 (7.47)    0.437
  HCT.nadir (mean (SD))             38.53 (4.92)   35.49 (6.90)    0.509
  INR (mean (SD))                    1.34 (0.40)    2.79 (2.00)    1.006
  Creatinin (mean (SD))             53.67 (12.35)  66.35 (44.02)   0.392
  Albumin.Admission (mean (SD))     28.68 (8.38)   21.05 (7.18)    0.978
  Lactate.admission (mean (SD))      2.50 (1.35)    4.90 (3.80)    0.842
  HCO3.admission (mean (SD))        16.68 (5.11)   15.77 (4.15)    0.196
  LA.admission (mean (SD))           0.96 (0.74)    2.66 (2.49)    0.925
  LB.admission (mean (SD))           0.23 (0.38)    0.38 (0.40)    0.368
  Cumulative.fluid.24h (mean (SD)) 145.36 (50.14) 226.28 (117.52)  0.896
```

4. **Study outcome = dengue-associated acute liver failure (PALF)**

```
                                     Stratified by PALF
                                       0              1             SMD
  n                                   483             41
  age.year (mean (SD))               8.62 (3.62)    7.22 (3.69)    0.381
  sex.female = 1 (%)                  217 (44.9)      15 (36.6)    0.170
  DSS.Decompensated = 1 (%)            54 (11.2)       9 (22.0)    0.293
  underlying.disease = 1 (%)           45 ( 9.3)       6 (14.6)    0.164
  onset.day.shock = 1 (%)              63 (13.0)       7 (17.1)    0.113
  Severe.bleeding = 1 (%)              24 ( 5.0)      31 (75.6)    2.075
  PLT.lower20K = 1 (%)                142 (29.4)      17 (41.5)    0.254
  PLT.transfusion = 1 (%)              54 (11.2)      34 (82.9)    2.067
  Severe.hepatitis = 1 (%)             36 ( 7.5)      38 (92.7)    3.259
  VIS.24h.over30 = 1 (%)                4 ( 0.8)      19 (46.3)    1.270
  Res.rate (mean (SD))              25.37 (5.77)   31.43 (8.66)    0.824
  Sys.shock.index (mean (SD))        1.32 (0.30)    1.52 (0.41)    0.572
  HCT.peak (mean (SD))              48.37 (5.39)   45.67 (7.87)    0.400
  HCT.nadir (mean (SD))             38.43 (4.93)   33.47 (8.06)    0.743
  INR (mean (SD))                    1.39 (0.45)    3.76 (2.48)    1.332
  Creatinin (mean (SD))             53.71 (13.39)  79.49 (56.92)   0.623
  Albumin.Admission (mean (SD))     27.93 (8.59)   21.31 (7.17)    0.837
  Lactate.admission (mean (SD))      2.62 (1.58)    6.13 (4.37)    1.070
  HCO3.admission (mean (SD))        16.66 (5.04)   15.02 (3.83)    0.367
  LA.admission (mean (SD))           1.06 (1.01)    3.28 (2.73)    1.076
  LB.admission (mean (SD))           0.24 (0.38)    0.48 (0.44)    0.585
  Cumulative.fluid.24h (mean (SD)) 150.94 (61.70) 247.39 (116.31)  1.036
```

**6.2 Univariable associations of LAR, lactate and L/B with each component:**

We additionally performed analysis and the results are shown below.

**Table 1. The unadjusted associations of biomarkers and composite endpoint**

| *Composite clinical endpoint* | | | |
| --- | --- | --- | --- |
| **Variables** | **Odds ratio** | **95% CI** | **p-value** |
| LAR (+ 1 unit) | 2.84 | 2.14 – 3.78 | < 0.001 |
| Lactate (+1 mmol/L) | 1.54 | 1.36 – 1.74 | < 0.001 |
| LB (+ 1 unit) | 2.05 | 1.23 – 3.41 | < 0.01 |

**Table 2. The unadjusted associations of biomarkers and in-hospital mortality**

| *In-hospital mortality* | | | |
| --- | --- | --- | --- |
| **Variables** | **Odds ratio** | **95% CI** | **p-value** |
| LAR (+ 1 unit) | 1.82 | 1.5 – 2.21 | < 0.001 |
| Lactate (+1 mmol/L) | 1.56 | 1.38 – 1.77 | < 0.001 |
| LB (+ 1 unit) | 3.1 | 1.63 – 5.88 | < 0.001 |

**Table 3. The unadjusted associations of biomarkers and requirement for mechanical ventilation**

| *Requirement for mechanical ventilation support* | | | |
| --- | --- | --- | --- |
| **Variables** | **Odds ratio** | **95% CI** | **p-value** |
| LAR (+ 1 unit) | 2.62 | 2 – 3.43 | < 0.001 |
| Lactate (+1 mmol/L) | 1.52 | 1.35 – 1.71 | < 0.001 |
| LB (+ 1 unit) | 2.08 | 1.24 – 3.47 | < 0.01 |

**Table 4. The unadjusted associations of biomarkers and dengue-associated acute liver failure (PALF)**

| *Dengue-associated PALF* | | | |
| --- | --- | --- | --- |
| **Variables** | **Odds ratio** | **95% CI** | **p-value** |
| LAR (+ 1 unit) | 1.88 | 1.54 – 2.3 | < 0.001 |
| Lactate (+1 mmol/L) | 1.52 | 1.35 – 1.7 | < 0.001 |
| LB (+ 1 unit) | 2.51 | 1.42 – 4.44 | < 0.01 |

95% CI, Confidence interval, LAR, Lactate-to-albumin ratio; L/B, Lactate-to-bicarbonate ratio

Notably, only 4 patients were diagnosed with dengue-associated encephalitis, which has been reported to have poor clinical and survival outcomes in the medical literature. Due to the very limited number of encephalitis cases (4/524 patients), we did not present univariable analysis between dengue-associated encephalitis and biomarkers (LAR, Lactate and LB), considering that it may be be too extreme in terms of statistical calculation.

In this regard, we have amended the **Table 1** in the revised manuscript, as shown below:

**Table 1 (Revised).** Baseline clinical and laboratory characteristics of study participants on admission and clinical outcomes at discharge

| **Characteristics** | **All participants**  **(N = 524)** | **Composite endpoint (+)**  **(n = 89)** | **Composite endpoint (-)**  **(n = 435)** | **p-value** |
| --- | --- | --- | --- | --- |
| Age (years) | 8.7 (6-11) | 7 (4.2-9.1) | 9 (6.5-11.4) | < 0.001 |
| Female patients, n (%) | 232 (44) | 40 (45) | 192 (44) | 0.98 |
| Body mass index (kg/m^2^) | 18.9  (16.3-22.5) | 18.9  (16.5-22.5) | 18.9  (16.3-22.3) | 0.55 |
| Underlying diseases, n (%) | 51 (9.7) | 12 (14) | 39 (9) | 0.26 |
| Early onset of dengue shock (< day 4), n (%) | 70 (13.4) | 23 (26) | 47 (11) | < 0.001 |
| Grade of DSS severity, n (%)  Compensated DSS  Decompensated DSS | 461 (88)  63 (12) | 67 (75.3)  22 (24.7) | 394 (90.6)  41 (9.4) | < 0.001 |
| Severe bleeding, n (%) | 55 (10.5) | 46 (51.7) | 9 (2.1) | < 0.001 |
| Systolic shock index (bpm/mmHg) | 1.3 (1.1-1.5) | 1.4 (1.3-1.7) | 1.3 (1.1-1.4) | 0.001 |
| Respiratory rate (/min) | 24 (22-28) | 30 (25-36) | 24 (20-26) | < 0.001 |
| Neurological status (on AVPU scale), n (%)  Alert and verbal response  Pain and unresponse | 492 (94)  32 (6) | 58 (65)  31 (35) | 434 (99.7)  1 (0.3) | < 0.001 |
| White blood cell count  (x 10^9^/L) | 5.19  (3.71-7.52) | 7.4  (4.6-10.8) | 4.83  (3.6-6.9) | < 0.001 |
| Hemoglobin (g/dL) | 14.8  (13.3-16.2) | 13.6  (11.6-15) | 15.1  (13.6-16.3) | < 0.001 |
| Peak hematocrit (%) | 48 (45-52) | 47 (42-51) | 48 (46-52) | 0.001 |
| Nadir hematocrit (%) | 39 (35-41) | 36 (30-41) | 39 (36-42) | < 0.001 |
| Platelet count (x 10^9^/L) | 30 (16-49) | 29 (15-48) | 31 (16-49) | 0.64 |
| Platelet transfusion, n (%) | 88 (17) | 62 (70) | 26 (6) | < 0.001 |
| Aspartate aminotransferase (IU/L) | 148  (77-398) | 1161  (291-3158) | 125  (73-356) | < 0.001 |
| Alanine aminotransferase (IU/L) | 64  (31-202) | 412  (123-1053) | 54  (28-118) | < 0.001 |
| Severe transaminitis, n (%) | 74 (14.1) | 52 (58.4) | 22 (5.1) | < 0.001 |
| International normalized ratio (INR) | 1.28 (1.15-1.62) | 2.26 (1.77-3.0) | 1.22  (1.13-1.43) | < 0.001 |
| Serum creatinine (µmol/L) | 53 (45-61) | 57 (43-68) | 53 (45-60) | < 0.01 |
| Serum albumin (g/dL) | 2.8 (1.9-3.4) | 2.0 (1.5-2.7) | 3.1 (2.2-3.6) | < 0.001 |
| Serum lactate (mmol/L) | 2.30 (1.7-3.2) | 3.5 (2.1-6.5) | 2.2 (1.6-3.0) | < 0.001 |
| LAR | 0.86  (0.61-1.3) | 1.77  (0.91-3.38) | 0.78  (0.57-1.09) | < 0.001 |
| LB ratio | 0.14  (0.09-0.23) | 0.23  (0.13-0.4) | 0.13  (0.09-0.2) | < 0.01 |
| Arterial blood gas analysis  pH  PCO_2_ (mmHg)  PO_2_ (mmHg)  Fraction inspired oxygen (%)  Bicarbonate (mEq/L) | 7.43 (7.39-7.47)  27 (22-32)  93 (53-139)  32 (22-36)  17.1 (14.7-19.4) | 7.38 (7.3-7.45)  27 (22-34)  139 (101-170)  60 (32-60)  16 (12.8-18.1) | 7.44 (7.4-7.47)  26 (22-31)  81 (49-128)  32 (21-32) 17.4 (14.8-20) | < 0.001  0.04  < 0.001  < 0.001  0.11 |
| Cumulative fluid infused from referral hospitals and 24h PICU admission, (mL/kg) | 143  (111-190) | 209  (136-303) | 137  (107-176) | < 0.001 |
| Vasoactive inotropic score within 6h of admission | 0 (0-0) | 15 (0-30) |  | < 0.001 |
| ***Patient complications and outcomes at discharge*** | | | | |
| Mechanical ventilation requirement, n (%) | 85 (16.2) | 85 (96) | 0 (0) | < 0.001 |
| Acute liver failure, n (%) | 41 (7.8) | 41 (46) | 0 (0) | < 0.001 |
| Dengue-associated encephalitis, n (%) | 4 (0.8) | 4 (4.5) | 0 (0) | 0.001 |
| Fatal outcome, n (%) | 22 (4.2) | 22 (24.7) | 0 (0) | < 0.001 |

Summary statistics are presented as median (interquartile) for continuous variables and frequency (%) for categorical variables. Two-sided, two-sample t tests were used for comparison of continuous variables, and the Chi-square test (adjusted Fisher exact test) were applicable categorical group comparisons. DSS, dengue shock syndrome; LAR, Lactate-to-albumin ratio; L/B, Lactate-to-bicarbonate ratio

**6.3 Calibration analysis results:**

Calibration plot: PR-AUC, Brier score, and expected calibration error with 95% CIs; include calibration-in-the-large and slope, add calibration plots.

**Response:** We have performed calibration analysis and input in the revision (**Table 4**).

**Table 4. Performance of predictive models with internal validation**

| Models | C-statistic | Calibration-in-the-large | Calibration slope | Brier score |
| --- | --- | --- | --- | --- |
| LAR | 0.96 (0.93) | 0 (-0.28) | 1 (0.72) | 0.03 (0.04) |
| Lactate | 0.96 (0.93) | 0 (-0.27) | 1 (0.74) | 0.03 (0.04) |
| LB | 0.95 (0.93) | 0 (-0.25) | 1 (0.76) | 0.04 (0.05) |

Statistics are summarized for training (test) sets performed with bootstrap (n =500 resampling) and internal validation. LAR, Lactate-to-albumin ratio; LB, Lactate-to-bicarbonate ratio

**Models**

A. LAR-based full predictive model (with all covariates from supervised models)
B. Lactate-based full predictive model (with all covariates from supervised models)
C. LB-based full predictive model (with all covariates from supervised models)

**Combining the three calibration data above in a single plot for visual evaluation:**

We can see that lactate-based predictive model had a low Brier score (0.04) and slightly better calibration plot and, than LAR and LB models. Notably, three models have mostly equivalent c-statistics and Brier score.

**6.4 Decision-curve analysis to compare net benefit against treat-all/none policies.**

The decision curve analysis indicated that the use of LAR, LB, and lactate models may confer a net clinical benefit across a broad range of threshold probabilities. Significantly, LAR-based model had the highest net benefit than LB- and lactate-based models. These results suggest that such biomarkers could have clinical utility in the management of severe dengue, although their applicability should be further validated in prospective studies.

**7. Results:** Comparative Performance — When stating that LAR outperformed lactate and L/B, quantify the difference using ΔAUC with 95% CIs and p-values, and corroborate with PR-AUC due to class imbalance; probe robustness with complete-case analyses and, if available, a temporal split; and assess whether apparent gains are driven largely by one component of the composite by providing per-component performance or sensitivity analyses that exclude a dominant component.

**Response:** Thank you for your insightful comments. We adopt a different approach from the clinician’s perspective. Therefore, we do not aim to perform a complicated analysis. However, we will have a modest statement that LAR *performed better* than lactate and L/B ratio in the revised manuscript.

“*LAR exceeded serum lactate and the L/B ratio in predicting critical outcomes in pediatric DSS*.“

**8.** **Discussion —** Interpret the findings with calibrated language emphasizing that admission LAR is an informative early marker while acknowledging that later (e.g., 24-hour) measurements may differ; relate LAR’s performance to plausible DSS pathophysiology in which albumin reflects inflammatory, nutritional, and capillary-leak states beyond lactate alone; discuss the implications and potential drawbacks of predicting a heterogeneous composite versus component-specific endpoints; and translate decision-curve results into actionable scenarios such as ICU escalation, intensified monitoring, or early hepatology consultation.

**Response:** We are thankful for the insightful comments. Yes, we have made amendments per your suggestion in the Discussion section of the revised manuscript, as shown below.

**“**On admission LAR appears to be an informative early marker of poor outcomes in children with DSS, capturing critical pathophysiological processes that extend beyond hyperlactatemia alone. While elevated lactate levels reflect impaired perfusion and anaerobic metabolism, concurrent hypoalbuminemia at admission likely reflects the combined effects of systemic inflammation, capillary leak, and underlying nutritional status. This broader biological context may explain the higher discriminatory performance of LAR compared to that of lactate or the L/B ratio, particularly at the time of in-hospital presentation. Most significantly, it should be acknowledged that LAR, L/B, and lactate values may evolve over the first 24 h owing to fluid resuscitation, hemodilution, and ongoing vascular leakage, which could modify their predictive utility over time after hospitalization. Notably, our recent report showed a higher predictive value of lactate level and L/B ratio at 24h post-admission than at the time of admission in this study. Therefore, this suggests dynamic modelling of these biomarkers during the first 24h of PICU admission. An additional consideration is that prognostication against a heterogeneous composite endpoint may obscure differential predictive performance for specific complications, such as dengue-associated acute liver failure or the need for mechanical ventilation. These findings support the potential clinical relevance of LAR while highlighting the need for further validation across settings and outcome definitions. Another significant point is that the decision curve analysis incorporating admission LAR into early risk stratification may yield actionable benefits, such as timely intensive care escalation, prioritizing intensified hemodynamic monitoring, and prompting early consultation for patients at the greatest risk.”

**9. Limitations** — Expand the section to note risks of overfitting without nested or temporal validation, the potential bias introduced by excluding variables with >20% missingness, the clinical heterogeneity within the composite endpoint, the single-center design with evolving practice patterns over 2013–2022, and measurement variability in albumin/lactate assays; explicitly state that external validation is needed before clinical deployment.

**Response:** Thank you for this comment. Most of these points have been stated in the first version of our manuscript. We have also made appropriate amendments based on your suggestions and added them in the revised manuscript.

**10. Figures, Tables, and Supplement** — Make figure captions fully self-contained by defining all acronyms and describing each panel; ensure clear axes, units, and readable fonts with consistent significant digits; add figures for the patient flow, missingness structure, calibration curves, decision-curve analysis, SHAP explainability, and threshold-level confusion matrices; and include a supplement with full model specifications, hyperparameter grids, per-fold results, software and exact package versions, and reproducible scripts or notebooks.

**Response:** Thank you for your comment. As per your suggestions for figures for patient flow, missingness structure, calibration curves, decision curve analysis, SHAP explain ability, and threshold-level confusion matrices, we have made appropriate amendments in the revised manuscript.

10.1 Supplement data with full model specifications, hyperparameter grids, per-fold results, software and exact package versions, and reproducible scripts or notebooks.

**#** **Full predictive model specification:**

**S3 Table. The unadjusted associations between the covariables and composite endpoint**

| **Variables** | **Statistics** | **OR** | **95% CI** | **p-value** |
| --- | --- | --- | --- | --- |
| Lactate (+1 mmol/L) | 2.3 (1.7-3.2) | 1.54 | 1.36 – 1.74 | < 0.001 |
| LAR (+1 unit) | 0.86 (0.61-1.30) | 2.84 | 2.14 – 3.78 | < 0.001 |
| LB (+1 unit) | 0.14 (0.09-0.23) | 2.05 | 1.23 – 3.41 | < 0.01 |
| Age (years) | 8.7 (6-11) | 0.85 | 0.79-0.91 | < 0.001 |
| Female patients, n (%) | 232 (44.3) | 1.03 | 0.65-1.63 | 0.89 |
| Underlying diseases, n (%) | 51 (9.7) | 1.58 | 0.79-3.16 | 0.19 |
| Early onset of dengue shock ≤ 4 days, n (%) | 70 (13.4) | 2.88 | 1.64-5.05 | < 0.001 |
| Decompensated shock, n (%) | 63 (12) | 3.16 | 1.77-5.63 | < 0.001 |
| Respiratory rate (/min) | 24 (22-28) | 1.13 | 1.09-1.18 | < 0.001 |
| Systolic shock index (+1 unit) | 1.3 (1.11-1.5) | 5.07 | 2.31-11.1 | < 0.001 |
| Pain and Unresponsive on AVPU scale, n (%) | 32 (6.1) | 232 | 31.1-1731 | < 0.001 |
| Severe bleeding, n (%) | 55 (10.5) | 50.5 | 23.2-110.2 | < 0.001 |
| Severe transaminitis, n (%) | 74(14.1) | 26.4 | 14.5-48.1 | < 0.001 |
| Peak hematocrit (%) | 48 (45-52) | 0.92 | 0.88-0.95 | < 0.001 |
| Nadir hematocrit (%) | 39 (35-41) | 0.89 | 0.85-0.93 | < 0.001 |
| Serum creatinine (µmol/L) | 53 (45-61) | 1.02 | 1.01-1.04 | < 0.001 |
| Serum albumin (g/dL) | 2.8 (2.0-3.5) | 0.89 | 0.86-0.92 | < 0.001 |
| Serum bicarbonate (mEq/L) | 17.1 (14.7-19.5) | 0.97 | 0.93-1.01 | 0.18 |
| Low platelet count  (<20 x 10^9^/L), n (%) | 159 (30.3) | 0.99 | 0.61-1.64 | 0.99 |
| Requiring platetet transfusion | 88 (16.8) | 36.1 | 19.8-65.9 | < 0.001 |
| International normalized ratio | 1.3 (1.2-1.6) | 16.6 | 9.1-30.5 | < 0.001 |
| Cumulative amount of fluid infused from referral hospitals and 24h admission (mL/kg) | 143 (110-190) | 1.01 | 1.01-1.02 | < 0.001 |

Statistics are presented as median (interquartile range) for continuous variables and n (%) for categorical variables. CI, 95% confidence interval; LAR, Lactate-to-albumin ratio; LB, Lactate-to-bicarbonate ratio

The vasoactive inotropic score (in terms of VIS > 30 and log2-VIS score) within 24h admission both showed very large values (extremity) in the statistical calculation, indicating that VIS > 30 perfectly predicted the study outcome (p < 0.001).

Notably, these covariables were predetermined based on our clinical knowledge and a medical literature review [*References* 1-5 below]. Several covariables were not statistically significant; nevertheless, they were retained in the full model due to their clinical and pathogenesis importance and medical literature.

We have added this supplementary data - **S3 Table** in the revised manuscript

10.2 Hyperparameter grids, per-fold results, software and exact package versions, and reproducible scripts or notebooks.

**Response:** Hyperparameter tuning was performed for supervised models using a grid search strategy with 5-fold cross-validation to minimize overfitting and optimize generalizability. For the random forest, the number of trees, maximum tree depth, and minimum number of samples required for node splitting were tuned. For the support vector machine, the penalty parameter (C), kernel type, and kernel-specific parameters (e.g., gamma for radial basis function kernels) were adjusted. For the XGBoost model, the tuning included the learning rate, number of boosting iterations, maximum tree depth, and subsampling ratios. The optimal hyperparameters were selected based on cross-validated performance metrics.

10.3 Reproducible scripts or notebooks: submitted as a supplementary file (**S2 Appendix**)

10.4 R and Python packages used in the study analysis

- **R (version 4.5.0) used for analysis** - with packages including *table1, compareGroups, tableone, flextable, gtsummary, tidyverse, dplyr, epiDisplay,* *ggplot2, pROC, cutpointr, epiR, binom, glmnet,* *leaps, MASS, naniar, VIM, missRanger,* *caret, caTools, pls, vip, randomForest, mlr, e1071, xgboost,* *SHAPforxgboost, iml,* *Hmisc, rms, and* *rmda*.
- **Python (Anaconda Distribution version 3.0)** - the following packages were used including

*numpy, pandas, scikit-learn* (for model training and performance metrics); *xgboost* and *lightgbm* (for ensemble boosting models; and shap for model explainability).

Data visualization and preprocessing were conducted using *pandas, matplotlib, and seaborn*.

Supplement data with statistical software and exact package versions, and reproducible scripts or notebooks => these data have been submitted in the revision as the file - **S2 Appendix**.

**References**

[1] Thanh NT, Luan VT, Viet DC, Tung TH, Thien V. A machine learning-based risk score for prediction of mechanical ventilation in children with dengue shock syndrome: A retrospective cohort study. PLoS One. 2024 Dec 6;19(12):e0315281. doi: 10.1371/journal.pone.0315281. PMID: 39642139; PMCID: PMC11623794.

[2] Nguyen Tat T, Vo Hoang-Thien N, Nguyen Tat D, Nguyen PH, Ho LT, Doan DH, et al. Prognostic values of serum lactate-to-bicarbonate ratio and lactate for predicting 28-day in-hospital mortality in children with dengue shock syndrome. Medicine (Baltimore). 2024 Apr 26;103(17):e38000. doi: 10.1097/MD.0000000000038000. PMID: 38669370; PMCID: PMC11049702.

[3] Vo LT, Do VC, Trinh TH, Nguyen TT. In-Hospital Mortality in Mechanically Ventilated Children With Severe Dengue Fever: Explanatory Factors in a Single-Center Retrospective Cohort From Vietnam, 2013-2022. Pediatr Crit Care Med. 2025 Jun 1;26(6):e796-e805. doi: 10.1097/PCC.0000000000003728. Epub 2025 Mar 19. PMID: 40105396; PMCID: PMC12133049.

[4] Nguyen TT, Ngo PT, Vo LT. Predicting the risk of mortality in children with dengue-induced hepatitis admitted to the paediatric intensive care unit. World J Crit Care Med. 2024 Dec 9;13(4):98862. doi: 10.5492/wjccm.v13.i4.98862. PMID: 39655306; PMCID: PMC11577541.

[5] Huy BV, Toàn NV. Prognostic indicators associated with progresses of severe dengue. PLoS One. 2022 Jan 5;17(1):e0262096. doi: 10.1371/journal.pone.0262096. PMID: 34986174; PMCID: PMC8730386.

**11. Data Availability & Reproducibility —** Revise the Data Availability Statement to meet PLOS ONE’s policy by depositing a de-identified dataset with an accompanying data dictionary and the full analysis code in a public repository (e.g., OSF or Zenodo with a DOI, and code mirrored on GitHub with a release archived to Zenodo); if any restrictions apply, specify them precisely and provide a compliant access mechanism; and document random seeds, software versions, and hardware used to ensure reproducibility.

**Response:** Thank you for this comment. We have made appropriate amendments and submitted them for revision. In terms of restrictions, we were unfamiliar with the GitHub/Zenodo. Therefore, we have further submitted the full analysis code in a supplementary file (**S2 Appendix)**.

**12. Ethics & Governance —** Provide the institutional review board approval identifier and date, clarify consent or waiver status, and summarize de-identification and secure data handling procedures; disclose funding sources and any conflicts of interest to ensure transparency regarding potential influences on study design, analysis, or interpretation.

**Response:** Thank you for this comment.

We already submitted the translation and attestation of local institutional review board approval identifier and date, from the published primary research project, titled *"* Prognostic model predicting mortality among patients presenting with dengue shock syndrome at Children’s Hospital 2, 2013–2022*"* [*Reference* 1]. The original study was reviewed and approved by the Scientific Committee and Institutional Review Board (IRB) of Children's Hospital 2, Ho Chi Minh City, Vietnam (**Approval number. 893/QD-BVND2 on June 6, 2022**).

**Ethics statement**

“*This study is a secondary analysis of data derived from the primary research project titled "Prognostic model predicting mortality among patients presenting with dengue shock syndrome at Children’s Hospital 2, 2013–2022". The original study was reviewed and approved by the Scientific Committee and Institutional Review Board (IRB) of Children's Hospital 2, Ho Chi Minh City, Vietnam (Approval number. 893/QD-BVND2 on June 6, 2022). All procedures involving human participants were conducted in accordance with the ethical standards of the institutional and/or national research committee and the principles outlined in the Declaration of Helsinki (as revised in 2013). The study also adhered to the ethical requirements for medical research outlined by the International Committee of Medical Journal Editors (ICMJE). Given the retrospective nature of the study and the use of fully de-identified secondary data, the research involved minimal risk to participants. The requirement for written informed consent was waived by the IRB. All data were anonymized prior to the analysis to ensure patient confidentiality and data protection*.”

In this study, we used a secondary dataset from the aforementioned primary study that was approved by the Chief Investigator. We also justify an informed consent waiver for secondary data analysis and disclose funding sources and any conflicts of interest to ensure transparency regarding potential influences on study design, analysis, or interpretation, as needed [*References 2-4 below*]. The dataset was protected with a password, which only the principal investigator could access. The dataset was de-identified when it was transferred to the statistician for analysis.

Funding: This study did not receive any funding.

**References**

[1] Nguyen Tat T, Vo Hoang-Thien N, Nguyen Tat D, Nguyen PH, Ho LT, Doan DH, et al. Prognostic values of serum lactate-to-bicarbonate ratio and lactate for predicting 28-day in-hospital mortality in children with dengue shock syndrome. Medicine (Baltimore). 2024 ;103(17):e38000. doi: 10.1097/MD.0000000000038000. PMID: 38669370; PMCID: PMC11049702.

[2] Tripathy JP. Secondary Data Analysis: Ethical Issues and Challenges. Iran J Public Health. 2013 Dec;42(12):1478-9. PMID: 26060652; PMCID: PMC4441947.

[3] Research involving the secondary use of existing data. University of Berkeley, California. Available: <https://cphs.berkeley.edu/secondarydata.pdf>

[4] Waivers of Informed Consent Guidelines. Human Research Protection Program, University of Michigan. Available: <https://hrpp.umich.edu/irb-health-sciences-and-behavioral-sciences-hsbs/informed-consent-guidelines-templates/waivers-of-informed-consent-guidelines/>

**13. Language & Style —** Edit for clarity and standard English, correcting typographical issues such as “Shapley Addictive Explanations” to “Shapley Additive Explanations” and “admisison” to “admission”; define all abbreviations at first mention and use them consistently thereafter (DSS, LAR, L/B, MV, PALF, SHAP, RF, LR, SVM); standardize numerical reporting (e.g., p = 0.003 or p < 0.001, consistent AUC precision, en-dashes for ranges, n/N [%]); and shorten long sentences in the Methods and Discussion to improve readability.

**Response:** Thank you for your comment. We have made appropriate amendments in the revision manuscript submitted.

**Reviewer #2:**

Thank you for the chance to review a manuscript title “Comparison of serum lactate and lactate-derived ratios as prognostic biomarkers in pediatric dengue shock syndrome using supervised machine learning models” by Nguyen Tat Thanh and Vo Thanh Luan. I believe this work is valuable since lactate and related ratios are biochemically linked to the core pathophysiology of dengue shock syndrome in dengue infection. I have several suggestions that can be considered further by the authors; please find them below:

1. Per peer review best practice for title formatting, please avoid abbreviations in the title would enhance clarity for multidisciplinary readers—e.g., write “dengue shock syndrome” in full and avoid “ML” abbreviation.

**Response:** Thank you for this comment. We have made an amendment in the revised manuscript according to your suggestion. We have written the full term ML as machine learning. For dengue shock syndrome, the term DSS have been widely used and accepted.

2. In the abstract, lease explicitly address limitations or potential biases, which is crucial for biomarker performance claims.

**Response:** Thank you for your suggestion. Because of the very tight word limit (< 350 words) for the abstract in terms of the PLOS One journal format. Therefore, we aimed not to input your suggestions to state the study limitations or potential biases in the Abstract. Instead, we have added your suggestion to the Discussion section as appropriate.

3. To increase the reproducibility, it is better to explicitly mention parameter tuning details, data preprocessing steps, and handling of missing data (e.g., imputation method).

**Response:** Thank you for your comment. We have made appropriate amendments in the revision.

3.1  We have made an amendment to mention the parameter tuning details in the revised manuscript, as shown below: Hyperparameter tuning was performed for supervised models using a grid search strategy with 5-fold cross-validation to minimize overfitting and optimize generalizability. For the random forest, the number of trees, maximum tree depth, and minimum number of samples required for node splitting were tuned. For the support vector machine, the penalty parameter (C), kernel type, and kernel-specific parameters (e.g., gamma for radial basis function kernels) were adjusted. For the XGBoost model, the tuning included the learning rate, number of boosting iterations, maximum tree depth, and subsampling ratios. The optimal hyperparameters were selected based on cross-validated performance metrics.

3.2 The details of data preprocessing steps, submitted as a supplementary data **(S2 File)** below:

3.3 Handling of missing data (e.g., imputation method):
- Mechanism of missing data in this study: Missing at Random (MAR)
- To our best knowledge, there is no “black-and-white” principle to choose 20%, 30%... missingness threshold for exclusion rule.
- We determined the >20% missingness as threshold, mainly we want the robust data for machine learning analysis to ensure study power and validity of finding.
- For your suggestion, we additional perform a missingness heatmap and Pareto chart (shown below), which will be submitted in the revision as supplementary data:

**Handling of missing data**

The extent and pattern of missing data were presented with frequency and proportion (%) using Pareto chart (**S1 Fig**). Variables with more than 20% missingness were excluded. For the remaining features, multiple imputations were performed using **the missRanger package in R** (version 4.5.0), which implements random forest-based imputation **with predictive mean matching** [17]. The resulting imputed dataset was used for all machine-learning analyses.

**Reference**

[17]. Wright MN, Ziegler A. ranger: A Fast Implementation of Random Forests for High Dimensional Data in C++ and R. J. Stat. Soft. [Internet]. 2017; 77(1):1-17. Available from: <https://www.jstatsoft.org/index.php/jss/article/view/v077i01>. [cited 2025 July 29].

4. Add some potential bias considerations, such as being single-center and retrospective design.

**Response:** Thank you for your comment. We have made appropriate amendments in the revision manuscript submitted.

5. Kindly avoid overstating non-significant findings (no misleading p > 0.05 claims).

**Response:** Thank you for your comment and suggestion. We will make appropriate amendments to the revised manuscript.

6. The fact that L/B ratio underperformed may relate to its higher susceptibility to metabolic compensation and bicarbonate variability, but this reasoning is not discussed.

**Response:** Thank you for this suggestion. We have made appropriate amendments to the revised manuscript (paragraph 5 in **Discussion** section), as shown below.

The relatively modest prognostic performance of the LB ratio may be explained by its high susceptibility to physiological and clinical variability. While lactate levels rise consistently in response to tissue hypoperfusion, serum bicarbonate is more labile, reflecting not only metabolic acidosis, but also compensatory mechanisms, fluid resuscitation, and renal handling of acid–base balance. This variability may dilute the predictive signal of the LB ratio, making it less stable than lactate or the lactate-to-albumin alone. In contrast, the LAR integrates lactate with albumin, a marker less influenced by short-term metabolic fluctuations, thereby offering a more robust reflection of both metabolic stress and plasma leakage in severe dengue.

7. Calibration assessment (e.g., Hosmer-Lemeshow, Brier score) can be done and reported for prognostic model trustworthiness.

**Response:** Thank you for your suggestion. We further perform analyses of calibration analysis and plot in the revised manuscript.

7.1 Calibration plot: PR-AUC, Brier score, and expected calibration error with 95% CIs; include calibration-in-the-large and slope, add calibration plots.

**Response:** We have performed calibration analysis (**Table 4**) and input it in the revision.

**Table 4. Performance of predictive models with internal validation**

| Models | C-statistic | Calibration-in-the-large | Calibration slope | Brier score |
| --- | --- | --- | --- | --- |
| LAR | 0.96 (0.93) | 0 (-0.28) | 1 (0.72) | 0.03 (0.04) |
| Lactate | 0.96 (0.93) | 0 (-0.27) | 1 (0.74) | 0.03 (0.04) |
| LB | 0.95 (0.93) | 0 (-0.25) | 1 (0.76) | 0.04 (0.05) |

Statistics are summarized for training (test) sets performed with bootstrap (n =500 resampling) and internal validation. LAR, Lactate-to-albumin ratio; LB, Lactate-to-bicarbonate ratio

**Models**

- LAR-based full predictive model (*all covariates in supervised models*)
- Lactate-based full predictive model (*all covariates in supervised models*)
- LB-based full predictive model (*all covariates in supervised models*)

**Three calibration data above are combined in a single plot for visual evaluation.**

We can see that the lactate-based predictive model had a low Brier score (0.04) and better calibration plot than the LAR and LB models. Notably, the three models had mostly equivalent c-statistics and Brier scores. On the whole, the three models showed fairly good calibration.

8.Authors indeed have described LAR as “practical and accessible” but does not discuss feasibility in resource-limited settings. In many such contexts, albumin/bicarbonate assays and rapid turnaround are unavailable, and implementing complex ML models (e.g., RF, SVM) at the point of care is unrealistic without substantial infrastructure. Please narratively analyze the feasibility of implementing the assessment, especially in resource constraint setting.

**Response:** We are thankful for your comment. We believe that these biomarkers can be easily measured in the majority of hospitals, even in resource-limited settings. Additionally, we will design a convenient and user-friendly application integrated in an online web and/or smart phones for all clinicians to use at the bedside. In this respect, medical doctors do not need to remember all the complex machine-learning models.

\# Narratively analyze the feasibility of implementing the assessment, especially in resource constraint setting.

**Response:** We have input this amendment in the revised manuscript (in the last paragraph of the **Discussion** section).

“*These findings suggest that lactate-based biomarkers can be incorporated into routine triage protocols for children with severe dengue, particularly at resource-limited hospitals. Early identification of high-risk patients using such measures may support timely referral decisions and guide the prioritization of monitoring and interventions. However, their clinical utility should be externally validated in prospective studies across diverse healthcare settings before the widespread implementation*.”

**Reviewer #3:**

This manuscript addresses an important and timely clinical question regarding the prognostic utility of lactate-derived indices. However, there are several areas that require clarification, methodological strengthening, and refinement of presentation to ensure the work meets the standards. Below are detailed comments intended to help the authors enhance the scientific robustness
1. Please elaborate on how this work differs from previous publications, particularly the 2024 Medicine (Baltimore) study on the L/B ratio. While the Discussion notes that this is among the first pediatric DSS studies comparing LAR and L/B ratio with ML, stating this clearly in the Introduction would highlight the unique contribution.

**Response:** Thank you for this comment. Our research team reported the prognostic value of the LB ratio in predicting the risk of death among children with dengue shock syndrome (DSS) Medicine (Baltimore) in 2024. This study was restricted by the relatively small number of deaths (primary outcome) and the low proportion of mortality among DSS patients (approximately 5 %), which could potentially influence the study power and validity of the findings. Therefore, we propose a new approach for creating a composite endpoint of death, mechanical ventilation, dengue PALF, and encephalitis. These major dengue complications have been reported to have a high mortality rate among children with DSS. In this respect, the statistical power can be significantly enhanced.

Recently, we have further recognized the applicability of LAR in critically ill patients and we  determined to apply the LAR to our cohort of patients with severe dengue. A literature review showed no comparison between LAR, LB, and lactate. Hence, we combined all of these research gaps to conduct this study.

We are thankful for your suggested point of stating “*This is among the first pediatric DSS studies comparing LAR and L/B ratio with machine learning*”. This is an interesting idea considering the methodological and knowledge gaps (machine learning analysis and LAR vs. LB comparison). However, we are cautious about the strong statement in the Introduction, and we think it is better to place this statement in the Discussion section, considering that it is more appropriate.

2. The reported AUCs are high (up to 0.97), which raises concerns about overfitting. It would strengthen the manuscript to:
- Clarify whether nested cross-validation was used for hyperparameter tuning, and whether outcome stratification was applied to balance rare events.
- Add calibration metrics (e.g., Brier score, calibration plots) to complement discrimination measures.
- Describe steps taken to avoid data leakage during preprocessing (e.g., imputation and scaling performed within CV folds).

**Response:** Yes, you are correct in raising concerns about overfitting, regarding AUCs of 0.97 from conventional logistic analysis. We appreciate your suggestions for additional analyses and clarification of the calibration metrics (e.g., Brier score, calibration plots), data processing, and nested cross-validation used for hyperparameter tuning.

2.1 We used nested cross-validation across the analysis. We will input more clarification about hyperparameter tuning in the revision, as shown below:

Hyperparameter tuning was performed for supervised models using a grid search strategy with 5-fold cross-validation to minimize overfitting and optimize generalizability. For the random forest, the number of trees, maximum tree depth, and minimum number of samples required for node splitting were tuned. For the support vector machine, the penalty parameter (C), kernel type, and kernel-specific parameters (e.g., gamma for radial basis function kernels) were adjusted. For the XGBoost model, the tuning included the learning rate, number of boosting iterations, maximum tree depth, and subsampling ratios. The optimal hyperparameters were selected based on cross-validated performance metrics.

Considering rare outcome events (deaths), we aimed to combine all dengue-related complications to enhance statistical power. Additionally, we also have a suggestion from other peer reviewers of performing univariable associations of LAR, lactate, and L/B with each component of the study outcomes, as shown below. These tables will be submitted as Supplementary Data files in the revised manuscript.

**Table 1. The unadjusted associations of biomarkers and composite endpoint**

| *Composite clinical endpoint* | | | |
| --- | --- | --- | --- |
| **Variables** | **Odds ratio** | **95% CI** | **p-value** |
| LAR (+ 1 unit) | 2.84 | 2.14 – 3.78 | < 0.001 |
| Lactate (+1 mmol/L) | 1.54 | 1.36 – 1.74 | < 0.001 |
| LB (+ 1 unit) | 2.05 | 1.23 – 3.41 | < 0.01 |

**Table 2. The unadjusted associations of biomarkers and in-hospital mortality**

| *In-hospital mortality* | | | |
| --- | --- | --- | --- |
| **Variables** | **Odds ratio** | **95% CI** | **p-value** |
| LAR (+ 1 unit) | 1.82 | 1.5 – 2.21 | < 0.001 |
| Lactate (+1 mmol/L) | 1.56 | 1.38 – 1.77 | < 0.001 |
| LB (+ 1 unit) | 3.1 | 1.63 – 5.88 | < 0.001 |

**Table 3. The unadjusted associations of biomarkers and for mechanical ventilation**

| *Requirement for mechanical ventilation support* | | | |
| --- | --- | --- | --- |
| **Variables** | **Odds ratio** | **95% CI** | **p-value** |
| LAR (+ 1 unit) | 2.62 | 2 – 3.43 | < 0.001 |
| Lactate (+1 mmol/L) | 1.52 | 1.35 – 1.71 | < 0.001 |
| LB (+ 1 unit) | 2.08 | 1.24 – 3.47 | < 0.01 |

**Table 4. The unadjusted associations of biomarkers and dengue-associated acute liver failure (PALF)**

| *Dengue-associated PALF* | | | |
| --- | --- | --- | --- |
| **Variables** | **Odds ratio** | **95% CI** | **p-value** |
| LAR (+ 1 unit) | 1.88 | 1.54 – 2.3 | < 0.001 |
| Lactate (+1 mmol/L) | 1.52 | 1.35 – 1.7 | < 0.001 |
| LB (+ 1 unit) | 2.51 | 1.42 – 4.44 | < 0.01 |

Notably, only 4 patients were diagnosed with dengue-associated encephalitis, which has been reported to have poor clinical and survival outcomes. Due to the very limited number of encephalitis cases (4/524 patients), we did not perform univariable analysis between dengue-associated encephalitis and biomarkers (LAR, Lactate and LB), considering them to be too extreme in statistical calculations.

2.2 We have further performed the calibration analysis and plot in the revision, as shown below:

Calibration plot: PR-AUC, Brier score, and expected calibration error with 95% CIs; include calibration-in-the-large and slope, add calibration plots.

**Response:** We have performed calibration analysis and input in the revision (**Table 4**).

**Table 4. Performance of predictive models with internal validation**

| Models | C-statistic | Calibration-in-the-large | Calibration slope | Brier score |
| --- | --- | --- | --- | --- |
| LAR | 0.96 (0.93) | 0 (-0.28) | 1 (0.72) | 0.03 (0.04) |
| Lactate | 0.96 (0.93) | 0 (-0.27) | 1 (0.74) | 0.03 (0.04) |
| LB | 0.95 (0.93) | 0 (-0.25) | 1 (0.76) | 0.04 (0.05) |

Statistics are summarized for training (test) sets performed with bootstrap (n =500 resampling) and internal validation. LAR, Lactate-to-albumin ratio; LB, Lactate-to-bicarbonate ratio

**Models**

- LAR-based full predictive model (*with all covariates from supervised models*)
- Lactate-based full predictive model (*with all covariates from supervised models*)
- LB-based full predictive model (*with all covariates from supervised models*)

Combining three calibration plots above in a single figure for visual evaluation:

We can see that lactate-based predictive model had a low Brier score (0.04) and better calibration plot and, than LAR and LB models. Notably, the three models had mostly equivalent c-statistics and Brier scores. Overall, the three models exhibited fairly good calibration plots. These results support the use of the full model in supervised machine learning analysis.

2.3 Describe steps taken to avoid data leakage during preprocessing (e.g., imputation and scaling performed within CV folds).

The details of data preprocessing steps, submitted as supplementary data **(S2 File)** below:

Handling of missing data (e.g., imputation method):

- Mechanism of missing data in this study: Missing at Random (MAR)
- To the best of our knowledge, there is no “black-and-white” principle to choose the 20%, 30%... missingness threshold for the exclusion rule. We determined the >20% missingness as threshold, mainly we want the robust data for machine learning analysis to ensure study power and validity of finding.
- Multiple imputation method: predictive mean matching (pmm)

**Handling of missing data**

The extent and pattern of missing data were presented as frequency and proportion (%) using a Pareto chart (**S1 Fig**). Variables with more than 20% missingness were excluded. For the remaining features, multiple imputations were performed using **the missRanger package in R** (version 4.5.0), which implements random forest-based imputation **with predictive mean matching** [17]. The resulting imputed dataset was used for all machine-learning analyses.

**Reference**

[17]. Wright MN, Ziegler A. ranger: A Fast Implementation of Random Forests for High Dimensional Data in C++ and R. J. Stat. Soft. [Internet]. 2017; 77(1):1-17. Available from: <https://www.jstatsoft.org/index.php/jss/article/view/v077i01>. [cited 2025 July 29].

3. The composite outcome (mortality, MV, PALF, encephalitis) is clinically meaningful, but the rationale for combining these different endpoints should be explained in more detail, as their prognostic weight and pathophysiology differ. If feasible, a sensitivity analysis using mortality alone or analyzing each component separately would help confirm robustness.

**Response:** We are thankful for your insightful comments. We answer from point to point in the following.

3.1 The composite outcome (mortality, MV, PALF, encephalitis) is clinically meaningful, but the rationale for combining these different endpoints should be explained in more detail, as their prognostic weight and pathophysiology differ

**Response:** We have discussed the pathophysiology of each biomarker (serum albumin, lactate, and bicarbonate) in the Discussion section of the submitted manuscript.

**Albumin**: Serum albumin serves as a surrogate marker for the severity of plasma leakage, which is a hallmark of dengue pathogenesis. Given its biological half-life of approximately three weeks, a rapid and profound decline in albumin levels in the acute phase of DSS cannot be attributed solely to impaired hepatic synthesis or increased catabolism.

**Lactate**: Serum lactate, the numerator of LAR, indicates the metabolic consequences of plasma leakage, including impaired perfusion, anaerobic metabolism, and systemic circulatory failure. In DSS, elevated lactate levels not only signal the presence of shock but also reflect inadequate tissue oxygenation despite fluid resuscitation and vasopressor support.

**Bicarbonate:** In patients with severe dengue, serum bicarbonate is associated with the development of metabolic acidosis resulting from dengue shock syndrome, hypovolemia, tissue hypoperfusion, and anaerobic metabolism.

All these biomarkers indicate the severity of dengue and their appropriateness in dengue pathophysiology. Importantly, the LAR and LB ratios have previously been reported to have high predictive values in several critically ill cohorts. Therefore, the applicability of lactate-based ratios (LAR and LB) in a severe dengue cohort is sensible, considering the pathophysiological similarities.

3.2 If feasible, a sensitivity analysis using mortality alone or analyzing each component separately would help confirm robustness.

**Response:** This study should be interpreted with reference to our previous publications from to 2022-2024 [*see reference list 1-4 below*]. In our previous reports, we performed mathematical modelling of severe dengue cohorts using mortality alone and analyzing each component of the outcome separately.

\# **Using mortality alone**

In the published report “Machine learning-based models for prediction of in-hospital mortality in patients with dengue shock syndrome” [*reference 1*], we used in-hospital mortality as the primary outcome. Owing to the low number of deaths, we applied the SMOTE upsampling method aiming to improve the statistical power. The results of this study are consistent with those of our submitted manuscript. Nevertheless, we would like to use real-world data by combining critical dengue outcomes into a single composite endpoint to enhance the statistical power.

**# Analyzing each component of outcome separately**

Actually, we have analyzed each components of outcome with modelling methods and published these data previously [*references 2-4*]. The restricted number of outcome events was the main limitation to the validity of the study findings. Hence, it is sensible to use the composite endpoint in this study.

***References:***

[1] Vo LT, Vu T, Pham TN, Trinh TH, Nguyen TT. Machine learning-based models for prediction of in-hospital mortality in patients with dengue shock syndrome. World J Methodol 2025; 15(3): 101837 [DOI: [10.5662/wjm.v15.i3.101837](https://dx.doi.org/10.5662/wjm.v15.i3.101837)]

[2] Nguyen Tat T, Vo Hoang-Thien N, Nguyen Tat D, Nguyen PH, Ho LT, Doan DH, et al. Prognostic values of serum lactate-to-bicarbonate ratio and lactate for predicting 28-day in-hospital mortality in children with dengue shock syndrome. Medicine (Baltimore). 2024 Apr 26;103(17):e38000. doi: 10.1097/MD.0000000000038000. PMID: 38669370; PMCID: PMC11049702.

[3] Thanh NT, Luan VT, Viet DC, Tung TH, Thien V. A machine learning-based risk score for prediction of mechanical ventilation in children with dengue shock syndrome: A retrospective cohort study. PLoS One. 2024 Dec 6;19(12):e0315281. doi: 10.1371/journal.pone.0315281. PMID: 39642139; PMCID: PMC11623794.

[4]. Nguyen TT, Ngo PT, Vo LT. Predicting the risk of mortality in children with dengue-induced hepatitis admitted to the paediatric intensive care unit. World J Crit Care Med. 2024 Dec 9;13(4):98862. doi: 10.5492/wjccm.v13.i4.98862. PMID: 39655306; PMCID: PMC11577541.

4. The lack of 24-hour post-admission albumin data is acknowledged, but the discussion could be expanded to consider how serial lactate and albumin measurements might improve prognostic accuracy, referencing existing literature where available.

**Response:** Thank you for this comment. To the best of our knowledge, there are very limited data about serial lactate and albumin within 24h of admission in predicting poor outcomes in patients with severe dengue (although literature is available in other critically ill cohorts).

Recently, we published a cohort of LB ratio used to predict the risk of mortality in children with dengue shock syndrome. The 24h post-PICU admission lactate showed prognostic value for death [*reference* 1]. Another study showed serum albumin within the first 3 days of disease onset as a prognostic indicator of the progression of severe dengue in 2022 [*reference* 2].

We completely agree with the reviewer that 24h dynamic modelling of serum lactate and albumin levels in patients with severe dengue is important for improving prognostic accuracy. Based on this suggestion, we have included an additional discussion in the revised manuscript (Paragraph 6).

***Reference***

[1] Nguyen Tat T, Vo Hoang-Thien N, Nguyen Tat D, Nguyen PH, Ho LT, Doan DH, et al. Prognostic values of serum lactate-to-bicarbonate ratio and lactate for predicting 28-day in-hospital mortality in children with dengue shock syndrome. Medicine (Baltimore). 2024 Apr 26;103(17):e38000. doi: 10.1097/MD.0000000000038000. PMID: 38669370; PMCID: PMC11049702.

[2] Huy BV, Toàn NV. Prognostic indicators associated with progresses of severe dengue. PLoS One. 2022 Jan 5;17(1):e0262096. doi: 10.1371/journal.pone.0262096. PMID: 34986174; PMCID: PMC8730386.

5. In Table 3, please include 95% confidence intervals for sensitivity, specificity, and accuracy, similar to those provided for AUC. This would improve the statistical transparency of the results.

**Response:** Thank you for this comment. We have made appropriate amendments as per your suggestion in the revised manuscript.

**Revised Table.** Area under the receiver operating characteristic curve for serum lactate, lactate-to-albumin and lactate-to-bicarbonate ratios, and cut-off points for composite clinical endpoint among children with dengue shock syndrome

| Parameters | AUC of composite clinical endpoint (95% CI) | | p*-*value | |
| --- | --- | --- | --- | --- |
| Lactate (mmol/L) | 0.72 | 0.65 - 0.78 | < 0.001 | |
| LAR | 0.82 | 0.76 - 0.87 | < 0.001 | |
| L/B ratio | 0.68 | 0.62 - 0.74 | < 0.01 | |
| Cutoff points | **Sensitivity** | **Specificity** | **LR (+)** | **LR (-)** |
| LAR ≥ 1.25 | 0.71  (0.60 – 0.80) | 0.82  (0.78 – 0.86) | 3.95  (3.35 – 4.65) | 0.36  (0.29 – 0.44) |
| L/B ratio ≥ 0.20 | 0.56  (0.45 – 0.67) | 0.75  (0.71 – 0.79) | 2.26  (1.84 – 2.79) | 0.58  (0.47 – 0.72) |

AUC, Area under the curve; LR, Likelihood ratio, 95% CI, Confidence interval (AUC, sensitivity, specificity, LR+, LR-); LAR, Lactate-to-albumin ratio; L/B, Lactate-to-bicarbonate ratio

6. A brief discussion on applicability in non-tertiary or different regional settings would help contextualize the findings. In addition, note the potential selection bias introduced by excluding patients with >20% missing data.

**Response:** We are thankful for these comments, and we have made appropriate amendments as per your suggestion in the revised manuscript.

6.1 A brief discussion on applicability in non-tertiary or different regional settings would help contextualize the findings.

**Response:** We will input this brief discussion into the revised manuscript (Paragraph 8).

“These findings suggest that lactate-based biomarkers can be incorporated into routine triage protocols for children with suspected severe dengue, particularly in resource-limited provincial hospitals. Early identification of high-risk patients using such measures could support timely referral decisions and guide the prioritization of monitoring and interventions. However, their clinical utility should be further validated in prospective studies across diverse healthcare settings before their widespread implementation.”

6.2 In addition, note the potential selection bias introduced by excluding patients with >20% missing data.

We acknowledge the potential selection bias introduced by excluding patients with >20% of missing data. To the best of our knowledge, there is no “black-and-white” principle to choose 20%, 30%, etc., of the missingness threshold for the exclusion rule. The rule of thumb principle attempts to retain the best sample size for the statistical analysis. Based on the fact that we mainly want robust data for machine learning analysis to ensure the study power and validity of findings, we determined the >20% missingness as the threshold.

**Reviewer #4:**

**Major Comments:**
1. Consider including external validation or additional internal validation (e.g., bootstrap) to reduce the risk of overfitting.

**Response:** We are thankful for your comment. Per your suggestion, we have further performed internal validation with bootstrapping (n = 500 resampling) in the revised manuscript. The calibration analysis is presented below.

Calibration plot: The PR-AUC, Brier score, and expected calibration error with 95% CIs, including calibration-in-the-large and slope, and calibration plots have been added.

**Response**: We have performed calibration analysis and input in the revision (**Table 4**).

**Table 4. Performance of predictive models with internal validation**

| Models | C-statistic | Calibration-in-the-large | Calibration slope | Brier score |
| --- | --- | --- | --- | --- |
| LAR | 0.96 (0.93) | 0 (-0.28) | 1 (0.72) | 0.03 (0.04) |
| Lactate | 0.96 (0.93) | 0 (-0.27) | 1 (0.74) | 0.03 (0.04) |
| LB | 0.95 (0.93) | 0 (-0.25) | 1 (0.76) | 0.04 (0.05) |

Statistics are summarized for training (test) sets performed with bootstrap (n =500 resampling) and internal validation. LAR, Lactate-to-albumin ratio; LB, Lactate-to-bicarbonate ratio

**Models**

- LAR-based full predictive model (*all covariates in supervised models*)
- LB-based full predictive model (*all covariates in supervised models*)
- Lactate-based full predictive model (*all covariates in supervised models*)

Three calibration plots are combined in a single figure for visual evaluation.

We can see that lactate-based predictive model had a low Brier score (0.04) and better calibration plot and, than LAR and LB models. Notably, three models have mostly equivalent c-statistics and Brier score. On the whole, the calibration plots show a fairly good result, supporting the full model to be further analyzed in the supervised machine learning models.

2. Clarify the method for determining optimal cut-offs for LAR and L/B ratio.

**Response:** We used the Youden index to determine the optimal cut-offs for the LAR and L/B ratio. We have made this amendment in the revised manuscript for clarification.

3. Discuss the potential utility of these biomarkers in earlier clinical settings (e.g., ER triage) beyond PICU admission.

**Response:** Thank for this comment. We have made an amendment to your suggestion, as shown below.

“These findings suggest that lactate-based biomarkers can be incorporated into routine triage protocols for children with suspected severe dengue, particularly in resource-limited provincial hospitals. Early identification of high-risk patients using such measures could support timely referral decisions and guide the prioritization of monitoring and interventions. However, their clinical utility should be further validated in prospective studies across diverse healthcare settings before their widespread implementation.”

We have input this amendment in the **Discussion** section (Paragraph 8) in the revised manuscript.

4. Provide more detail on missing data patterns and imputation procedures, including sensitivity analyses comparing imputed vs. complete cases.

**Response:** We are thankful for this comment. Per your suggestion, we have provided more details on the missing data patterns and imputation procedures.

Mechanism of missing data in this study: Missing at Random (MAR)

To the best of our knowledge, there is no “black-and-white” principle to choose missingness thresholds of 20%, 30%...etc

We determined the >20% missingness as a threshold; we mainly wanted robust data for machine learning analysis to ensure study power and validity of the findings.

Based on your suggestion, we have added a missingness heatmap and Pareto chart (shown below), which will be submitted in the revision as supplementary data **(S1 Fig)**:

**Handling of missing data**

The extent and pattern of missing data were presented with frequency and proportion (%) using Pareto chart (**S1 Fig**). Variables with more than 20% missingness were excluded. For the remaining features, multiple imputations were performed using **the missRanger package in R** (version 4.5.0), which implements random forest-based imputation **with predictive mean matching** [17]. The resulting imputed dataset was used for all machine-learning analyses.

**Reference**

[17]. Wright MN, Ziegler A. ranger: A Fast Implementation of Random Forests for High Dimensional Data in C++ and R. J. Stat. Soft. [Internet]. 2017; 77(1):1-17. Available from: <https://www.jstatsoft.org/index.php/jss/article/view/v077i01>. [cited 2025 July 29].

- We have stated this information in the submitted manuscript.

\# Suggestion for the sensitivity analyses comparing imputed vs. complete cases.

To the best of our knowledge, mathematical modelling with multiple imputation dataset has been evidenced and highly recommended by statistical experts for its robustness [*References 1-4 below]*. Therefore, we would like not to perform sensitivity analyses comparing imputed vs. complete cases. Indeed, we are thankful for your suggestion.

**References**

[1] Austin PC, White IR, Lee DS, van Buuren S. Missing Data in Clinical Research: A Tutorial on Multiple Imputation. Can J Cardiol. 2021 Sep;37(9):1322-1331. doi: 10.1016/j.cjca.2020.11.010. Epub 2020 Dec 1. PMID: 33276049; PMCID: PMC8499698.

[2] Hughes RA, Heron J, Sterne JAC, Tilling K. Accounting for missing data in statistical analyses: multiple imputation is not always the answer. Int J Epidemiol. 2019 Aug 1;48(4):1294-1304. doi: 10.1093/ije/dyz032. PMID: 30879056; PMCID: PMC6693809.

[3] Sinharay S, Stern HS, Russell D. The use of multiple imputation for the analysis of missing data. Psychol Methods. 2001 Dec;6(4):317-29. PMID: 11778675.

[4] Lüdtke O, Robitzsch A, Grund S. Multiple imputation of missing data in multilevel designs: A comparison of different strategies. Psychol Methods. 2017 Mar;22(1):141-165. doi: 10.1037/met0000096. Epub 2016 Sep 8. PMID: 27607544.

5. Expand on the feasibility and cost-effectiveness of implementing LAR in low-resource settings.

**Response:** Thank you for this comment. We have also performed decision curve analysis as per your suggestion. Decision curve analysis is a helpful to evaluate cost-effectiveness among predictive models, particularly in design intervention schemes in limited-resource settings. We make this amendment in the revision manuscript resubmitted.

\# Decision Curve Analysis for 3 models: LAR/LB/Lactate based models:

Decision curve analysis demonstrated that application of the models for predicting critical dengue-related outcomes provided a consistent net clinical benefit across a wide range of threshold probabilities for LAR, L/B, and lactate models. These findings indicate that such predictive approaches may have meaningful clinical applicability and utility in the management of severe dengue, particularly in resource-limited settings.

In addition, these findings suggest that lactate-based biomarkers can be incorporated into routine triage protocols for children with suspected severe dengue, particularly in resource-limited provincial hospitals. Early identification of high-risk patients using such measures could support timely referral decisions and guide the prioritization of monitoring and interventions. However, their clinical utility should be further validated in prospective studies across diverse healthcare settings before their widespread implementation.

**Minor Comments:**
1. Add definitions for all abbreviations at first mention.

**Response:** Thank for this comment. We make appropriate amendments in the revised manuscript.

2. Include 95% CI for all metrics in Table 3 for consistency.

**Response:** Thank for this comment. We make appropriate amendments in the revised manuscript, as shown below.

**Revised Table.** Area under the receiver operating characteristic curve for serum lactate, lactate-to-albumin and lactate-to-bicarbonate ratios, and cut-off points for composite clinical endpoint among children with dengue shock syndrome

| Parameters | AUC of composite clinical endpoint (95% CI) | | p*-*value | |
| --- | --- | --- | --- | --- |
| Lactate (mmol/L) | 0.72 (0.65 - 0.78) | | < 0.001 | |
| LAR | 0.82 (0.76 - 0.87) | | < 0.001 | |
| L/B ratio | 0.68 (0.62 - 0.74) | | < 0.01 | |
| Cutoff points | **Sensitivity** | **Specificity** | **LR (+)** | **LR (-)** |
| LAR ≥ 1.25 | 0.71  (0.60 – 0.80) | 0.82  (0.78 – 0.86) | 3.95  (3.35 – 4.65) | 0.36  (0.29 – 0.44) |
| L/B ratio ≥ 0.20 | 0.56  (0.45 – 0.67) | 0.75  (0.71 – 0.79) | 2.26  (1.84 – 2.79) | 0.58  (0.47 – 0.72) |

AUC, Area under the curve; LR, Likelihood ratio, 95% CI, Confidence interval (AUC, sensitivity, specificity, LR+, LR-); LAR, Lactate-to-albumin ratio; L/B, Lactate-to-bicarbonate ratio

3. Improve labeling and color contrast in SHAP plots for readers unfamiliar with the method.

**Response:** Thank for this comment. We make appropriate amendments in the revised manuscript.

4. Address minor grammatical issues during copy-editing.

**Response:** Thank for this comment. We make appropriate amendments in the revised manuscript.

We have answered all the questions by all the reviewers and have offered corrections as needed. We are thankful for your significant comments that have made our study more comprehensive to the readers.

We look forward to your feedback and answer any further questions.

Many thanks,

Best regards,

Dr Thanh Nguyen Tat
