# Supplementary material for: Comparison of serum lactate and lactate-derived ratios as prognostic biomarkers in pediatric dengue shock syndrome using supervised machine learning models
Source: PLoS One. 2025 Oct 27;20(10):e0335022. doi: 10.1371/journal.pone.0335022 (PMC12558473; doi:10.1371/journal.pone.0335022)
Supplement: Attachment — Submitted filename: Response_to_PLOS_Editors_auresp_2.docx [file pone.0335022.s014.docx]

**Response letter to the journal editors**

Dear Editors-in-Chief of the PLOS ONE,

We are very thankful for your comments on our submitted manuscript.

**MANUSCRIPT #**	PONE-D-25-41174R1

“Comparison of serum lactate and lactate-derived ratios as prognostic biomarkers in pediatric dengue shock syndrome using supervised machine learning models”

These are our responses to all the reviewers’ comments.

**Journal Requirements:**

1. If the reviewer comments include a recommendation to cite specific previously published works, please review and evaluate these publications to determine whether they are relevant and should be cited. There is no requirement to cite these works unless the editor has indicated otherwise.

**Response:** Thank you for this comment. We have made appropriate amendments in the revised manuscript.

We have meticulously looked through the previously reference list and determined to remove these cited references, in terms of less relevance, as follows:

World Health Organization‎. Global strategy for dengue prevention and control 2012-2020. World Health Organization, 2012. [Accessed and cited on 04th December 2023]. Available from: <https://iris.who.int/handle/10665/75303>

Nguyen TT, Nguyen DT, Vo TT, Dang OT, Nguyen BT, Pham DT, et al. Associations of obesity and dengue-associated mortality, acute liver failure and mechanical ventilation in children with dengue shock syndrome. Medicine (Baltimore). 2023; 102(46):e36054. doi: 10.1097/MD.0000000000036054. PMID: 37986332; PMCID: PMC10659721

Nguyen TT, Le NT, Nguyen NM, Do VC, Trinh TH, Vo LT. Clinical features and management of children with dengue-associated obstructive shock syndrome: A case report. Medicine (Baltimore). 2022; 101(43):e31322. doi: 10.1097/MD.0000000000031322. PMID: 36316901

Vo LT, Vu T, Pham TN, Trinh TH, Nguyen TT. Machine learning-based models for prediction of in-hospital mortality in patients with dengue shock syndrome. World J Methodol. 2025; 15(3): 101837. [DOI: [10.5662/wjm.v15.i3.101837](https://dx.doi.org/10.5662/wjm.v15.i3.101837)]

2. Please review your reference list to ensure that it is complete and correct. If you have cited papers that have been retracted, please include the rationale for doing so in the manuscript text, or remove these references and replace them with relevant current references. Any changes to the reference list should be mentioned in the rebuttal letter that accompanies your revised manuscript. If you need to cite a retracted article, indicate the article’s retracted status in the References list and also include a citation and full reference for the retraction notice.

**Response:** Thank you for your comment. We have removed retracted articles from reference and replaced appropriate references as suggested.

We have carefully checked the retraction status of updated cited references in the revision, and we affirm that all cited references were checked for retraction or expressions of concern (via PubMed/PMC, publisher webpages and CrossRef on 14 September 2025; no retractions or expressions of concern have been identified under current submission).

3. “While revising your submission, please upload your figure files to the Preflight Analysis and Conversion Engine (PACE) digital diagnostic tool, [https://pacev2.apexcovantage.com/](https://track.editorialmanager.com/CL0/https:%2F%2Fpacev2.apexcovantage.com%2F/1/010f0199419a2505-8a46eb10-6e25-45bc-b165-9248c11361e1-000000/MybAdzh3PwU7XKoAVmfuNpuFFnnPLGg7-QgM0vMNCkI=228). PACE helps ensure that figures meet PLOS requirements.”

**Response:**

Because we are not familiar with using PACE, considering the journal’s suggestion, we kindly suggest the journal editors to do a favor to help our research team with this task, editing all figures to meer the journal requirement for publications. All the figures are now in 600 dpi format.

We request assistance in formatting the figures (width and length) to meet the journal’s standards.

We have made the appropriate amendments as per your suggestions.

We look forward to your feedback and answer any further questions.

Many thanks,

Best regards,

Dr. Nguyen Tat Thanh
