# Supplementary material for: Comparison of serum lactate and lactate-derived ratios as prognostic biomarkers in pediatric dengue shock syndrome using supervised machine learning models
Source: PLoS One. 2025 Oct 27;20(10):e0335022. doi: 10.1371/journal.pone.0335022 (PMC12558473; doi:10.1371/journal.pone.0335022)
Supplement: Attachment — Submitted filename: Response to Reviewers -27Sep2025.docx [file pone.0335022.s015.docx]

**Response letter to reviewers**

Dear Editors-in-Chief of the PLOS ONE,

We are very thankful for your insightful comments for our submitted manuscript.

MANUSCRIPT # **PONE-D-25-41174**, titled "Comparison of serum lactate and lactate-derived ratios as prognostic biomarkers in pediatric dengue shock syndrome using supervised machine learning models"

We are thankful for all peer-review points. These are our responses to all the reviewers’ comments.

**Journal Editors:**

Please carefully consider all comments from the reviewers. I agree with Reviewer 4 that the limitation paragraph can still benefit of further elaboration, especially considering the data collection method.

In addition, kindly revise the "data availability" statement and cite the primary data source: <https://journals.plos.org/plosone/article?id=10.1371/journal.pone.0126134>

**Response:** We are thankful for your comments. We have made appropriate amendments according to your suggestions.

**Data availability**: All relevant data are within the paper and its Supporting Information files.

**Our primary data source:**

All relevant data supporting the findings of this study are available within the paper, its Supporting Information files, and the primary dataset from the parent study: Nguyen Tat T, Vo Hoang-Thien N, Nguyen Tat D, Nguyen PH, Ho LT, Doan DH, et al. Prognostic values of serum lactate-to-bicarbonate ratio and lactate for predicting 28-day in-hospital mortality in children with dengue shock syndrome. Medicine (Baltimore). 2024;103(17):e38000. doi: 10.1097/MD.0000000000038000. PMID: 38669370; PMCID: PMC11049702. This dataset was approved by the Scientific Committee and Institutional Review Board of Children’s Hospital No.2, Ho Chi Minh City, Vietnam (IRB No. 893/QD-BVND2; 06 June 2022).

We have cited this reference as appropriate.

[4] Nguyen Tat T, Vo Hoang-Thien N, Nguyen Tat D, Nguyen PH, Ho LT, Doan DH, et al. Prognostic values of serum lactate-to-bicarbonate ratio and lactate for predicting 28-day in-hospital mortality in children with dengue shock syndrome. Medicine (Baltimore). 2024 ;103(17):e38000. doi: 10.1097/MD.0000000000038000. PMID: 38669370; PMCID: PMC11049702.

The limitation paragraph can still benefit of further elaboration, especially considering the data collection method.

**Response:** We thank for your comment. We have made appropriate amendments according to your suggestions in the revised manuscript (Lines 584-588).

“As this study was based on secondary data analysis, it was constrained by the scope and quality of the information collected in the original investigation, with no possibility of verifying or supplementing missing variables. Furthermore, the consistency of the recorded data may have been affected by variations in clinical practice and documentation during the study period.”

**Journal Requirements:**

1. If the reviewer comments include a recommendation to cite specific previously published works, please review and evaluate these publications to determine whether they are relevant and should be cited. There is no requirement to cite these works unless the editor has indicated otherwise.

**Response:** Thank you for your comment. We have strictly followed this recommendation.

2. Please review your reference list to ensure that it is complete and correct. If you have cited papers that have been retracted, please include the rationale for doing so in the manuscript text, or remove these references and replace them with relevant current references. Any changes to the reference list should be mentioned in the rebuttal letter that accompanies your revised manuscript. If you need to cite a retracted article, indicate the article’s retracted status in the References list and also include a citation and full reference for the retraction notice.

**Response:** Thank for your comment. We have strictly followed this recommendation. We have checked and no retracted articles have been found in the reference list.

**Reviewer #4:**

**Strengths:**
1. Important clinical relevance: pediatric DSS with high mortality risk.
2. Large cohort (524 patients) with comprehensive clinical and laboratory data.
3. Novelty: evaluation of lactate-derived ratios (LAR, LB) using machine learning, which is underexplored in pediatrics.
4. Robust methodology with multiple supervised models, SHAP interpretability, and decision curve analysis.
5. Clear finding that LAR outperforms lactate and LB ratio for prognostic prediction.

**Points for minor revision:**
1. Clarity of language: Some sentences in the Introduction and Discussion are long and complex. Please consider a professional language edit to improve readability for an international audience.
2. Logistic regression overfitting: In the Results, you note potential overfitting of LR. It would be useful to briefly explain how this may affect interpretation and why ensemble models are more reliable here.
3. Figures: You requested editorial assistance for figure formatting. If possible, ensure figure legends are fully self-explanatory, particularly for Fig 2 (RF variable importance) and Fig 3 (SHAP summary plot).
4. Limitations: The Discussion could be slightly expanded to emphasize that this is a single-center retrospective analysis, and that external validation in independent pediatric cohorts is necessary.
5. Clinical applicability: While the statistical performance of LAR is strong, please comment on how this could be practically implemented in resource-limited DSS treatment settings.
Overall, this is a strong and clinically impactful paper. With minor polishing, it will make a valuable contribution.

**Response:**

We are thankful for the reviewers’ comments for making our paper more comprehensive to the readers. These are our responses to your comments.

1. Clarity of language: Some sentences in the Introduction and Discussion are long and complex. Please consider a professional language edit to improve readability for an international audience.

**Response:** We are thankful for your comments, and we have made appropriate amendments in the revised manuscript.

2. Logistic regression overfitting: In the Results, you note potential overfitting of LR. It would be useful to briefly explain how this may affect interpretation and why ensemble models are more reliable here.

**Response:** We are thankful for your comments. In our opinion, the Results section should mainly describe what the statistical analyses yield the results. In this section, it is not recommended to explain or discuss any thing about data results. Instead, explanation (or interpretation study results) should be placed in the Discussion section.

In this respect, we have discussed the logistic regression overfitting and showed that ensemble models are more reliable with appropriately cited references in the paragraph 4, Discussion section.

3. Figures: You requested editorial assistance for figure formatting. If possible, ensure figure legends are fully self-explanatory, particularly for Fig 2 (RF variable importance) and Fig 3 (SHAP summary plot).

**Response:** We are thankful for your comments. Appropriate figure legends have been provided in the revised manuscript as well. Notably, we aim to provide a succinct and clear description, and our target readers are mainly researchers with a good background in mathematical modelling.

*# Previous submission version*

[Fig 2 Caption]

**Fig 2. Comparisons of serum lactate levels, lactate-derived ratios, and significant clinical variables of the random forest model based on classification accuracy.**

*Revision*

**Fig 2. Key predictors of the composite clinical endpoint in dengue shock syndrome identified by the random forest model.**
The lactate-to-albumin ratio (LAR) was the strongest predictor, outperforming serum lactate and the lactate-to-bicarbonate ratio. Additional important variables included markers of hemodynamic compromise, organ dysfunction, and disease severity, reflecting the multifactorial pathophysiology of dengue shock syndrome.

*# Previous submission version*

[Fig 3 Caption]

**Fig 3. SHAP summary plot illustrating the relative contributions of serum lactate, lactate-derived ratios (LAR and LB), and clinical features to the prediction of critical outcomes in pediatric patients with dengue shock syndrome.**

*Revision*

**Fig 3. SHAP summary plot of predictors of critical outcomes in pediatric dengue shock syndrome.**
SHAP analysis identified elevated INR, platelet transfusion requirement, high fluid administration, and respiratory failure as the strongest clinical predictors, whereas LAR was the most informative biomarker, followed by LB ratio and serum lactate. These results emphasize the prognostic utility of serum lactate-derived indices and the importance of multiple clinical features in determining the outcomes.

4. Limitations: The Discussion could be slightly expanded to emphasize that this is a single-center retrospective analysis, and that external validation in independent pediatric cohorts is necessary.

**Response:** We did discuss these points and we have provided this information in our previous manuscript sunbmitted to the journal.

Lines 579-586, Paragraph 7, Discussion section

Lines 604-605, last sentences in the Paragraph 8 (last paragraph), Discussion section

We have clearly states these points in the previous submission. Importantly, the readers of this niche paper have a solid background in scientific research; hence, we believe there is no need to elaborate these information in so details. Thank you.

5. Clinical applicability: While the statistical performance of LAR is strong, please comment on how this could be practically implemented in resource-limited DSS treatment settings.

**Response:** We did discuss and provide this information in our previous manuscript sunbmitted to the journal in Paragraph 8 (last paragraph), Discussion section. Per your suggestion, we make a minor amendment for clarification in the revision (last paragraph, Discussion section).

*Revision*

This study highlights the potential of lactate-derived biomarkers, particularly the lactate-to-albumin ratio (LAR), as practical tools for early risk stratification in pediatric dengue shock syndrome. Compared with the lactate-to-bicarbonate (LB) ratio and serum lactate alone, LAR demonstrated superior prognostic performance in predicting critical complications, including mechanical ventilation, acute liver failure, and dengue-associated encephalitis. Importantly, both serum lactate and albumin tests are routinely available and low-cost laboratory measurements in most provincial and district hospitals, making the LAR feasible for implementation, even in resource-limited settings. Incorporating LAR into admission triage protocols may allow for the earlier identification of high-risk children, support timely referral to higher-level facilities, and guide the prioritization of intensive monitoring and therapeutic interventions. Nevertheless, prospective validation across diverse healthcare systems is needed to confirm its generalizability before it can be adopted as a standard risk assessment tool.

We have answered all the questions by all the reviewers and have offered corrections as needed. We are thankful for your significant comments that have made our study more comprehensive to the readers.

We look forward to your feedback and answer any further questions.

Many thanks,

Best regards,

Dr Thanh Nguyen Tat
